# Structure-Guided Design of a Subnanomolar Dengue Virus NS4B Inhibitor

**DOI:** 10.1021/acs.jcim.6c02098

**Published:** 2026-10-06

**Authors:** Poomipat Laorsatiankul, Mohammad Hassan Baig, Shuang Chen, Mehdi Hassan, Patan Rasvan Khan, Mala Ramanjaneyulu, Kim Chang Joong, Jo Yun Seong, Jae-June Dong, Shozeb Haider

**Affiliations:** † Pharmaceutical and Biological Chemistry, 371646 UCL School of Pharmacy, London WC1N 1AX, U.K.; ‡ 58920 BNJ Biopharma, Yeonsu-gu, Incheon 21983, Republic of Korea; § Department of Family Medicine, Kangbuk Samsung Hospital, Samsung Medical Center, Jongno District, Seoul 03181, Republic of Korea; ∥ Prince Fahad Bin Sultan Chair for Biomedical Research, University of Tabuk, Tabuk 71491, Saudi Arabia; ⊥ Department of Chemistry, University of Baltistan Skardu, Skardu 16100, Pakistan

## Abstract

Dengue virus nonstructural protein 4B (NS4B) is an essential regulator of viral replication but remains structurally unresolved, limiting drug discovery. Here, we combine modeling, ligand-based design, and experimental validation to enable structure-guided inhibition of NS4B. Comparative models across all four serotypes reveal a conserved transmembrane binding pocket consistent with resistance mutations. Docking of JNJ-series inhibitors defines key interaction features that guide the development of predictive field-based 3D-QSAR models (R = 0.91, Q = 0.89). Prospective screening of bioisosteric and in-house compounds identifies BNJ-495 as a candidate broad-spectrum inhibitor with predicted activity across all four dengue serotypes. Experimental validation confirms potent activity against dengue virus serotype 2 (IC_50_ = 0.17 nM) with a high selectivity index (∼37,000). Conserved binding interactions across serotypes support broad-spectrum potential. This study establishes a general framework for targeting structurally elusive viral membrane proteins.

## Graphical abstract

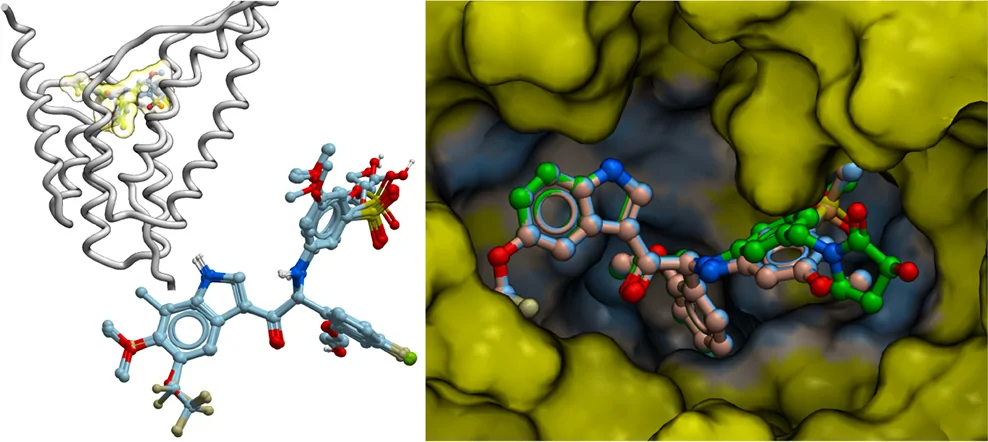

## Introduction

Dengue virus is a positive-sense (+) single-stranded RNA flavivirus that is transmitted through the bite of the female *Aedes* mosquito.^1,2^ It is estimated that around 400 million DENV infections happen annually worldwide, with an average of 500,000 people needing hospitalization for severe and fatal illnesses. Moreover, two and a half billion people live in areas at high risk of dengue, with mortality rates exceeding 5–20% in some areas.^3–5^ Dengue infection is therefore considered a severe public health risk worldwide (World Health Organization, 2012).^6^ Clinical manifestations range from mild dengue fever (DF) to the fatal symptoms, including dengue hemorrhagic fever (DHF) and dengue shock syndrome (DSS).^7^ Antibodies produced after infection with dengue fever are only immune to infections of the same dengue serotype.^5,8^ Some clinical studies suggest that infection with a heterologous serotype increases the risk of fatal symptoms of dengue fever.^9^

The dengue virus has four serotypes: DENV-1, DENV-2, DENV-3, and DENV-4. Each serotype is prevalent worldwide and can result in a wide variety of illnesses. The Dengue viral genome is a single-stranded RNA of approximately 11,000 base pairs in length. As viral replication begins, the open reading frame is translated into a polyprotein and subsequently cleaved by viral and cellular proteases into seven nonstructural proteins (NS1, NS2A, NS2B, NS3, NS4A, NS4B, and NS5) and three structural proteins (nucleocapsid C shell, prM/M premembrane/membrane, and envelope glycoprotein E; Figure 1A).^8^ These proteins are essential for the life cycle of the virus. Among the nonstructural proteins, NS4B plays an indispensable role in viral replication. Structurally, NS4B is a small transmembrane protein. Despite extensive biochemical and biophysical investigations, no high-resolution structure of NS4B has been reported. As a result, our understanding of NS4B has come from topology mapping and indirect biophysical characterization rather than a definitive 3D structure.^10,11^ Despite lacking enzymatic activity, it serves as a structural and organizational hub for the viral replication complex. By interacting with other nonstructural proteins, particularly NS3, NS4A, and the NS2B/NS3 protease–helicase complex, NS4B helps establish the specialized vesicle packets that house viral RNA synthesis. These membrane-bound replication organelles do not form effectively in its absence, highlighting NS4B’s critical role in shaping and maintaining the intracellular environment required for efficient genome replication.^10^

**Figure 1:**
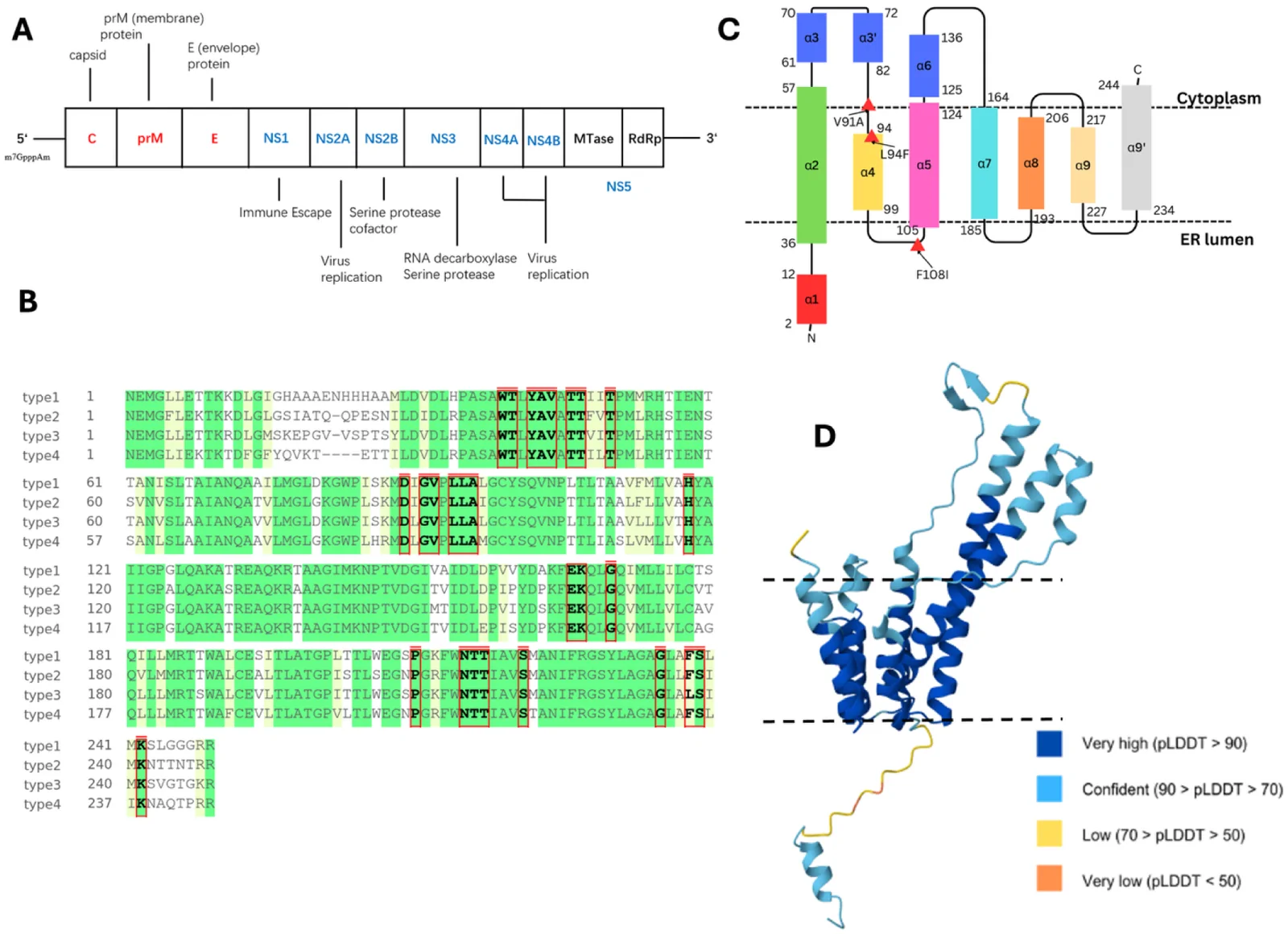
(A) Schematic representation of the Dengue virus genome organization. The polyprotein encodes structural proteins (C, prM, E; red) and nonstructural proteins (NS1–NS5; blue), with their primary biological roles indicated. (B) Sequence alignment of all four serotypes of the NS4B protein. Conserved residues are highlighted in green, while binding site residues are highlighted in bold and boxed in red. (C) Proposed structural topology of DENV-2 NS4B. Specific mutations, such as V91A and L94F, are indicated by red triangles. (D) AlphaFold-predicted 3D structure of DENV-2 NS4B. The model is color-coded by the predicted Local Distance Difference Test (pLDDT) score to indicate confidence levels. The dashed lines represent the boundaries of the membrane.

The importance of NS4B extends beyond its scaffolding functions. It also acts as a regulatory component of the replication machinery by interacting with NS3.^12^ Furthermore, NS4B significantly enhances the DNA unwinding activity of the NS3 helicase.^13–15^ NS4B exists not only in its mature form but also as part of the NS4A-2K-NS4B precursor, an intermediate that is functionally linked to the earliest steps of replication organelle biogenesis. Blocking the activity or processing of this intermediate interrupts viral RNA replication at its inception.^16^ Additionally, the NS4B protein is known to specifically disrupt STAT1 phosphorylation, hence mediating antagonism of the host’s innate immune response.^17^ Recent efforts to develop dengue antiviral inhibitors have identified the transmembrane protein NS4B as a particularly attractive candidate due to its function.^18^

In drug development, vaccines have proven difficult to produce a balanced immune response against all four dengue serotypes. An attenuated dengue vaccine CYD-TDV, or Dengvaxia, has been licensed in 20 countries under strict conditions and can only be administered to people who have previously been infected with dengue.^19,20^ Although numerous small-molecule compounds that inhibit DENV have been reported, none has been licensed for the treatment of dengue fever.^21,22^ Nevertheless, a few compounds have successfully advanced through clinical trials.^23^ Although the mechanism of inhibition is not fully understood, experimental results indicate binding to NS4B with nanomolar affinity across all four serotypes.^24^ A small molecule that did not reveal any safety issues in a clinical trial was JNJ-1802.^25^ It has been demonstrated that JNJ-1802 and NITD-688 (Figure S1) inhibit viral multiplication by blocking the interaction between the DENV nonstructural proteins NS3 and NS4B.^24,26^ Although JNJ-1802 showed the highest potency, the project was discontinued due to an internal company decision (JNJ.com, 2024). Another compound JNJ-A07 (Figure S1) binds selectively to NS4B or its precursor and blocks the interactions between NS4A-2K-NS4B and the NS2B/NS3 protease–helicase complex.^16^

Because of the lack of high-resolution structural information, all NS4B inhibitors reported to date have relied on ligand-based approaches rather than true structure-guided approaches. While ligand-based discovery has produced potent compounds, the absence of structural insight restricts our ability to optimize binding interactions, understand resistance mutations, or design broad-spectrum inhibitors across all dengue serotypes.

In this study, we model the NS4B protein structure and report on novel, high-potency NS4B inhibitors. Using the binding poses of JNJ-series compounds to NS4B, we constructed a 3D-QSAR model from the JNJ inhibitor library, which was then applied to predict the activity of new ligands and screen additional candidates. This structure-based optimization approach enabled the identification of key residues that define the NS4B binding site and the selection of the most promising inhibitors. Through this combined structural and computational workflow, our study provides an integrated approach for rationalized structure-based discovery of NS4B inhibitors.

## Methods

### Field-Based 3D-QSAR

#### Data Preparation

The 21 JNJ compounds with reported antiviral EC_50_ values across the four DENV serotypes were retrieved from a previously published study.^25^ The EC_50_ values, reported in μM, were transformed into negative logarithmic (pEC_50_) values, to ensure a normalized data distribution for predictive modeling, using the following formula:

$$p{\mathrm{EC}}_{50}=-\log_{10}\left({\mathrm{EC}}_{50}\right)$$

The SMILES codes of the compounds were compressed into SDF files for uploading and generating 3D structures in the ICM-Pro software v3.9-1b (www.molsoft.com).

#### Molecular Docking

The conservation of the amino acid sequence among all four DENV serotypes ranges between 76 and 82%, highlighting its functional importance as a dengue pan-serotype target (Figure 1B).^23^ In the absence of any experimental structure, the NS4B protein was built using AlphaFold 3 (serotype 1 NS4B protein strain Nauru/West Pac/1974 (UniProt ID: P17763), serotype 2 NS4B protein strain 16681-PDK53 (UniProt ID: P29991), serotype 3 NS4B protein strain Philippines/H87/1956 (UniProt ID: P27915), and serotype 4 NS4B protein strain Philippines/H241/1956 (UniProt ID: Q58HT7)). Docking was performed using the MolSoft ICM-Pro (ver 3.9-1b) stochastic global optimization algorithm.^27^ The PocketFinder function was used to search for the binding pockets. The selected binding site corresponded to the region containing residues V91, L94, and T108 in DENV-2. Substitutions at these residues have been shown to reduce the potency of JNJ-1802.^25^ This information was used to build a 30 Å^3^ grid that encompassed the residues along with the identified binding site.^28^ Ligands were treated as fully flexible during docking, while the receptor was kept rigid. Multiple docking runs were then performed to dock all JNJ ligands. 50 alternate conformations were generated to explore potential binding poses and evaluate scores. The final pose selected for molecular alignment was chosen based on the docking score, consistency with the experimentally identified resistance-associated residues (V91, L94, and T108), and similarity across the JNJ inhibitor series. The consensus orientation was then selected as a template for further docking of the in-house ligands, including the bioisosteres and BNJ ligands.

#### Molecular Alignment

Prior to building the 3D-QSAR model, the molecular alignment of the 21 JNJ ligands (Figure S1) was performed using ICM-Pro software. The ligands were superimposed based on the molecular structure, such as core residues (phenyl core, aniline core, and indole core), and physicochemical properties, such as electrostatic, hydrophobic, HBA, HBD, and aromatic ring interaction field potentials.

Stereochemistry was explicitly considered during modeling. JNJ-1802 has been reported as the S-enantiomer, and this configuration was used as the reference stereochemistry during docking and alignment. The same S-configuration was assigned to the corresponding stereogenic center in the JNJ, BIS, and BNJ compound series to ensure consistency in molecular alignment and 3D-QSAR model construction. The objective of the docking procedure was to generate a consistent binding orientation across the congeneric series for subsequent 3D-QSAR analysis rather than to establish an experimentally validated stereochemically resolved binding mode.

#### 3D-QSAR Model

A three-dimensional quantitative structure–activity relationship (3D-QSAR) model was constructed using Schrödinger Maestro (Schrödinger Release 2024-4). This model was used to establish a statistically significant correlation between the structural attributes of the ligands and their observed antiviral activity (EC_50_). We focused on EC_50_ (half-maximal effective concentration), which represents the concentration of the compound required to induce a 50% response. In this case, the concentration that reduces antiviral activity by 50%.^29^ The JNJ data set was utilized to develop the 3D-QSAR models using partial least-squares (PLS) regression analysis. To construct the 3D-QSAR models using the field-based QSAR tool, the extended Gaussian potential function, which comprises steric, electrostatic, hydrophobic, HBA, HBD, and aromatic ring interaction field potentials, was chosen. The maximum PLS factor was set to 3 to avoid overfit models. The random training set was set to 70%. The grid spacing was set to 1.0 Å. The force field values were set to ignore the force field within 2.0 Å of any training-set atom. The truncated steric and electrostatic force field was maintained at its default value of 30.0 kcal/mol. Variables with a standard deviation <0.01 were eliminated.

The number of ligands to leave out for cross-validation was set to 1 (leave-one-out). The models for each PLS factor were evaluated by using internal and external validation parameters, and the best 3D-QSAR model was selected on the basis of statistical robustness.^30^ This protocol was applied to all DENV serotypes of the NS4B protein. Then, analogues and in-house ligands were predicted to be active using the prediction function based on field-based 3D-QSAR. The ligands were ranked on the basis of their predicted antiviral activity.

### Bioisostere Generation

To increase the chemical diversity and improve the physicochemical properties of lead compounds, bioisosteres of the reference ligand were identified. JNJ-24 (JNJ-1802) was selected as the reference ligand. The bioisosteric replacement focused on the side chains of the aniline and indole moieties since the change in the para position of the phenyl top moiety hugely reduced the potency of the compound.^25^ In this procedure, the SwissBioisostere database (http://www.swissbioisostere.ch)^31^ was utilized to replace current moieties within the reference ligands, thereby maintaining or increasing the intended biological activity against DENV NS4B. The database focused explicitly on identifying atomic replacements or functional groupings, utilizing data obtained by mining bioactivity information from the ChEMBL database and detecting matched molecular pairs. The creation of analogues included exploring alternative groups or scaffolds to optimize compounds for greater activity, improved pharmacokinetics (ADME), or reduced toxicity.^31^ Four in-house compounds, BNJ-278, BNJ-390, BNJ-391, and BNJ-495, were also included in the study for comparison.

### Molecular Screening

The bioisostere data set was then screened for ADMET properties using ADMETlab 3.0. Lipinski rule was used to screen the compounds that might show good oral absorption and permeability.^32^ Compounds that passed Lipinski rule, were characterized by four essential parameters: molecular weight (MW) not exceeding 500 Da, a log P value of 5 or less, a maximum of 10 hydrogen-bond acceptors (HBAs), and a maximum of 5 hydrogen-bond donors (HBDs). Compounds that met at least 3 criteria were indicated with a score of 0. The Pfizer 3/75 rule is predicated on two requirements: a topological polar surface area (TPSA) of less than 75 and a log P value larger than 3.^33,34^ Compounds that met Pfizer’s criteria were scored 0 and indicated to have a high probability of nontoxicity. On the other hand, compounds that did not meet either of these requirements were assigned a score of 1, indicating that these compounds might have a high probability of toxicity.^33^ Compounds satisfying the selected Lipinski and Pfizer 3/75 criteria were prioritized for subsequent 3D-QSAR prediction, whereas compounds failing these filters were deprioritized. With the 3D-QSAR model, we utilized the model to predict the activity of our compounds against each serotype of the NS4B protein, followed by compounds being ranked according to their predicted activity.

## Experimental Evaluation by the IPK Assay

### Materials and Reagents

Dengue virus serotype 2 (DENV-2; strain BR DEN2 01-01, GenBank accession no. JX073928) was used in all experiments. Huh7.5 cells were obtained as previously described.^35^ The antiviral compounds remdesivir (MCE, HY-104077) and ribavirin (MP Biomedicals, 219606650) were used as reference controls. Viral infection was detected using the antiflavivirus envelope protein monoclonal antibody 4G2, produced in the D1-4G2-4-15 hybridoma cell line (ATCC, HB-112).

### IPK Assay Procedure

The predictions from the 3D-QSAR were validated using the IPK assays. Huh7.5 cells were seeded into 384-well plates 1 day prior to infection. Twenty-point dose–response curves were generated for each test compound. Compounds (0.25 μL per well) were transferred into cell-seeded plates using a HummingWell liquid handling system. Cells were subsequently infected with DENV-2 (BR DEN2 01-01: GenBank JX073928) at a multiplicity of infection (MOI) of 2. At 48 h of post-infection (hpi), cells were fixed with paraformaldehyde (PFA).

Viral 4G2 envelope protein and host cell nuclei were stained by immunofluorescence to assess cell number and viability. Images were acquired using an Operetta CLS high-content imaging system (PerkinElmer) and quantitatively analyzed with Columbus software (PerkinElmer) to determine inhibition of viral infection and cell viability. Half-maximal inhibitory concentration (IC_50_) values were calculated by fitting the data to sigmoidal dose–response curves using XLfit.

## Results and Discussion

### Molecular Docking and Alignment

#### Molecular Docking

The sequence similarity between the four NS4B serotypes ranges between 76 and 82% (Figure 1B). High conservation is observed in the TM regions, while variability is observed in the extracellular loops. The 3D crystal structure of NS4B is not currently available in the Protein Data Bank probably due to its dynamic, highly hydrophobic, and membrane-associated nature. Therefore, the structure of the NS4B protein was generated by using AlphaFold 3 (Figure 1C/D).^36^ The AlphaFold 3 model reported the accuracy of the entire structure by using the predicted template modeling (pTM) score. The pTM scores of serotypes 1, 2, 3, and 4 NS4B models were 0.79, 0.79, 0.78, and 0.81, respectively. The models were considered to be reliable based on the pTM score >0.5 indicating that the overall predicted fold for the complex might be similar to the true structure.^37^ Another score function used by AlphaFold is pLDDT (predicted Local Distance Difference Test).^38^ It is a per-residue score that indicates the model’s confidence in the predicted local environment of each amino acid, which focuses on the local distances between atoms.^37^ The overall structure of the models was presented at a high confidence level, as the average pLDDT of the entire structure was 80 (Figures 1D/S4). However, the loop regions were indicated to be of low confidence due to their dynamic nature. Lastly, the predicted aligned error (PAE) matrix represents the estimated inaccuracy in the relative orientation and location of two tokens within the expected structure. Greater values signify a greater projected error and, hence, a lower level of confidence.^36^ The overall structures in all serotypes were indicated to have moderate PAE (Figure S4). Ramachandran plots were used to evaluate the stereochemical quality of backbone dihedral angles (Phi (φ) and Psi (ψ)). In all serotypes of NS4B models, most residues fell in the favorable (red), additional allowed (yellow), and generously allowed regions. None of the residues fell in outlier (white) regions, which denote appropriate stereochemical geometry. Individual residue conformations are shown as black dots (Figure S4).

To experimentally validate interactions between JNJ-1802 and the DENV NS4B protein, Goethals et al. expressed a C-terminally hemagglutinin (HA)-affinity-tagged NS4A-2K-NS4B precursor in its wild-type (WT) form or bearing the V91A, L94F, or T108I substitutions.^28^ Previous mutagenesis studies identified in DENV-2 reported that the substitutions V91A, L94F, and T108I reduce sensitivity to JNJ-1802 and impair the disruption of the NS3–NS4B interaction.^28^ These findings suggest that the residues contribute to the inhibitor-binding region and were therefore used to guide the identification of the putative binding pocket. Based on this observation and to further elucidate the structural basis of ligand recognition, we docked JNJ-1802 into the predicted binding pocket of DENV-2 NS4B. Docking analysis revealed that the compound occupies a deep, cup-shaped cavity formed by the surrounding transmembrane helices of NS4B (Figure 2). The binding site is composed of 27 amino acid residues: W38, T39, Y41, A42, V43, T45, T46, T49, P50, D88, G90, V91, L93, L94, A95, E165, K166, Q170, H177, P209, T216, S220, G234, F237, S238, K241, and N242 (Figure 2).

**Figure 2:**
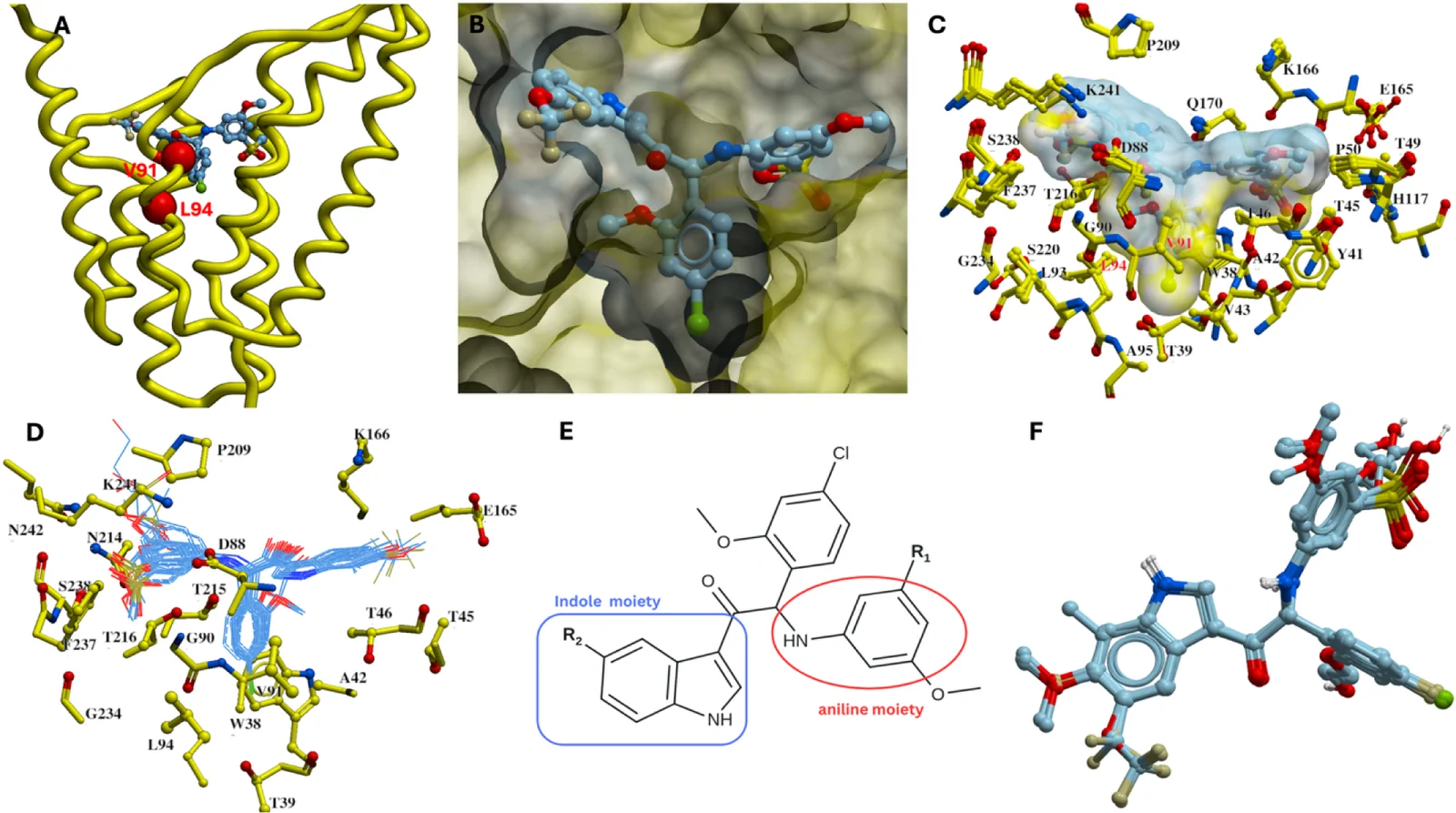
(A) The position of the DENV-2 NS4B putative ligand-binding site between the transmembrane helices. The positions of V91 and L94 are highlighted as red spheres. (B) Enlarged side view of the binding site. The protein is represented as a molecular surface. (C) The putative binding pocket is formed from 27 residues. The ligand is represented as a surface. Residues from all four DENV serotypes have been superimposed and illustrated. The positions of V91 and L94 are annotated in red. (D) Binding poses of all JNJ-series compounds docked in DENV-2 NS4B. (E) Chemical structure of the reference ligand derived from JNJ-1802, highlighting the key bioisosteric regions. The indole moiety is outlined in blue, and the aniline moiety is circled in red. (F) Structural alignment of all JNJ ligands.

Detailed inspection of ligand–protein interactions highlighted multiple scaffold-specific contacts. Within the phenyl moiety, the chlorine substituent engaged in hydrophobic interactions with A42, V43, V91, and L94, while the aromatic phenyl core participated in stabilizing π–alkyl interactions with A42, V91, and L94. In the aniline region, the sulfone functionality formed weak carbon–hydrogen interactions with residues T45 and T46. On the indole side of the molecule, the trifluoromethoxy group established a halogen bond with D88, a hydrogen bond with N242, and additional hydrophobic contacts with S238 and K241. Furthermore, the NH group of the pyrrole ring contributed a hydrogen bond to T216 (Figure 2).

It is important to note that previous structure–activity relationship studies of the JNJ series have highlighted the importance of the meta-methoxy substituent for antiviral potency. In the selected docking pose, this group is positioned near the entrance of the binding pocket, where it may contribute through steric and hydrophobic complementarity. The methyl sulfone moiety extends toward a more polar region of the cavity and may remain partially solvent-accessible. Alternative ligand orientations cannot be excluded in the absence of experimentally determined structural information. Therefore, the proposed binding mode should be regarded as a structure-informed working hypothesis that is consistent with the currently available mutagenesis and SAR data.

The predicted binding pose of JNJ-1802 is consistent with published mutagenesis studies that implicate residues V91 and L94 in inhibitor susceptibility and resistance. Comparative sequence (Figure 1B) and structural analysis (Figure 2) further demonstrate that this binding pocket is highly conserved across all four DENV serotypes, with near-complete residue conservation. The sole exception is position 237, which is occupied by leucine rather than phenylalanine in DENV-3, suggesting a broadly conserved binding mode for JNJ-1802 across the serotypes.

#### Molecular Alignment

To identify a common and reliable binding mode, all inhibitors in the data set were docked simultaneously into the generated NS4B structure. The resulting poses were analyzed to determine the most frequently occurring and best-predicted binding orientation. This consensus pose, which aligned well with the reference ligand JNJ-1802 and showed interactions with key residues V91 and L94, was selected and used as the reference for the consistent alignment of all generated compounds (Figure 2).

Data set alignment is a critical factor in developing 3D-QSAR models with high predictive accuracy.^29^ Molecular docking methods can align and generate suitable inhibitor conformations, resulting in 3D-QSAR models with statistical quality comparable to that of manual alignment. Furthermore, docking-based alignment models enhance the design of new inhibitors by elucidating interactions between inhibitors and amino acids within the active site.^39^

#### 3D-QSAR Model

The JNJ data set was divided into a training set of 15 ligands and a test set of 6 ligands. A field-based approach was used to construct the 3D-QSAR model by correlating interaction energy fields with the biological activities of aligned compounds.^40^ The Field-Based QSAR method employs the Comparative Molecular Similarity Indices Analysis (CoMSIA) method, utilizing specific parameters.^41^ For CoMSIA-like models, the field style is Gaussian (Schrödinger, Inc., 2022). CoMSIA evaluates five fields with distinct physicochemical characteristics (steric, electrostatic, hydrophobic, and hydrogen-bonding donor and acceptor) using a Gaussian-type distance-dependent function, which avoids singularities at atomic positions.^42^ The experimental activity of JNJ compounds against dengue virus serotype 2 (DENV-2) NS2B, measured as pEC_50_ (the negative logarithm of the EC_50_), was determined using both enhanced green fluorescent protein (eGFP)-infected cells and reverse transcription polymerase chain reaction (RT-PCR) assays.^25^ Experimental activity data from RT-PCR were selected for model construction because this genotypic antiviral assay was applied to all serotypes of DENV NS4B, allowing for a comparison of predicted activity across protein types and the identification of the most effective antiviral compounds. After constructing the aligned data set and building the 3D-QSAR model, partial least-squares (PLS) regression analysis was used to refine the model, with a maximum of three PLS factors considered. Specifically, the extended Gaussian-based potential was selected as the molecular field type, resulting in three models that incorporated hydrophobic, steric, electrostatic, hydrogen-bond acceptor (HBA), hydrogen-bond donor (HBD), and aromatic-ring fields. For each factor, the predicted activity values of the data set were calculated. The optimal model was selected based on statistical robustness (Table 1).^43^

**Table 1:** Statistical Model for Field-Based 3D QSAR of JNJ Ligands in Each Type of DENV NS4B Complex

Type of DENV	SD	R^2^	R^2^_CV_	R^2^_scramble_	Stability	F	P	RMSE	Q^2^	Pearson-r
1	0.2606	0.9188	0.3963	0.7394	0.585	41.5	2.74 × 10^–6^	0.25	0.8927	0.9822
2	0.2343	0.9138	0.6436	0.6148	0.837	38.9	3.79 × 10^–6^	0.23	0.8905	0.9528
3	0.242	0.9043	0.6625	0.5444	0.839	34.6	6.74 × 10^–6^	0.26	0.8811	0.9537
4	0.1301	0.9678	0.3357	0.7144	0.431	110.3	1.72 × 10^–8^	0.16	0.9228	0.9653

For the DENV-2 NS4B protein, the best model was determined to have a PLS factor of 3. The coefficient of determination, or R^2^, is 0.9138, which indicates that 91% of the variation in the observed activity can be explained by the model. The cross-validation R^2^ was calculated using an N-out strategy, yielding an R^2^_CV_ of 0.6436. The R^2^_scramble_, which calculates the average value achieved utilizing scrambled activities, came out to be 0.6148 (Table 1). Several models constructed with scrambled activities were used to calculate this parameter. Higher values indicate that the variable set may be reasonably complete and that the model can accommodate a wide range of data, but lower values suggest that the model cannot fit random data.^44^

The statistic for the model prediction of the changes gained in the composition of the training set was found to be 0.837. The model-to-actual activity variance ratio is provided by the descriptor F. Since the activity variance is spread across n – m – 1 (n ligands, m PLS factors), and the model variance is computed for the dispersed data over m degrees of freedom, the obtained value of 38.9 is the result. Regression is more significant when the F value is large.^43^ The Pearson (P) descriptor measured 3.79 × 10^–6^, where the P descriptor is treated as a ratio of Chi-squared distributions. A smaller P value indicates a greater degree of confidence.^43^ With an RMSE of 0.23, a Q^2^ of 0.8905, and a Pearson r of 0.9528, the external validation metrics show that the model is good and capable of predicting the antiviral activity of BNJ compounds and bioisosteres that were not part of the model creation process (Table 1).^30^

The scatter plots shown display the correlation between experimental and predicted pEC_50_ values for both the training set and the test set (Figure 3). Thus, all presented data indicate good stability and high statistical significance of the field-based 3D-QSAR model. A limitation of the present study is the relatively small size and restricted chemical diversity of the training set. This reflects the availability of experimentally characterized compounds within the JNJ inhibitor series. Therefore, the developed model is expected to be the most reliable within the chemical space represented by this scaffold series and should not be extrapolated to highly dissimilar chemotypes without further validation.

**Figure 3:**
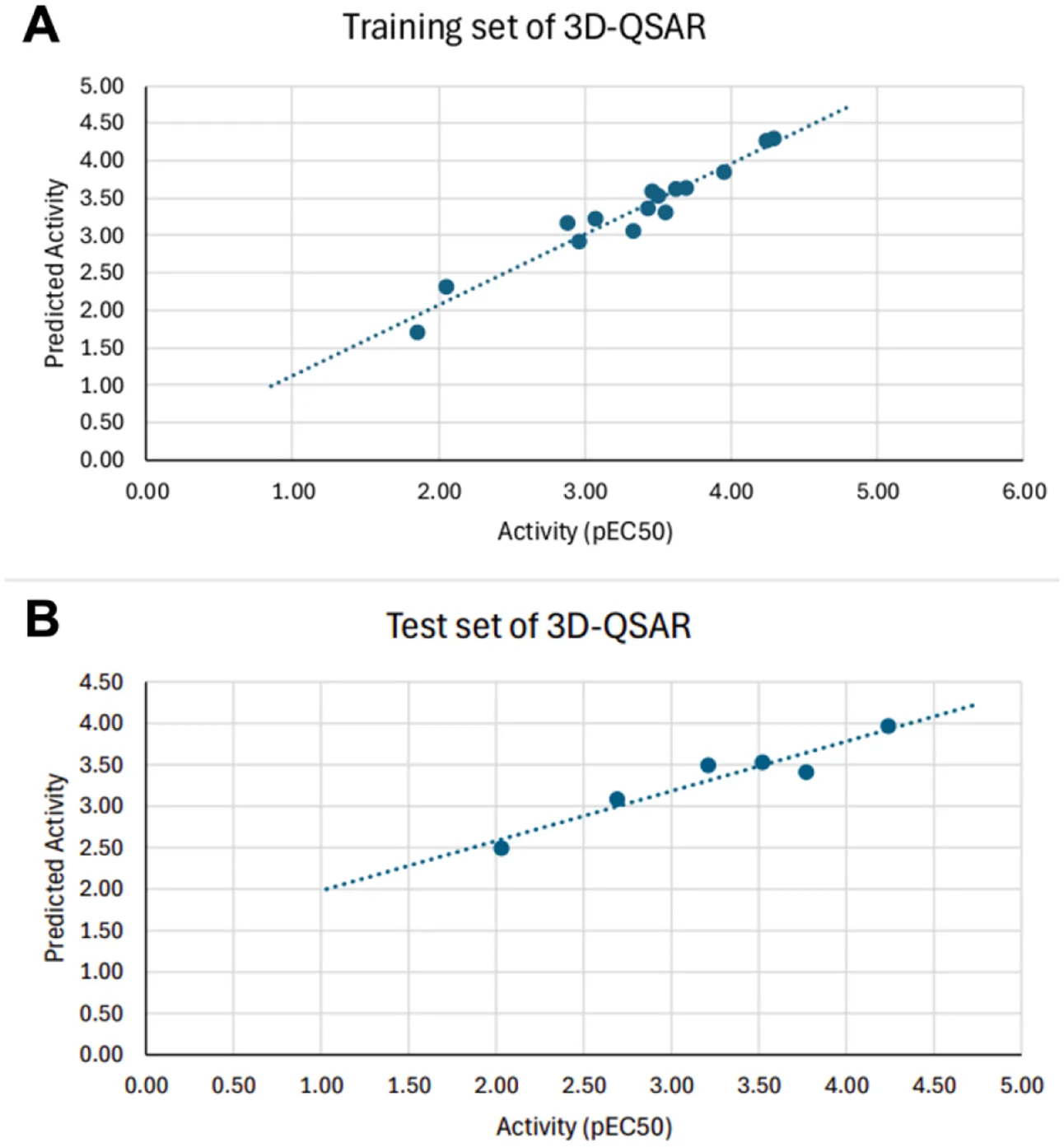
Correlation between the experimental and predicted biological activities for the DENV-2 complex. (A) Training set of 3D-QSAR highlights the correlation between the experimental activity and the activities predicted by the model for the compounds used in the training phase. (B) Test set of 3D-QSAR illustrates the external validation of the model using a separate test set of compounds to ensure its predictive reliability for novel structures.

### Bioisostere Generation

To explore alternative molecular designs and identify ligands with improved predicted activities, bioisosteric analogues of JNJ-1802 were generated. The two key functional moieties of JNJ-1802, the indole and aniline cores (Figure 2), were submitted to both SwissBioisosteres and ICM (MolSoft ICM 3.9-1b) to identify suitable bioisosteric replacements. ICM suggested 347 replaceable side chains on the aniline core and 27 replaceable groups on the indole core. SwissBioisosteres generated approximately 500 replacement options across both of the cores. The proposed substitutions varied considerably in terms of their structural compatibility with the JNJ scaffold. The 374 bioisosteric replacements generated by ICM showed substantial overlap with the replacement suggestions obtained from the SwissBioisostere Database. In this context, consensus refers to bioisosteric transformations that were independently identified by both of the approaches. Several replacement options suggested by SwissBioisostere were excluded because they introduced unfavorable physicochemical features, excessive molecular complexity, or alterations considered incompatible with the established JNJ scaffold. Consequently, 374 consensus analogues were selected for further analysis (Supporting Information Sheet 1). The observed differences in predicted activity between the compounds implied that binding interactions were the dominant determinants of potency, and therefore, physicochemical properties were not considered in the development of the QSAR model.

### Molecular Screening

In the first screening for ADMET properties, only 69 compounds passed both the Lipinski’s rule and the Pfizer 3/75 rule.^33^ After finishing the first screening, the prediction activity of both JNJ-1802’s analogue ligands was estimated by using the prediction function implemented in Schrödinger Maestro. The software can predict the biological activity of new, uncharacterized ligands by inserting them into a preexisting 3D grid and determining their field values. The predicted activity of all ligands is listed in Supporting Information 2. With a combination of both ADMET properties and predicted activity, there were 2 compounds that showed promising potential. In BIS-222 (Table 2), which has a substituted 3-hydroxypyrrolidin-2-one on an aniline core, the highest potential activity is observed against serotype 2 NS4B, with an estimated pEC_50_ of 4.277 (Figure 4). Among in-house ligands, BNJ-495, a substituted phosphinate group on an aniline core was predicted to have antiviral activity with a pEC_50_ of 4.230. However, when every serotype NS4B was considered, BIS-222 dominated on both serotype 1, with a predicted activity of 3.684, and serotype 2. Nevertheless, its potency significantly decreased against serotypes 3 and 4, with pEC_50_ of 1.88 and 1.538, respectively. On the other hand, the predicted activities of BNJ-495 against DENV-1, DENV-2, DENV-3, and DENV-4 were 3.511, 4.230, 2.596, and 1.895, respectively. Like BIS-222, BIS-47 and BIS-161 exhibited marked serotype-dependent variation in predicted pEC_50_ values. Although BIS-47 showed the highest predicted activity against DENV-3 and BIS-161 against DENV-4, both compounds displayed substantially reduced predicted activity against the remaining serotypes. Specifically, BIS-47 pEC_50_ significantly dropped against types 2 and 4 NS4B, and BIS-161 predicted activity was reduced against serotypes 1, 2, and 3 (Supporting Information 2). Based on this prediction, BNJ-495 was identified as the most effective pan-serotype inhibitor of NS4B.

**Figure 4:**
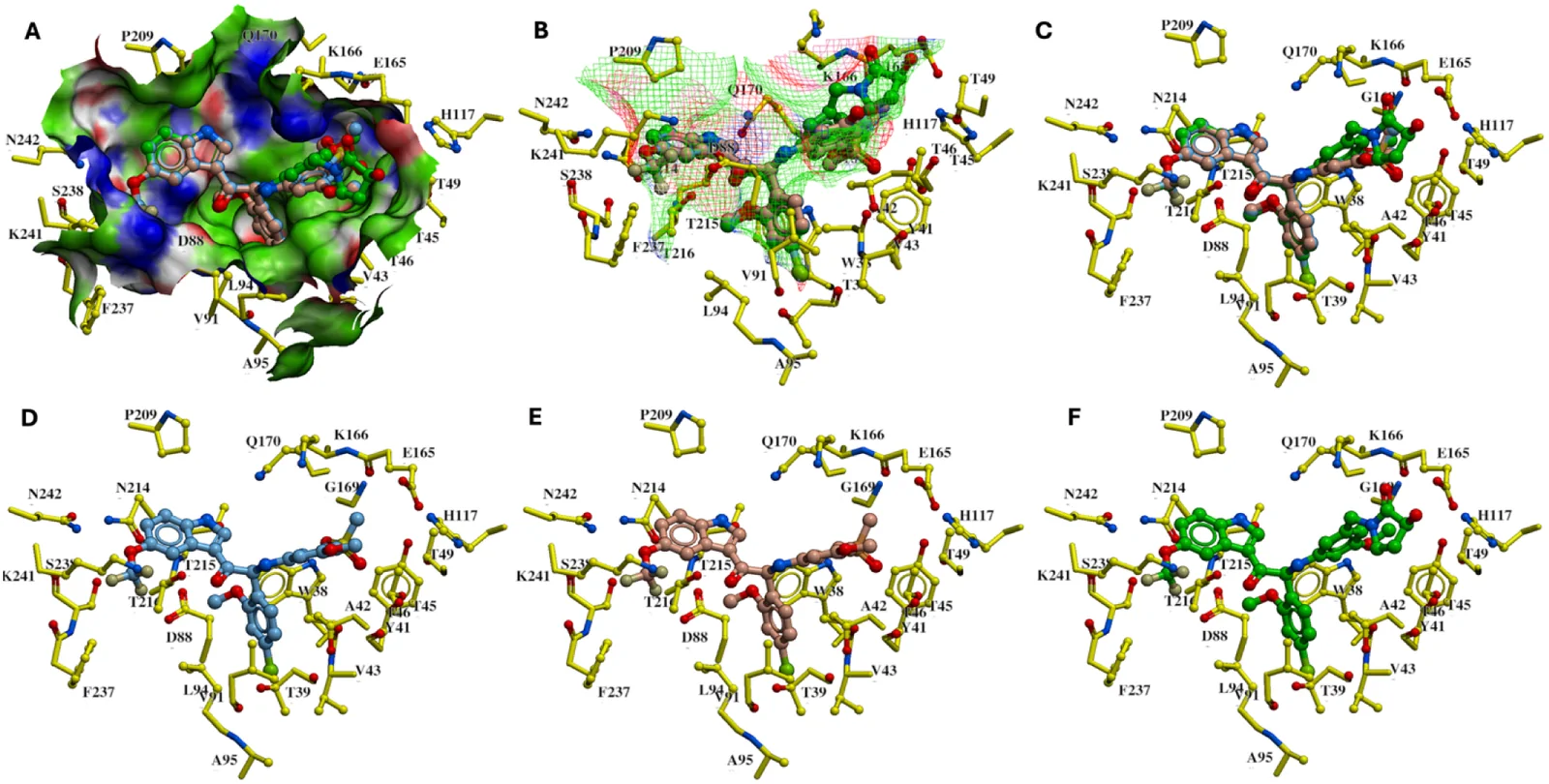
Molecular docking of ligands. (A) Topological view of JNJ-1802 (cyan), BNJ-495 (pink), and BIS-222 (green) binding to the NS4B serotype-2 binding pocket. The protein surface is rendered with electrostatic potential mapping (blue: positive, red: negative, green: hydrophobic). The overlapping ligands and the surrounding residues (yellow) are shown in sticks. (B) Mesh representation of the binding pocket volume, illustrating spatial occupancy and steric fit of the ligands relative to the surrounding residue side chains (yellow). (C) Composite overlay of JNJ-1802 (cyan), BNJ-495 (pink), and BIS-222 (green) highlighting conserved docking orientations and key residue interactions. Individual binding poses for (D) JNJ-1802, (E) BNJ-495, and (F) BIS-222.

**Table 2:**
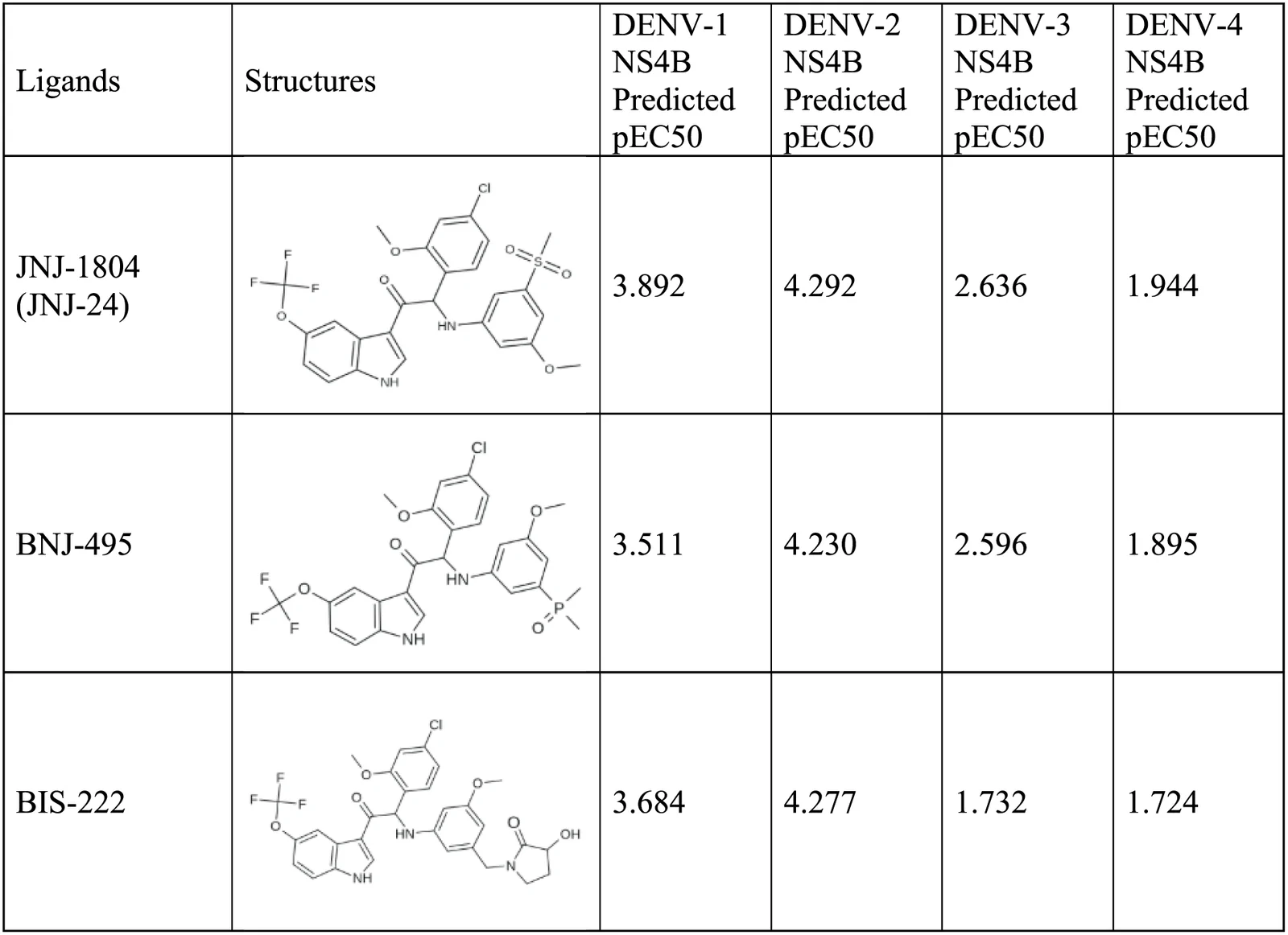
Predicted Activity of JNJ-1802 Analogues (BIS) and In-House Ligands (BNJ) against All Four Serotypes of DENV NS4B

### Experimental Validation

Using the IPK immunofluorescence-based viral assay system, BNJ compounds were evaluated for antiviral activity against dengue virus serotype 2 in duplicate experiments. Remdesivir was included on each assay plate as a positive control to ensure assay quality and to assess IC_50_ reproducibility and antiviral activity. Four BNJ compounds were supplied in liquid form at a concentration of 10 mM (100 μL each). Compounds were tested in 20-point, 2-fold serial dose–response curves starting at 50 μM, with all concentrations assessed in duplicate.

Among the four in-house BNJ compounds tested, BNJ-495 demonstrated the most pronounced antiviral activity against dengue virus serotype 2 in the IPK immunofluorescence assay. BNJ-495 exhibited a very high level of potency, with an IC_50_ of 0.00017 μM indicating strong inhibition of viral infection at low nanomolar concentrations (Table 3). The dose–response curve exhibited a steep Hill slope (4.33), consistent with a sharp and robust antiviral effect over a narrow concentration range (Figure 5). Cytotoxicity analysis showed a CC_50_ of 6.11 μM, resulting in a very high selectivity index (SI) of approximately 37,000. These results showed a clear separation between antiviral efficacy and host cell toxicity under the assay conditions.

**Table 3:** Summary of IC_50_ and CC_50_ values of the tested 4 in-house compounds.

	Inhibition of Infection (%)			Cell ratio (%)			
Compound Name	Hill (slope)	IC_50_(μM)	IC(Y = 50)	Min (%)	CC_50_ (μM)	CC (Y = 50)	SI
**BNJ–278**	3.08	0.023	0.022	0.34	5.12	5.23	240
**BNJ–390**	2.76	0.0011	0.001	34.61	^a^PI	19.06	18279
**BNJ–391**	2.37	0.0052	0.0051	78.12	^a^PI	>50	9838
**BNJ–495**	4.33	0.00017	0.00017	0.17	6.11	6.41	37146

a PI = Partial Inhibition

**Figure 5:**
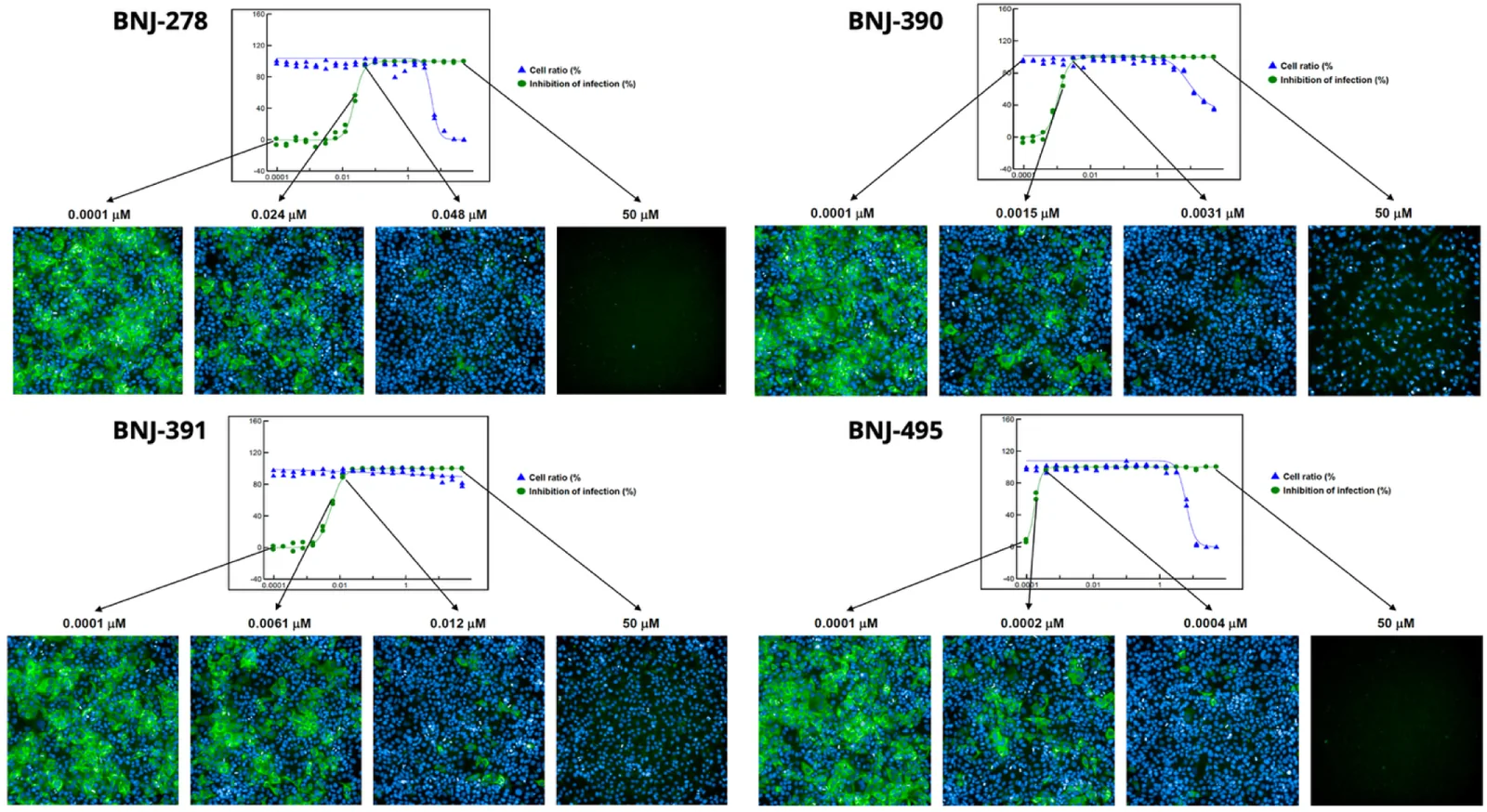
Effect of BNJ inhibitors on Dengue virus serotype-2 infection assay. The antiviral activity and cellular toxicity of four selected compounds (BNJ-278, BNJ-390, BNJ-391, and BNJ-495) were evaluated across a broad concentration range. The dose–response curves illustrate the % of viral inhibition (green circles) and cell ratio (blue triangles) reflecting host-cell viability. Potent dose-dependent inhibition of infection was observed for all compounds, with near-complete suppression of viral replication at higher concentrations. The corresponding cell ratio data indicate the compounds maintain a favorable safety profile at lower effective doses, though significant reductions in cell viability were noted at the highest tested concentrations for BNJ-278 and BNJ-495. The immunofluorescence images confirm the quantitative data (cell nuclei are visualized in blue, while viral proteins are labeled in green). A marked, concentration-dependent reduction in the green fluorescence signal is evident across the three representative concentrations shown for each compound (0.0001 μM to 0.048 μM), demonstrating the effective neutralization of the virus in a cellular environment.

The remaining compounds also showed antiviral activity but with lower potency compared to BNJ-495. BNJ-390 and BNJ-391 exhibited low-nanomolar IC_50_ values (0.0011 μM and 0.0052 μM, respectively) and did not reach 50% cytotoxicity within the concentration range tested, suggesting acceptable cell tolerance (Table 3). BNJ-278 displayed the weakest antiviral activity among the tested compounds, with an IC_50_ of 0.023 μM and a CC_50_ of 5.12 μM.

The ranking predicted by the QSAR model was generally consistent with the experimental antiviral activity. BNJ-495 was predicted to be the most promising broadly active compound among the in-house series and subsequently demonstrated the highest antiviral potency in the DENV-2 assay. These findings provide preliminary support for the predictive utility of the QSAR within this chemical series. Also, worth noting is that the BNJ compounds evaluated experimentally were synthesized and tested as racemic mixtures. The reported antiviral activities, therefore, represent the combined contribution of both enantiomers. While the high potency of BNJ-495 demonstrates that at least one stereoisomer possesses favorable antiviral activity, the relative contribution of the individual enantiomers remains unknown. Future studies employing enantiomerically pure compounds will be necessary to determine stereochemical preferences and further refine the proposed NS4B-binding model.

## Conclusions

In conclusion, we aimed to discover new ligands or analogue compounds that are comparable to JNJ-1802 in antiviral activity by finding the possible binding pose and constructing a QSAR model. Without a high-resolution structure, four serotypes of the NS4B protein model were built by using AlphaFold 3, and the quality of the models was reported with high confidence scores. The JNJ ligand data set was docked to all serotypes to identify the binding conformation of the ligands. The conformations of the ligands were aligned together to find the consensus pose and interactions that were satisfied with previously published mutagenesis data. To identify the most potent compounds, we constructed a data set of bioisosteres of JNJ-1802 (BIS-1 to BIS-374) and in-house compounds (BNJ-278, BNJ-390, BNJ-391, and BNJ-495). The data set was screened with ADMET properties to increase the chance of success in development. The structure–activity relationship (3D-QSAR) allowed us to predict the potency or activity of our analogues. The results of predicted activity from 3D-QSAR indicate that BNJ-495 is the most potent ligand among the four in-house ligands against all four serotypes of the NS4B protein. For analogues, BIS-222 is the most potent against DENV-1 and DENV-2 NS4B, BIS-47 is the most potent against DENV-3 NS4B, and BIS-161 is the most potent against DENV-4 NS4. Importantly, experimental validation confirmed the activity predicted by the 3D-QSAR model, which reinforced the validity of the computational approach. Although computational analyses predicted activity across all four dengue serotypes, antiviral testing was performed only against DENV-2, and further experimental validation is required to confirm BNJ-495’s pan-serotype activity. The present workflow employing integrated modeling, QSAR, and experimental results highlights BNJ-495 as a promising NS4B-targeting antiviral candidate and demonstrates the utility of rationalized structure-based methods in guiding dengue antiviral discovery.

## Supplementary Material







## Data Availability

Information about all molecules is detailed in the manuscript and Supporting Information.

## Notes

Supplementary Figures S1–S4 (library of JNJ compounds, selected bioisosteres, in-house BNJ compounds, quality assessment of NS4B models, scheme of compounds); synthetic procedures and quality analysis of compounds and intermediates (PDF)
Supplementary Data 1: Bioisosteres (XLSX)
Supplementary Data 2: List of all compounds (XLSX)

## Abbreviations

QSAR: quantitative structure–activity relationship
DENV: Dengue virus; RNA—ribonucleic acid
DNA: deoxyribonucleic acid
JNJ: Johnson and Johnson
TPSA: topological polar surface area
HBA: hydrogen bond acceptor
HBD: hydrogen bond donor
MW: molecular weight
ADMET: Absorption Distribution Metabolism Excretion Toxicity
PFA: paraformaldehyde
pTM: predicted template modeling
pLDDT: predicted local distance difference test
pAE: predicted aligned error
RT-PCR: reverse transcription-polymerase chain reaction

## References

[1] Wilder-Smith A., Hombach J., Ferguson N., Selgelid M., O’brien K., Vannice K., Barrett A., Ferdinand E., Flasche S., Guzman M. (2019). Deliberations
of the
Strategic Advisory Group of Experts on Immunization on the Use of
CYD-TDV Dengue Vaccine. Lancet Infectious Diseases.

[2] Calisher C. H., Karabatsos N., Dalrymple J. M., Shope R. E., Porterfield J. S., Westaway E. G., Brandt W. E. (1989). Antigenic Relationships between Flaviviruses
as Determined by Cross-Neutralization Tests with Polyclonal Antisera. J. Gen. Virol..

[3] Guzman M. G., Halstead S. B., Artsob H., Buchy P., Farrar J., Gubler D. J., Hunsperger E., Kroeger A., Margolis H. S., Martínez E., Nathan M. B., Pelegrino J. L., Simmons C., Yoksan S., Peeling R. W. (2010). Dengue: A Continuing
Global Threat. Nat. Rev. Microbiol..

[4] San
Martín J. L., Brathwaite O., Zambrano B., Solórzano J. O., Bouckenooghe A., Dayan G. H., Guzmán M. G. (2010). The Epidemiology
of Dengue in the Americas Over the Last Three Decades: A Worrisome
Reality. Am. Soc. Tro. Med. Hyg..

[5] World Health Organization. WHO Report on Global Surveillance of Epidemic-Prone Infectious Diseases; 2000, 127, https://iris.who.int/handle/10665/66485.

[6] Velayudhan, R. Global Strategy For Dengue Prevention And Control, 2012–2020; World Health Organization, 2012,49

[7] Wang W.-H., Urbina A. N., Chang M. R., Assavalapsakul W., Lu P.-L., Chen Y.-H., Wang S.-F. (2020). Dengue
Hemorrhagic
Fever – A Systemic Literature Review of Current Perspectives
on Pathogenesis, Prevention and Control. J.
Microbiol., Immunol. Infect..

[8] Rodenhuis-Zybert I. A., Wilschut J., Smit J. M. (2010). Dengue
Virus Life Cycle: Viral and
Host Factors Modulating Infectivity. Cell. Mol.
Life Sci..

[9] Yang Z.-S., Baua A. D., Hemdan M. S., Assavalapsakul W., Wang W.-H., Lin C.-Y., Chao D.-Y., Chen Y.-H., Wang S.-F. (2025). Dengue Virus Infection: A Systematic Review of Pathogenesis,
Diagnosis and Management. J. Infect. Public
Health.

[10] Li Q., Kang C. (2022). Dengue Virus
NS4B Protein as a Target for Developing Antivirals. Front. Cell. Infect. Microbiol..

[11] Li Q., Kang C. (2022). Structures and Dynamics of Dengue Virus Nonstructural
Membrane Proteins. Membranes.

[12] Chatel-Chaix L., Fischl W., Scaturro P., Cortese M., Kallis S., Bartenschlager M., Fischer B., Bartenschlager R. (2015). A Combined
Genetic-Proteomic Approach Identifies Residues within Dengue Virus
NS4B Critical for Interaction with NS3 and Viral Replication. J. Virol..

[13] Umareddy I., Chao A., Sampath A., Gu F., Vasudevan S. G. (2006). Dengue
Virus NS4B Interacts with NS3 and Dissociates It from Single-Stranded
RNA. J. Gen. Virol..

[14] Kundharapu S., Chowdary T. K. (2022). Dengue Virus NS4b
N-Terminus Disordered Region Interacts
with NS3 Helicase C-Terminal Subdomain to Enhance Helicase Activity. Viruses.

[15] Lu H., Zhan Y., Li X., Bai X., Yuan F., Ma L., Wang X., Xie M., Wu W., Chen Z. (2021). Novel Insights
into the Function of an N-Terminal Region of DENV2 NS4B for the Optimal
Helicase Activity of NS3. Virus Res..

[16] Kiemel D., Kroell A.-S. H., Denolly S., Haselmann U., Bonfanti J.-F., Andres J. I., Ghosh B., Geluykens P., Kaptein S. J. F., Wilken L., Scaturro P., Neyts J., Van Loock M., Goethals O., Bartenschlager R. (2024). Pan-Serotype
Dengue Virus Inhibitor JNJ-A07 Targets NS4A-2K-NS4B Interaction with
NS2B/NS3 and Blocks Replication Organelle Formation. Nat. Commun..

[17] Morrison J., Aguirre S., Fernandez-Sesma A. (2012). Innate Immunity
Evasion by Dengue
Virus. Viruses.

[18] Khanra M., Ghosh I., Khatun S., Ghosh N., Gayen S. (2025). Dengue Virus-Host
Interactions: Structural and Mechanistic Insights for Future Therapeutic
Strategies. J. Struct. Biol..

[19] Dengue Vaccine: WHO – Recommendations sition Paper September. Vaccine 2019, 37 (35), 4848–4849. 10.1016/j.vaccine.2018.09.063 30424888

[20] Thomas S. J., Yoon I.-K. (2019). A Review of Dengvaxia®:
Development to Deployment. Hum. Vaccines Immunother..

[21] Wang Q.-Y., Dong H., Zou B., Karuna R., Wan K. F., Zou J., Susila A., Yip A., Shan C., Yeo K. L., Xu H., Ding M., Chan W. L., Gu F., Seah P. G., Liu W., Lakshminarayana S. B., Kang C., Lescar J., Blasco F., Smith P. W., Shi P.-Y. (2015). Discovery of Dengue
Virus NS4B Inhibitors. J. Virol..

[22] Chauhan N., Gaur K. K., Asuru T. R., Guchhait P. (2024). Dengue Virus: Pathogenesis
and Potential for Small Molecule Inhibitors. Biosci. Rep..

[23] Wang Y., Xie X., Shi P.-Y. (2022). Flavivirus NS4B Protein: Structure, Function, and Antiviral
Discovery. Antiviral Res..

[24] Wang Y., Sun L., Fernandes L., Wang Y.-H., Zou J., Franklin S. J., Hu Y., Palmer L. K., Yeung J., Barriga D., Russell W. K., Moquin S. A., Shi P.-Y., Skepper C., Xie X. (2025). Mechanistic
Insights into Dengue Virus Inhibition by a Clinical Trial Compound
NITD-688. Proc. Natl. Acad. Sci. U. S. A..

[25] Kesteleyn B., Bonfanti J.-F., Bardiot D., De Boeck B., Goethals O., Kaptein S. J. F., Stoops B., Coesemans E., Fortin J., Muller P., Doublet F., Carlens G., Koukni M., Smets W., Raboisson P., Chaltin P., Simmen K., Loock M. V., Neyts J., Marchand A., Jonckers T. H. M. (2024). Discovery of JNJ-1802, a First-in-Class
Pan-Serotype Dengue Virus NS4B Inhibitor. J.
Med. Chem..

[26] Kaptein S. J. F., Goethals O., Kiemel D., Marchand A., Kesteleyn B., Bonfanti J.-F., Bardiot D., Stoops B., Jonckers T. H. M., Dallmeier K., Geluykens P., Thys K., Crabbe M., Chatel-Chaix L., Münster M., Querat G., Touret F., De Lamballerie X., Raboisson P., Simmen K., Chaltin P., Bartenschlager R., Van Loock M., Neyts J. (2021). A Pan-Serotype Dengue
Virus Inhibitor Targeting the NS3–NS4B Interaction. Nature.

[27] Abagyan R., Totrov M., Kuznetsov D. (1994). ICM—A
New Method for Protein
Modeling and Design: Applications to Docking and Structure Prediction
from the Distorted Native Conformation. J. Comput.
Chem..

[28] Goethals O., Kaptein S. J. F., Kesteleyn B., Bonfanti J.-F., Van Wesenbeeck L., Bardiot D., Verschoor E. J., Verstrepen B. E., Fagrouch Z., Putnak J. R., Kiemel D., Ackaert O., Straetemans R., Lachau-Durand S., Geluykens P., Crabbe M., Thys K., Stoops B., Lenz O., Tambuyzer L., De Meyer S., Dallmeier K., McCracken M. K., Gromowski G. D., Rutvisuttinunt W., Jarman R. G., Karasavvas N., Touret F., Querat G., De Lamballerie X., Chatel-Chaix L., Milligan G. N., Beasley D. W. C., Bourne N., Barrett A. D. T., Marchand A., Jonckers T. H. M., Raboisson P., Simmen K., Chaltin P., Bartenschlager R., Bogers W. M., Neyts J., Van Loock M. (2023). Blocking NS3–NS4B
Interaction Inhibits Dengue Virus in Non-Human Primates. Nature.

[29] Jiang X., Kopp-Schneider A. (2014). Summarizing
EC50 Estimates from Multiple Dose-response
Experiments: A Comparison of a Meta-analysis Strategy to a Mixed-effects
Model Approach. Biom. J..

[30] Ozgencil F., Eren G. (2023). Field-Based 3D-QSAR
Studies on Amide- and Urea-Containing NAMPT Inhibitors. Chemom. Intell. Lab. Syst..

[31] Wirth M., Zoete V., Michielin O., Sauer W. H. B. (2013). SwissBioisostere:
A Database of Molecular Replacements for Ligand Design. Nucleic Acids Res..

[32] Lipinski C. A., Lombardo F., Dominy B. W., Feeney P. J. (2001). Experimental and
Computational Approaches to Estimate Solubility and Permeability in
Drug Discovery and Development Settings 1PII of Original Article:
S0169–409X(96)­00423–1. Adv. Drug
Delivery Rev..

[33] Hughes J. D., Blagg J., Price D. A., Bailey S., DeCrescenzo G. A., Devraj R. V., Ellsworth E., Fobian Y. M., Gibbs M. E., Gilles R. W., Greene N., Huang E., Krieger-Burke T., Loesel J., Wager T., Whiteley L., Zhang Y. (2008). Physiochemical
Drug Properties Associated with in Vivo Toxicological Outcomes. Bioorg. Med. Chem. Lett..

[34] Yukawa T., Naven R. (2020). Utility of Physicochemical
Properties for the Prediction of Toxicological
Outcomes: Takeda Perspective. ACS Med. Chem.
Lett..

[35] Blight K. J., McKeating J. A., Rice C. M. (2002). Highly Permissive Cell Lines for
Subgenomic and Genomic Hepatitis C Virus RNA Replication. J. Virol..

[36] Abramson J., Adler J., Dunger J., Evans R., Green T., Pritzel A., Ronneberger O., Willmore L., Ballard A. J., Bambrick J., Bodenstein S. W., Evans D. A., Hung C.-C., O’neill M., Reiman D., Tunyasuvunakool K., Wu Z., Žemgulytė A., Arvaniti E., Beattie C., Bertolli O., Bridgland A., Cherepanov A., Congreve M., Cowen-Rivers A. I., Cowie A., Figurnov M., Fuchs F. B., Gladman H., Jain R., Khan Y. A., Low C. M. R., Perlin K., Potapenko A., Savy P., Singh S., Stecula A., Thillaisundaram A., Tong C., Yakneen S., Zhong E. D., Zielinski M., Žídek A., Bapst V., Kohli P., Jaderberg M., Hassabis D., Jumper J. M. (2024). Accurate Structure
Prediction of
Biomolecular Interactions with AlphaFold 3. Nature.

[37] Xu J., Zhang Y. (2010). How Significant Is a Protein Structure Similarity with
TM-Score =
0.5?. Bioinformatics.

[38] Jumper J., Evans R., Pritzel A., Green T., Figurnov M., Ronneberger O., Tunyasuvunakool K., Bates R., Žídek A., Potapenko A., Bridgland A., Meyer C., Kohl S. A. A., Ballard A. J., Cowie A., Romera-Paredes B., Nikolov S., Jain R., Adler J., Back T., Petersen S., Reiman D., Clancy E., Zielinski M., Steinegger M., Pacholska M., Berghammer T., Bodenstein S., Silver D., Vinyals O., Senior A. W., Kavukcuoglu K., Kohli P., Hassabis D. (2021). Highly Accurate Protein
Structure Prediction with AlphaFold. Nature.

[39] Ul-Haq Z., Mahmood U., Reza S. (2012). A Combined 3D-QSAR and Molecular
Docking Strategy to Understand the Binding Mechanism of V600EB-RAF
Inhibitors. Mol. Divers..

[40] Cramer R. D., Patterson D. E., Bunce J. D. (1988). Comparative Molecular Field Analysis
(CoMFA). 1. Effect of Shape on Binding of Steroids to Carrier Proteins. J. Am. Chem. Soc..

[41] Karimi S., Shahabi F., Mubarak S. M. H., Arjmandi H., Hashemi Z. S., Pourzardosht N., Zakeri A., Mahboobi M., Jahangiri A., Rahbar M. R., Khalili S. (2022). Impact of SNPs, off-Targets, and
Passive Permeability on Efficacy of BCL6 Degrading Drugs Assigned
by Virtual Screening and 3D-QSAR Approach. Sci.
Rep..

[42] Da
Cunha E. F. F., Sippl W., De Castro Ramalho T., Ceva Antunes O. A., De Alencastro R. B., Albuquerque M. G. (2009). 3D-QSAR
CoMFA/CoMSIA Models Based on Theoretical Active Conformers of HOE/BAY-793
Analogs Derived from HIV-1 Protease Inhibitor Complexes. Eur. J. Med. Chem..

[43] Golbraikh A., Tropsha A. (2002). Predictive QSAR Modeling
Based on Diversity Sampling
of Experimental Datasets for the Training and Test Set Selection. J. Comput.-Aided Mol. Des..

[44] Ajjarapu S. M., Tiwari A., Taj G., Singh D. B., Singh S., Kumar S. (2021). Simulation Studies,
3D QSAR and Molecular Docking on a Point Mutation
of Protein Kinase B with Flavonoids Targeting Ovarian Cancer. BMC Pharmacol. Toxicol..

