# Supplementary material for: Structure-Guided Design of a Subnanomolar Dengue Virus NS4B Inhibitor
Source: J Chem Inf Model. 2026 Oct 6;66(20):13630–40. doi: 10.1021/acs.jcim.6c02098 (PMC13715512; doi:10.1021/acs.jcim.6c02098)

Supporting Information

Structure-guided design of a sub-nanomolar Dengue Virus NS4B inhibitor

Poomipat Laorsatiankul,[1,⊥] Mohammad Hassan Baig,[2,⊥] Shuang Chen,[1] Mehdi Hassan,[1,5] Patan Rasvan Khan,[2] Mala Ramanjaneyulu,[2] Kim Chang Joong,[2] Jo Yun Seong,[2] Jae-June Dong [2,3*], Shozeb Haider,[1,4*]

[1] Pharmaceutical and Biological Chemistry, UCL School of Pharmacy, London WC1N 1AX

[2] BNJ Biopharma, Yeonsu-gu, Incheon, 21983, Republic of Korea

[3] Department of Family Medicine, Kangbuk Samsung Hospital, Samsung Medical Center, Jongno District, Seoul, 03181, Republic of Korea

[4] Prince Fahad Bin Sultan Chair for Biomedical Research, University of Tabuk, Tabuk, 71491, Saudi Arabia

[5] Department of Chemistry University of Baltistan Skardu, Pakistan,16100

[⊥] Equal Contributions

**Correspondence:*

Dr. Jae-June Dong (jaejune.dong@samsung.com)

Dr Shozeb Haider (Shozeb.haider@ucl.ac.uk)

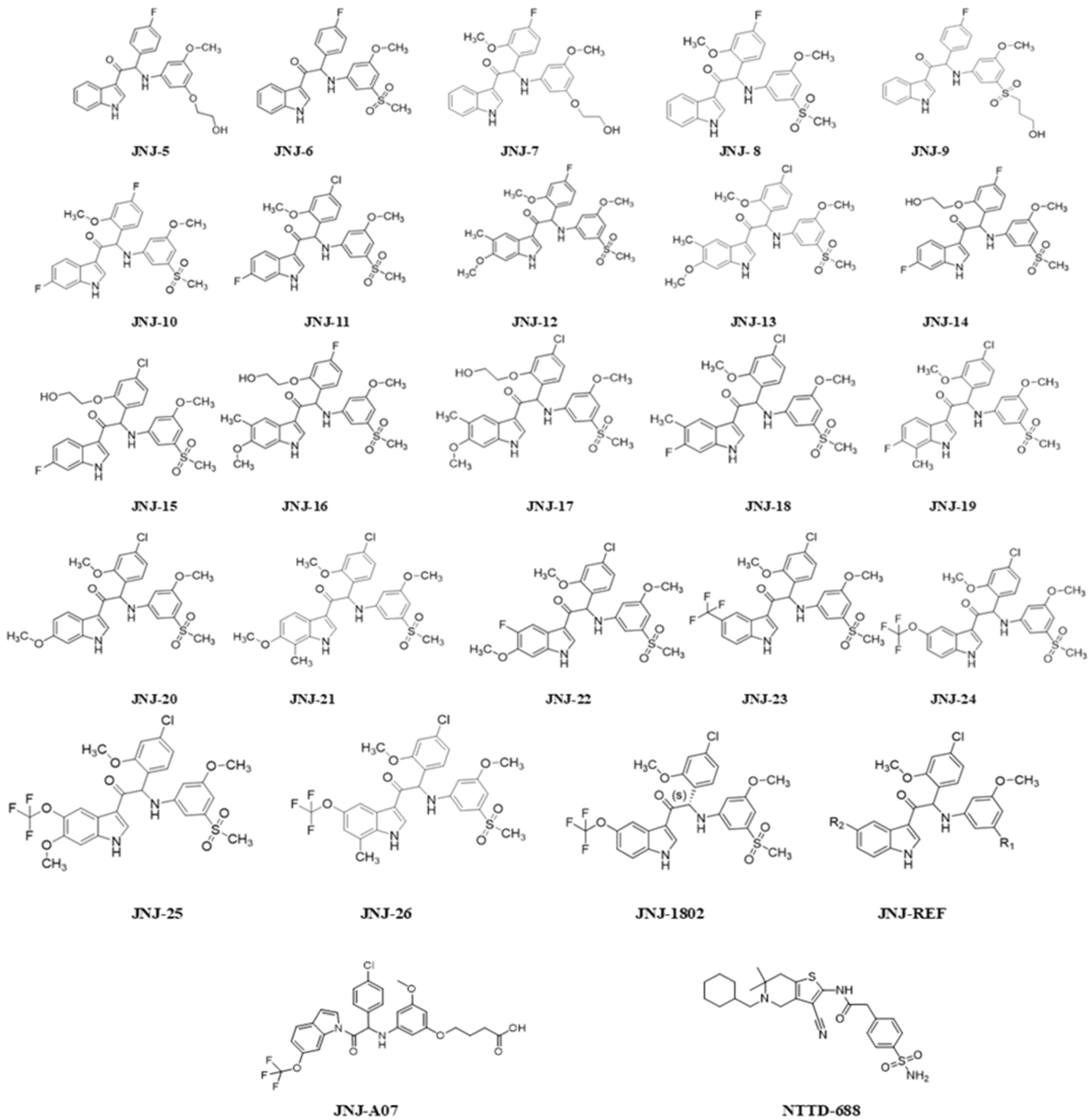

Figure S1. The library of JNJ compounds. Also included are reference molecules JNJ-REF, JNJ-A07, and the structurally distinct inhibitor NITD-688. JNJ-24 and JNJ-1802 are identical molecules.

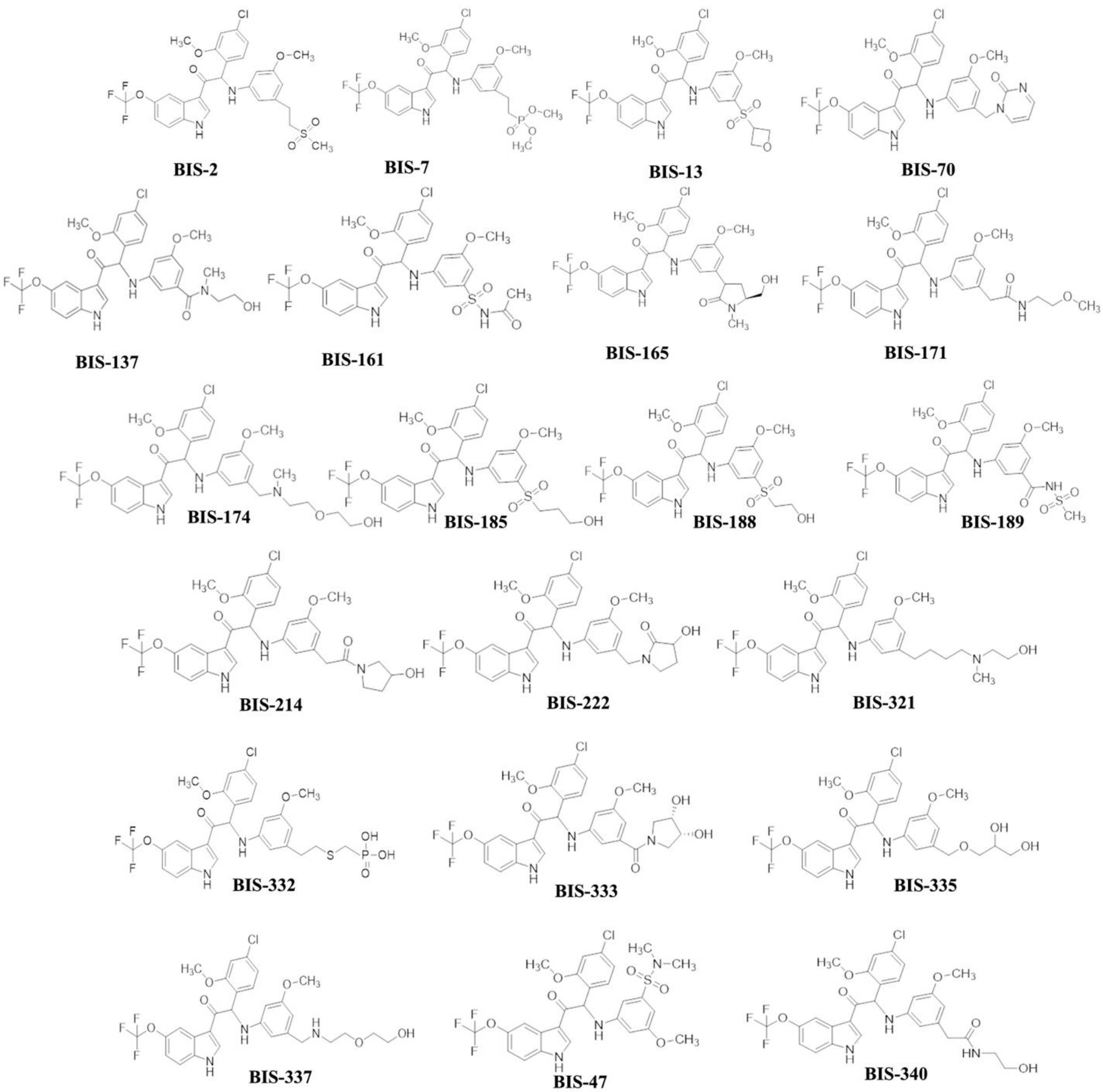

Figure S2. Structures of a selected BIS (bioisosteres) compounds used in this study. A detailed list of 374 compounds generated from the JNJ analogues are listed in Supporting Sheet 1.

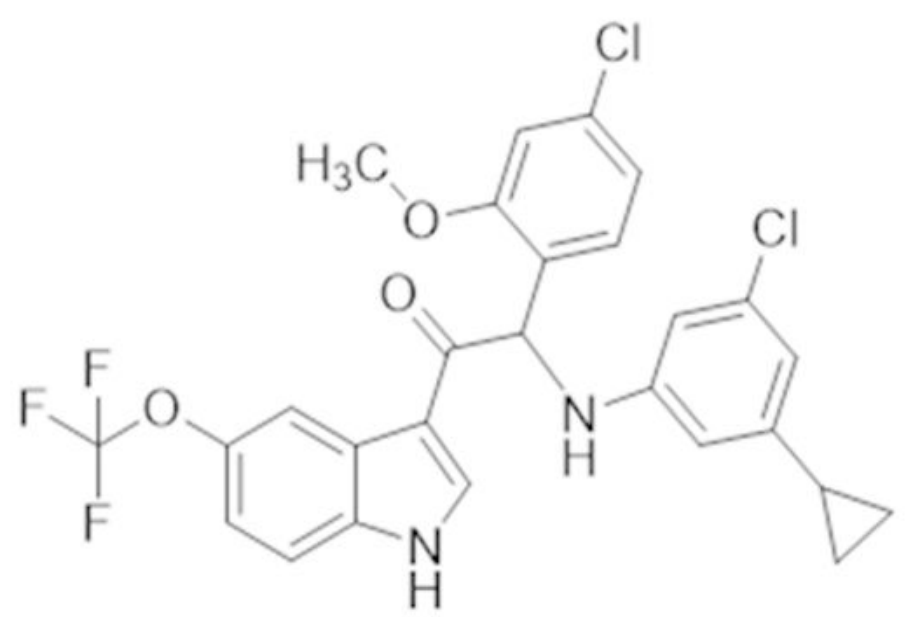

BNJ-278

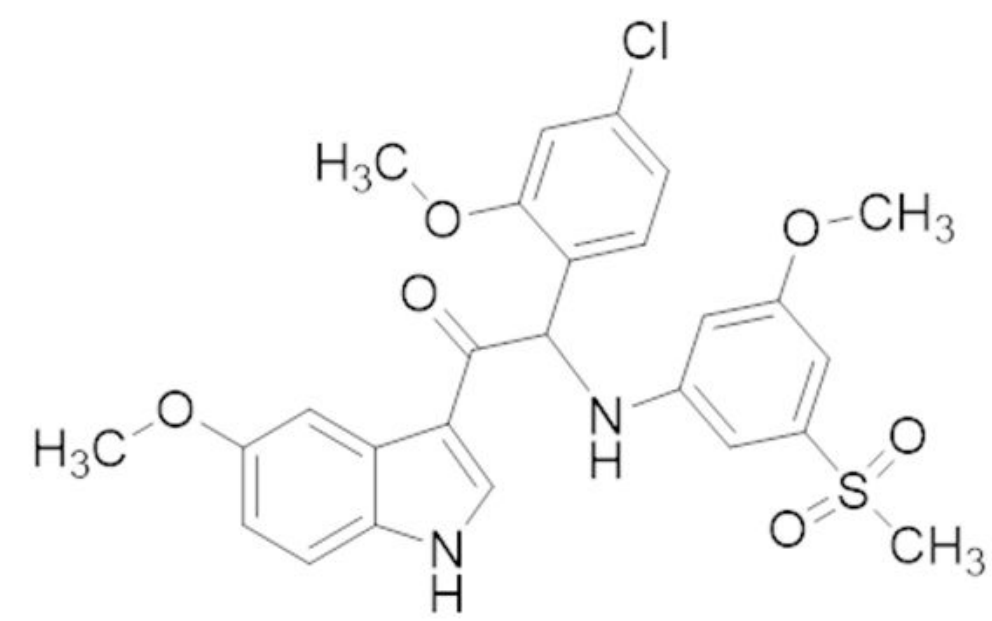

BNJ-390

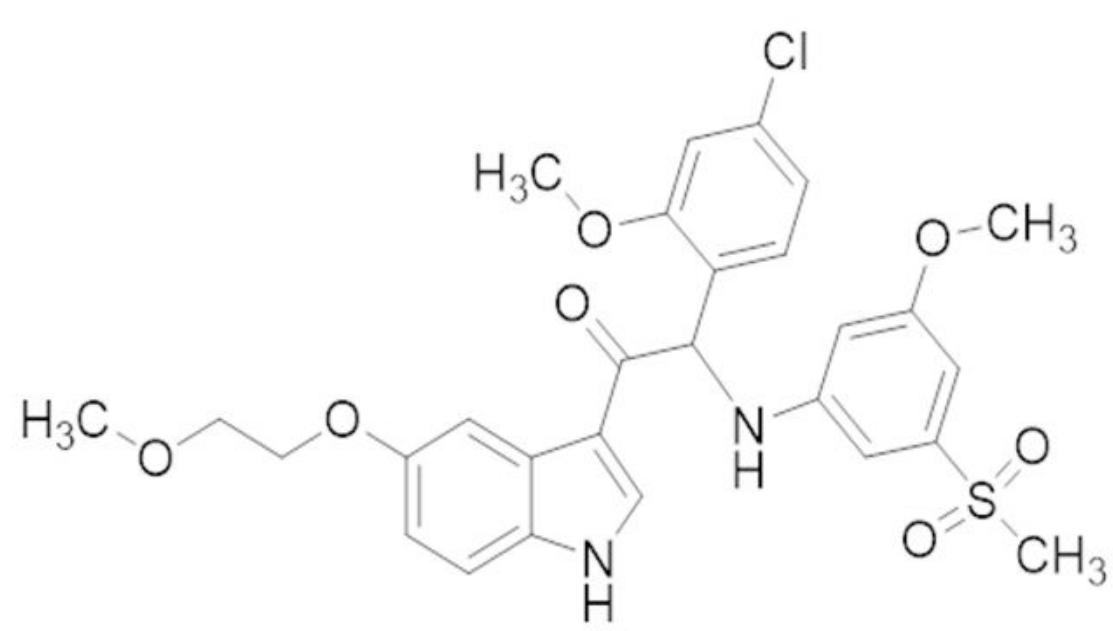

BNJ-391

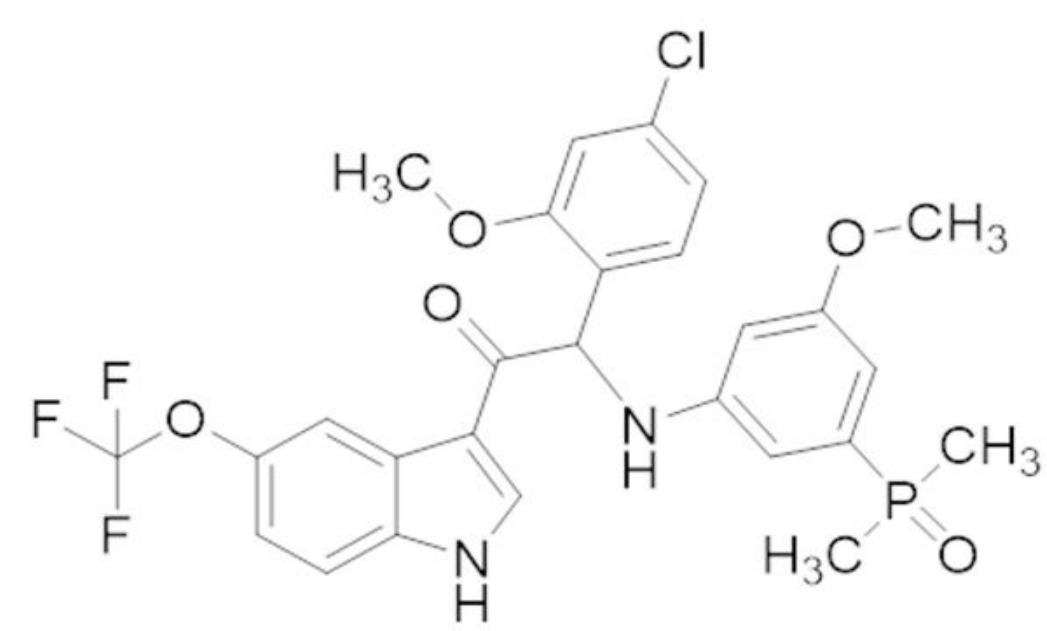

BNJ-495

Figure S3. In-house BNJ series compounds used in this study.

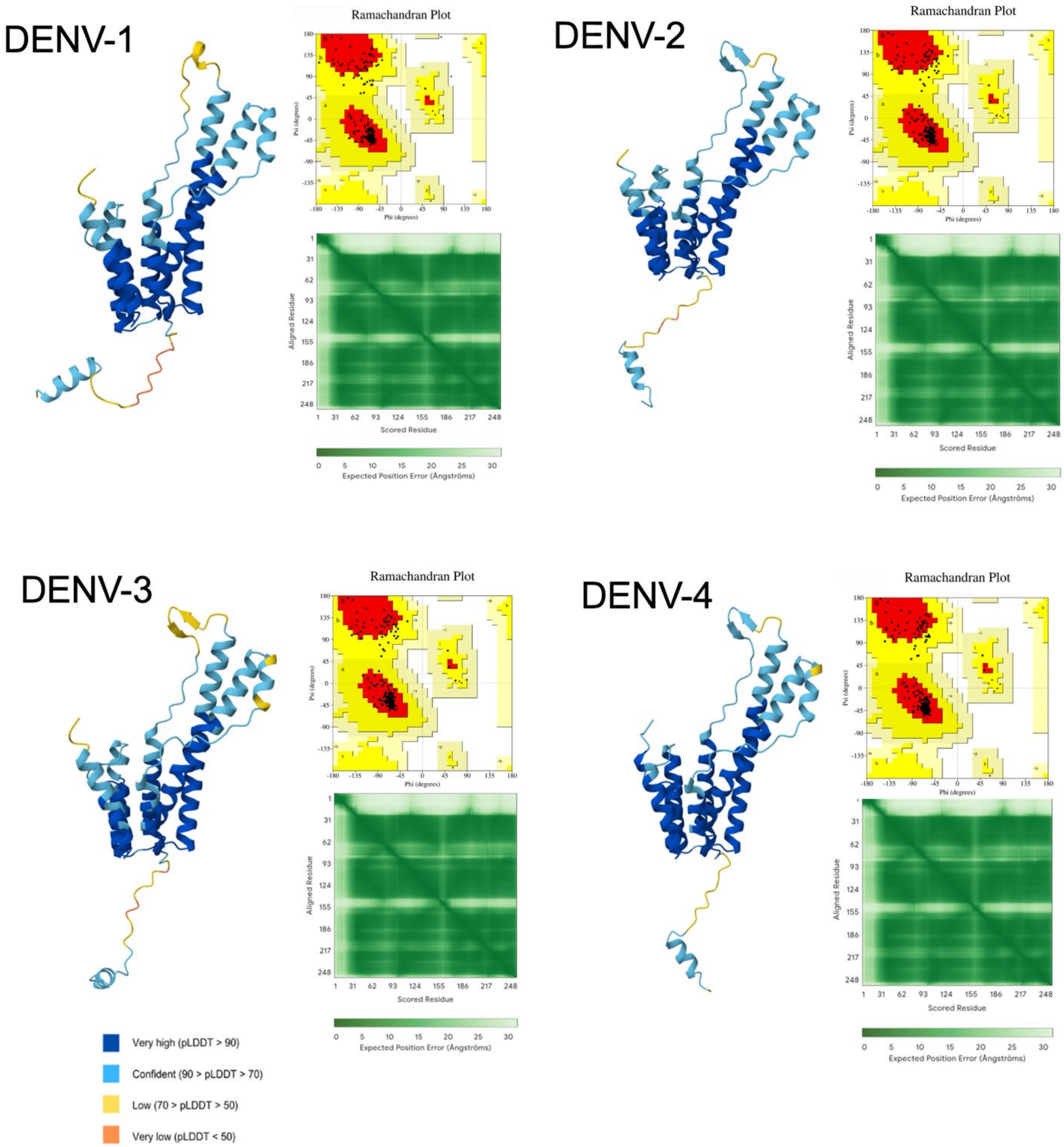

Figure S4. The assessment of the quality of the NS4B models. Model reliability is visualized through the per-residue confidence score (pLDDT), where blue indicates very high confidence (pLDDT > 90), light blue signifies good confidence, yellow denotes low confidence, and orange identifies unreliable regions. To further assess the spatial accuracy of the models, the Predicted Aligned Error (PAE) matrix was utilized to measure the expected inaccuracy in the relative distance (in Å) between residue pairs. Within this matrix, dark green represents a low PAE, indicating high confidence in the relative positions of residues, while light green indicates a higher PAE and greater positional uncertainty. The stereochemical quality of the protein backbone was validated using Ramachandran plots, which map the allowed and disallowed regions for the two primary backbone dihedral angles, ϕ and φ. These regions are categorized into favorable (red), additionally allowed (yellow), and generously allowed or outlier (white) regions.

Synthetic scheme of key intermediates:

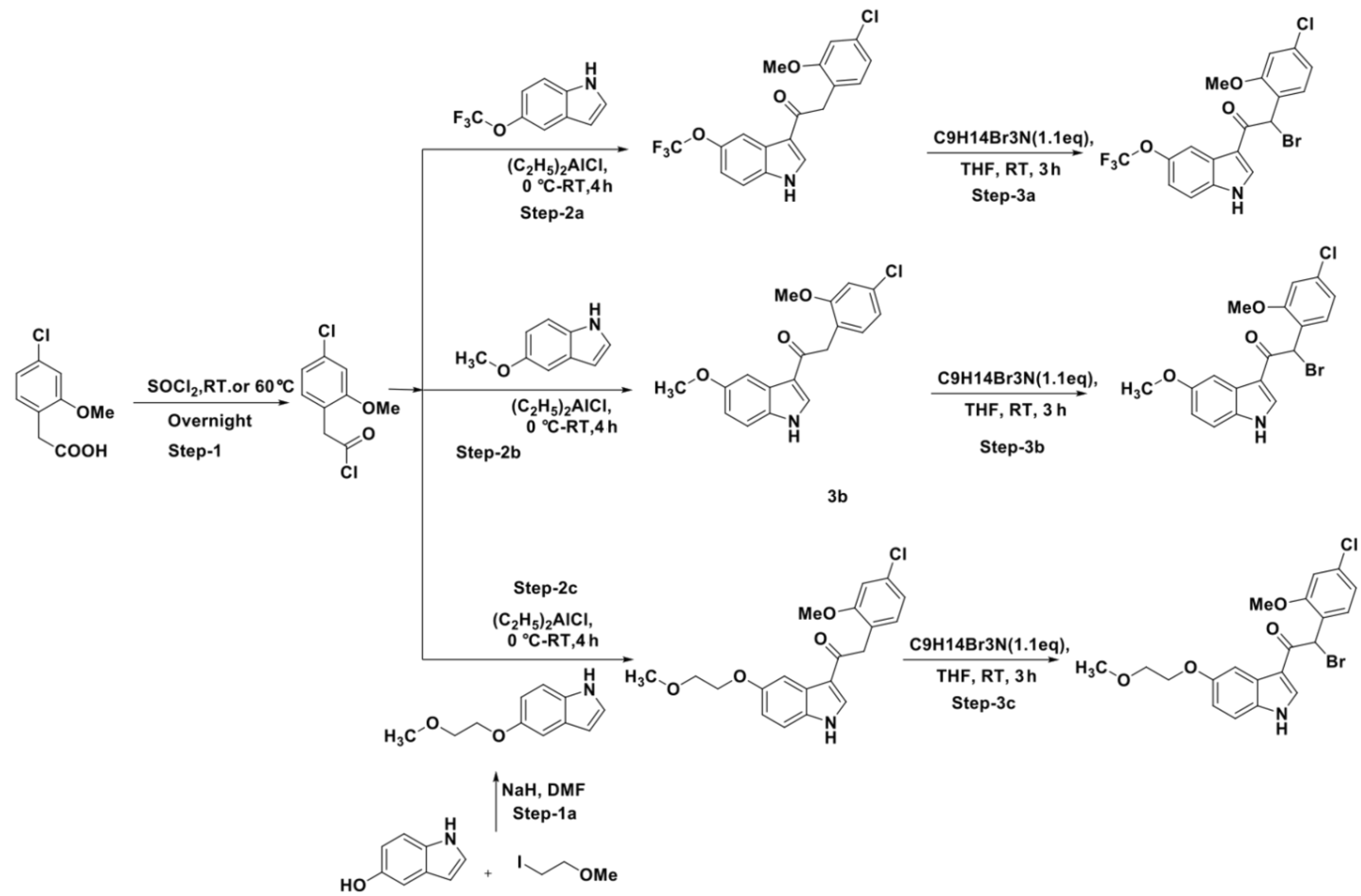

Synthetic scheme of BNJ-278:

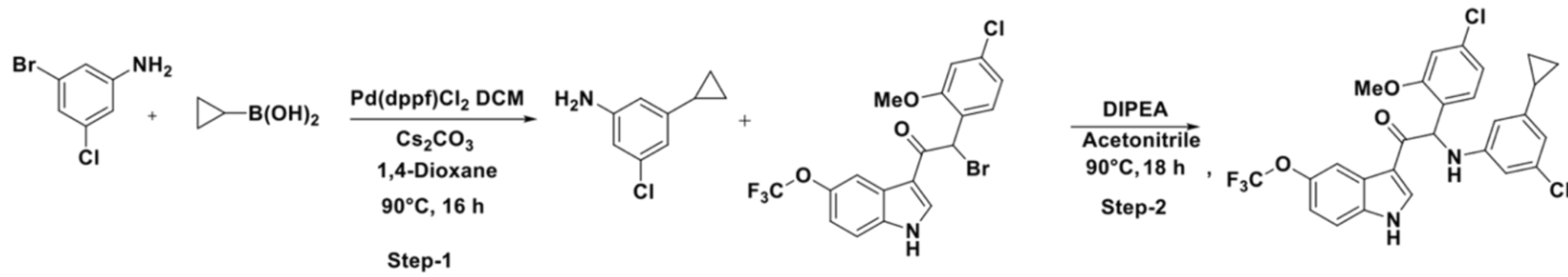

Synthetic scheme of BNJ-390:

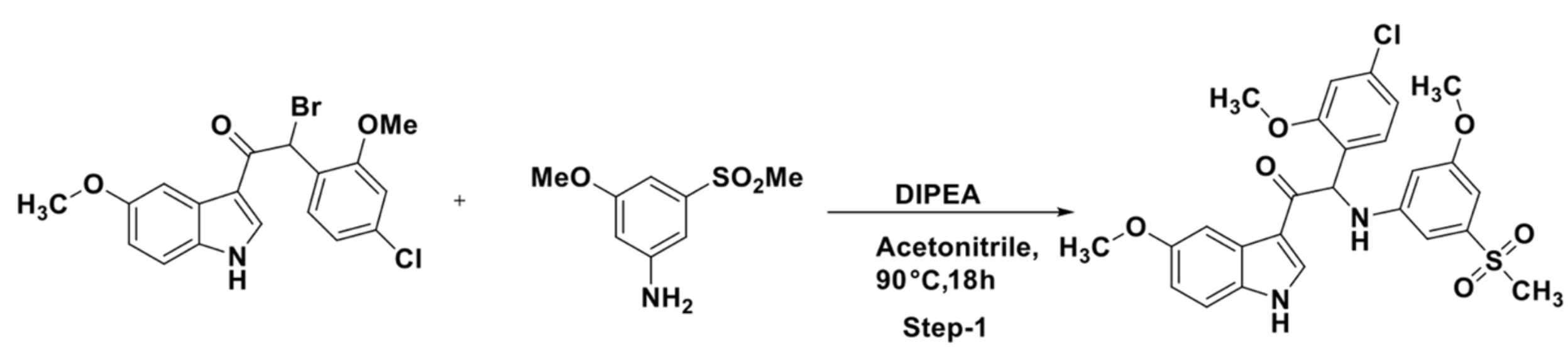

Synthetic scheme of BNJ-391:

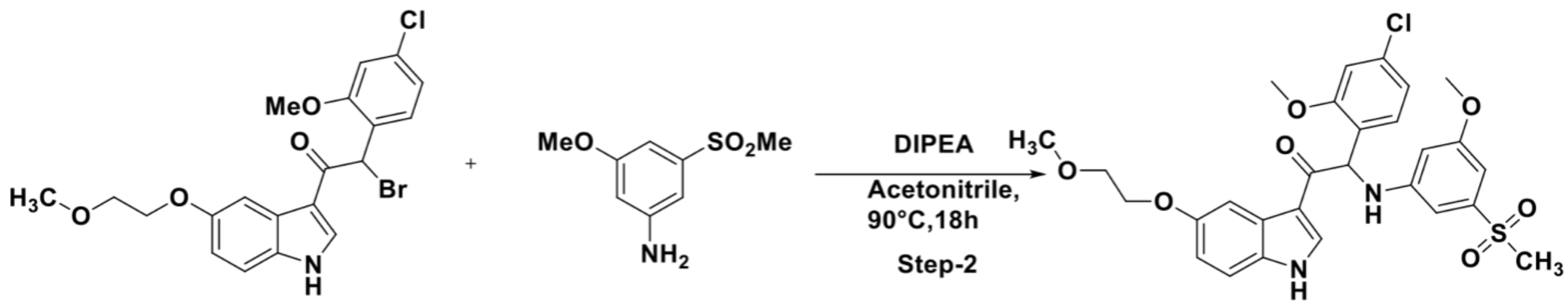

Synthetic scheme of BNJ-495:

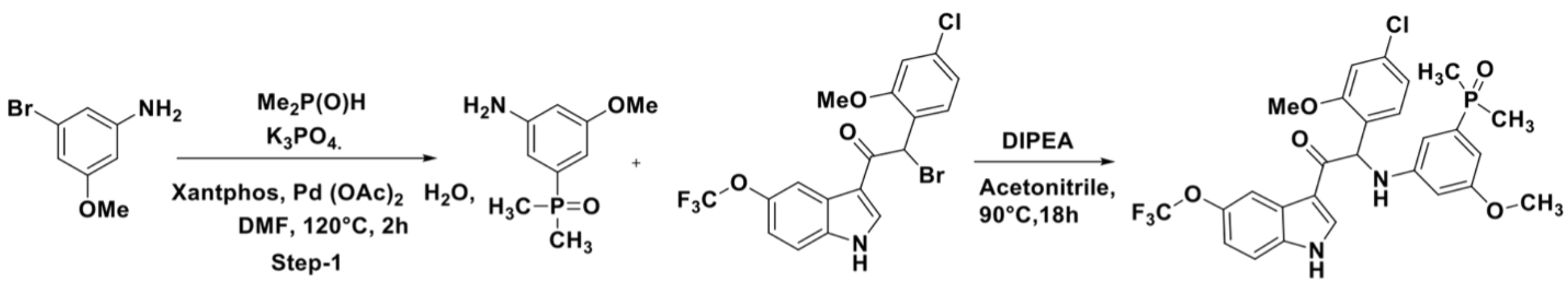

Synthetic procedures

1.Synthesis of *2-bromo-2-(4-chloro-2-methoxyphenyl)-1-(5-(trifluoromethoxy)-1H-indol-3-yl) ethan-1-one (*4a):

Step-1: Synthesis of *2-(4-chloro-2-methoxyphenyl) acetyl chloride* (2):

In a dry 100 mL round-bottom flask equipped with a magnetic stir bar and reflux condenser, 2-(4-chloro-2-methoxyphenyl) acetic acid (e.g., 5.0 g, 0.024 mol) was dissolved in 50 mL of dry THF under nitrogen atmosphere at 0°C was added Thionyl chloride (2.5 mL, ~0.030 mol) was added dropwise to the stirred solution while maintaining the temperature 0 °C using an ice bath. After complete addition, the reaction mixture was allowed to warm to room temperature and then heated to reflux (~70 °C) for 15 hours. The reaction progress was monitored by TLC (ethyl acetate: hexane, 1:4). Upon completion, the mixture was cooled to room temperature, and excess thionyl chloride and solvent were removed under reduced pressure using a rotary evaporator. The crude product was obtained as a pale-yellow oil and used directly in subsequent reactions without further purification. The product was stored under nitrogen in a sealed vial at 0–4 °C to prevent hydrolysis.

Step-2a: Synthesis of *2-(4-chloro-2-methoxyphenyl)-1-(5-(trifluoromethoxy)-1H-indol-3-yl) ethan-1-one (*3a):

A solution of 5-(trifluoromethoxy)-1H-indole (3g, 14.9 mmol) in DCM (150 mL) was cooled to 0 °C under a nitrogen atmosphere. A solution of diethyl aluminum chloride (1M in hexane, 22.4 mL, 22.4 mmol) was added dropwise, and the resulting mixture was kept at 0 °C for 15 min. A solution of 2-(4-chloro-2-methoxyphenyl) acetyl chloride, 4.57 g, 20.9 mmol) in DCM (100 mL) was added dropwise. Stirring was continued at 0 °C for 1 h, and the reaction mixture was subsequently stirred at room temperature for 4 h. The reaction mixture was poured out in a stirring ice/Rochelle salt solution. After the ice had melted, the mixture was filtered over dicalite and the filter cake was washed several times with tetrahydrofuran (THF). The filtrates were combined. The layers were separated, and the organic layer was washed with brine, dried over MgSO4, filtered, and evaporated under reduced pressure. The residue was tritrated with CH_2Cl_2 (50mL). The resulting precipitate was filtered and dried under vacuum at 50°C to provide 2-(4-chloro-2-methoxyphenyl)-1-(5-(trifluoromethoxy)-1H-indol-3-yl) ethan-1-one (4.3 g, 76%).

Step-3a: Synthesis of *2-bromo-2-(4-chloro-2-methoxyphenyl)-1-(5-(trifluoromethoxy)-1H-indol-3-yl) ethan-1-one (*4a*).* A stirred solution of 2-(4-chloro-2-methoxyphenyl)-1-(5-(trifluoromethoxy)-1H-indol-3-yl) ethan-1-one (4.39 g,11.4 mmol) in THF (200 mL) was cooled to 0°C. A solution of phenyl trimethyl ammonium tribromide ([CAS4207-56-1], 4.73 g, 12.6 mmol) in THF (100 mL) was added dropwise. The resulting suspension was stirred at room temperature for 2h. The solids were removed by filtration and washed with THF. The combined filtrates were evaporated under reduced pressure. The residue was mixed with EtOAc (30 mL). The solids were isolated by filtration, washed with a small amount of EtOAc, and dried under vacuum at 50 °C to provide 2-bromo-2-(4-chloro-2-methoxyphenyl)-1-(5-(trifluoromethoxy)-1H-indol-3-yl) ethan-1-one (5.0 g, yield: 94%) as a white solid, which was used in the next step without further purification.

2.Synthesis of *2-bromo-2-(4-chloro-2-methoxyphenyl)-1-(5-(trifluoromethoxy)-1H-indol-3-yl) ethan-1-one (*4b):

Step-2b: Synthesis of *2-(4-chloro-2-methoxyphenyl)-1-(5-methoxy-1H-indol-3-yl) ethan-1-one (*3b*)*:

A solution of 5-methoxy-1H-indole (3 g, 14.9 mmol) in DCM (150 mL) was cooled to 0 °C under a nitrogen atmosphere. A solution of diethyl aluminum chloride (1M in hexane, 22.4 mL, 22.4 mmol) was added dropwise, and the resulting mixture was kept at 0 °C for 15 min. A solution of 2-(4-chloro-2-methoxyphenyl) acetyl chloride, 4.57 g, 20.9 mmol) in DCM (100mL) was added dropwise. Stirring was continued at 0 °C for 1h, and the reaction mixture was subsequently stirred at room temperature for 4 h. The reaction mixture was poured out in a stirring ice/Rochelle salt solution. After the ice had melted, the mixture was filtered over dicalite and the filter cake was washed several times with tetrahydrofuran (THF). The filtrates were combined. The layers were separated, and the organic layer was washed with brine, dried over $MgSO_4$, filtered, and evaporated under reduced pressure. The residue was titrated with CH_2Cl_2 (50 mL). The resulting precipitate was filtered and dried under vacuum at 50 °C to provide 2-(4-chloro-2-methoxyphenyl)-1-(5-methoxy-1H-indol-3-yl) ethan-1-one (4.3 g, 76%).

Step-3b: Synthesis of *2-bromo-2-(4-chloro-2-methoxyphenyl)-1-(5-methoxy-1H-indol-3-yl) ethan-1-one (*4b*).* A stirred solution of 2-(4-chloro-2-methoxyphenyl)-1-(5-methoxy-1H-indol-3-yl) ethan-1-one (4.39 g,11.4 mmol) in THF (200 mL) was cooled to 0 °C. A solution of phenyl trimethyl ammonium tribromide ([CAS4207-56-1], 4.73 g, 12.6 mmol) in THF (100 mL) was added dropwise. The resulting suspension was stirred at room temperature for 2 h. The solids were removed by filtration and washed with THF. The combined filtrates were evaporated under reduced pressure. The residue was mixed with EtOAc (30 mL). The solids were isolated by filtration, washed with a small amount of EtOAc, and dried under vacuum at 50 °C to provide 2-bromo-2-(4-chloro-2-methoxyphenyl)-1-(5-methoxy-1H-indol-3-yl) ethan-1-one (5.0 g, yield: 94%) as a white solid, which was used in the next step without further purification.

3.Synthesis of *2-bromo-2-(4-chloro-2-methoxyphenyl)-1-(5-(2-methoxyethoxy)-1H-indol-3-yl) ethan-1-one* (4c):

Step-2c: Synthesis of *2-(4-chloro-2-methoxyphenyl)-1-(5-(2-methoxyethoxy)-1H-indol-3-yl) ethan-1-oneone (*3c*)*:

A solution of 5-(2-methoxyethoxy)-1H-indole (1 g, 1 eq.,) in DCM (150 mL) was cooled to 0 °C under a nitrogen atmosphere. A solution of diethyl aluminum chloride (1 M in hexane, 22.4 mL, 22.4 mmol) was added dropwise, and the resulting mixture was kept at 0 °C for15 min. A solution of 2-(4-chloro-2-methoxyphenyl) acetyl chloride, (1.57 g, 1.2 eq.,) in DCM (100 mL) was added dropwise. Stirring was continued at 0 °C for 1h, and the reaction mixture was subsequently stirred at room temperature for 4 h. The reaction mixture was poured out in a stirring ice/Rochelle salt solution. After the ice had melted, the mixture was filtered over dcalite and the filter cake was washed several times with tetrahydrofuran (THF). The filtrates were combined. The layers were separated, and the organic layer was washed with brine, dried over $MgSO_4$, filtered, and evaporated under reduced pressure. The residue was triturated with CH_2Cl_2 (50 mL). The resulting precipitate was filtered and dried under vacuum at 50 °C to provide 2-(4-chloro-2-methoxyphenyl)-1-(5-(2-methoxyethoxy)-1H-indol-3-yl) ethan-1-oneone (0.400 g, 20%).

Step-3c: Synthesis of *2-bromo-2-(4-chloro-2-methoxyphenyl)-1-(5-(2-methoxyethoxy)-1H-indol-3-yl) ethan-1-one* (4c). A stirred solution of 2-(4-chloro-2-methoxyphenyl)-1-(5-(2-methoxyethoxy)-1H-indol-3-yl) ethan-1-oneone (0.400 g,1 eq., 11.4 mmol) in THF (200 mL) was cooled to 0 °C. A solution of phenyl trimethyl ammonium tribromide ([CAS4207-56-1], 0.412 g, 1.01 eq.,) in THF (100 mL) was added

dropwise. The resulting suspension was stirred at room temperature for 2 h. The solids were removed by filtration and washed with THF. The combined filtrates were evaporated under reduced pressure. The residue was mixed with EtOAc (30 mL). The solids were isolated by filtration, washed with a small amount of EtOAc, and dried under vacuum at 50 °C to provide 2-bromo-2-(4-chloro-2-methoxyphenyl)-1-(5-methoxy-1H-indol-3-yl) ethan-1-one (0.15 g, yield: 24%) as a white solid, which was used in the next step without further purification.

Synthesis BNJ-278:

Synthesis of *2-(4-chloro-2-methoxyphenyl)-2-((3-chloro-5-cyclopropylphenyl) amino)-1-(5-(trifluoromethoxy)-1H-indol-3-yl) ethan-1-one*:

Step-1: Synthesis of *3-chloro-5-cyclopropylaniline* (2): A solution of 3-bromo-5-chloroaniline (0.5 g, 1 eq.,) and cyclopropylboronic acid (0.208 g, 1 eq.,), in dioxane (5 mL) and water (1 mL) was stirred at room temperature, Under nitrogen condition. To the reaction mixture was added base Cs_2CO_3 (2.367 g, 3 eq.,) and then Pd(dppf)Cl_2 (0.088 g, 0.05 eq.,) was added after purging of degas and reaction mixture was stirred for 100 ^{o}C for 8 hours. The solvent was removed under reduced pressure, and the residue was purified by flash chromatography on silica gel (gradient: 0-10% EtOAc in petroleum ether) to yield 0.19 g (46.8%) of the 3-chloro-5-cyclopropylaniline as a pale-yellow color liquid and confirmed by LCMS: = 168.0 $[M+H]^+$ at 5.2 rt.

Step-2: Synthesis of *2-(4-chloro-2-methoxyphenyl)-2-((3-chloro-5-cyclopropyl phenyl) amino)-1-(5-(trifluoromethoxy)-1H-indol-3-yl) ethan-1-one* BNJ-278: A mixture of compound 4a (*2-bromo-2-(4-chloro-2-methoxyphenyl)-1-(5-(trifluoromethoxy)-1H-indol-3-yl) ethan-1-one* (0.5 g, 1.0 eq.,), and *3-chloro-5-cyclopropylaniline* (*Int*-2), (0.272 g, 1.5 eq.,), and DIPEA (0.659 mL, 3.5 eq.,) in dry Acetonitrile (10 mL) was stirred with reflux for overnight. After cooling, the solvent was evaporated, and the residue was purified by column chromatography with silica gel (heptane/EtOH/EtOAc 1/3 from 100:0 to30:70) and obtained pure 0.256g of BNJ-278 (*2-(4-chloro-2-methoxyphenyl)-2-((3-chloro-5-cyclopropyl phenyl) amino)-1-(5-(trifluoromethoxy)-1H-indol-3-yl) ethan-1-one* as a color less solid and confirmed by LCMS = 549.1 with 91.9 % and HPLC 96.69%.

Synthesis of BNJ-390:

***Synthesis of 2-(4-chloro-2-methoxyphenyl)-1-(5-methoxy-1H-indol-3-yl)-2-((3-methoxy-5-(methylsulfonyl) phenyl) amino) ethan-1-one*:** A mixture of compound 4b (*2-bromo-2-(4-chloro-2-methoxy phenyl)-1-(5-methoxy-1H-indol-3-yl)ethan-1-one)* (0.08 g, 1.0 eq.,), *3-methoxy-5-(methyl sulfonyl) aniline (5)*, (0.05 g, 1.3 eq.,), and DIPEA (0.119 mL, 3.5 eq.,) in dry Acetonitrile (3 mL) was stirred and reflux for overnight. After cooling, the solvent was evaporated, and the residue was purified by column chromatography on silica gel (heptane/EtOH/EtOAc 1/3 from 100:0 to30:70) to afford *2-(4-chloro-2-methoxyphenyl)-1-(5-methoxy-1H-indol-3-yl)-2-((3-*

methoxy-5-(methylsulfonyl) phenyl) amino) ethan-1-one, C (5.2 mg, yield: 25 % pure) as a color less solid and confirmed by LCMS = 529.2 with 94.12 % and HPLC 95.19 %.

Synthesis of BNJ-391:

***Synthesis of 2-(4-chloro-2-methoxyphenyl)-2-((3-methoxy-5-(methylsulfonyl)phenyl) amino)-1-(5-(2-methoxyethoxy)-1H-indol-3-yl) ethan-1-one*:** A mixture of compound 3c (*2-bromo-2-(4-chloro-2-methoxyphenyl)-1-(5-(2-methoxyethoxy)-1H-indol-3-yl) ethan-1-one* (4c*)* (0.08 g, 1.0 eq.,), *3-methoxy-5-(methylsulfonyl) aniline (*5*)*, (0.050 g, 1.3 eq.,), and DIPEA (0.119 mL, 3.5 eq.,) in dry Acetonitrile (2 mL) was stirred and reflux for overnight. After cooling, the solvent was evaporated, and the residue was purified by column chromatography on silica gel (heptane/EtOH/EtOAc 1/3 from 100:0 to 30:70) to afford *BNJ-392 (2-(4-chloro-2-methoxyphenyl)-2-((3-methoxy-5-(methylsulfonyl) phenyl) amino)-1-(5-(2-methoxyethoxy)-1H-indol-3-yl) ethan-1-one,* (5.2 mg, yield: 25 % pure) as a color less solid and confirmed by LCMS = 573.2 with 94.12 % and HPLC 95.19 %.

Synthesis of BNJ-495:

***Synthesis of (3-amino-5-methoxyphenyl)dimethylphosphine oxide (2)*:** To a solution of *3-bromo-5-methoxyaniline* (0.3 g, 1 eq.,) and *dimethyl phosphine oxide* (0.151 g, 1.5 eq) in DMF (15 mL), was added K_3PO_4 (0.66 g, 2.5 eq.,) was at RT under nitrogen purging for 10 mints. Then *xantphos* (0.086 g, 0.12 eq.,) and Pd $(OAc)_2$ (0.027 g, 0.1 eq.,) was added to reaction mixture stirred at 100 ^{0} C for 8 hours. The solvent was removed under reduced pressure, and the residue was purified by flash chromatography on silica gel (gradient: 0-30% EtOAc in petroleum ether) to yield 0.150 g (50%) of *(3-amino-5-methoxyphenyl)dimethylphosphine oxide* as a solid. LCMS = 230.77 $[M+H]^+$.

Step-2: Synthesis of *2-(4-chloro-2-methoxyphenyl)-2-((3-(dimethylphosphoryl)-5-methoxyphenyl) amino)-1-(5-(trifluoromethoxy)-1H-indol-3-yl)ethan-1-one (BNJ-495)*: A mixture of *2-bromo-2-(4-chloro-2-methoxyphenyl)-1-(5-(trifluoromethoxy)-1H-indol-3-yl) ethan-1-one* (*4a)* (0.1 g, 1.0 eq.,), *(3-amino-5-methoxyphenyl) dimethyl phosphine (2)*, (0.08 g, 1.3 eq.,), and DIPEA (0.12 mL, 3.5 eq.,) in dry Acetonitrile (10 mL) was stirred at reflux overnight. After cooling, the solvent was evaporated, and the residue was purified by column chromatography on silica gel (heptane/EtOH/EtOAc 1/3 from 100:0 to30:70) to afford *2-(4-chloro-2-methoxyphenyl)-2-((3-(dimethylphosphoryl)-5-methoxyphenyl)amino)-1-(5-(trifluoromethoxy)-1H-indol-3-yl)ethan-1-one (BNJ-495)* (0.0120 g, yield: 22 % pure) as a color less solid and confirmed by LCMS = 581.2 with 91.9 % and HPLC 96.69 %.

LCMS of compound BNJ-278

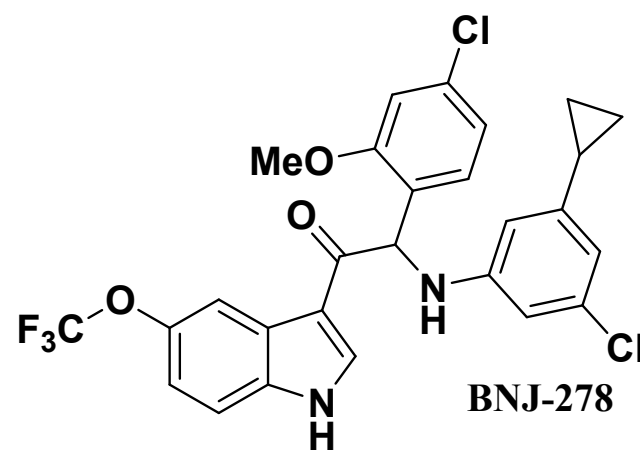

BNJ-278

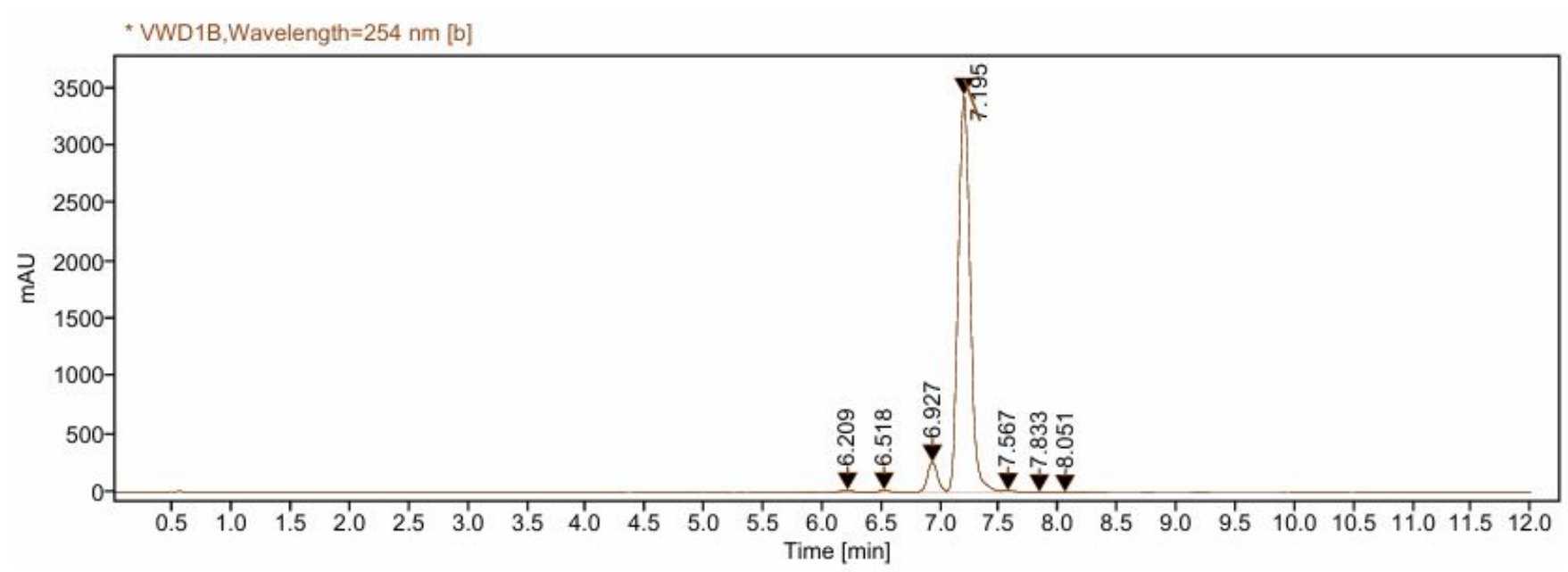

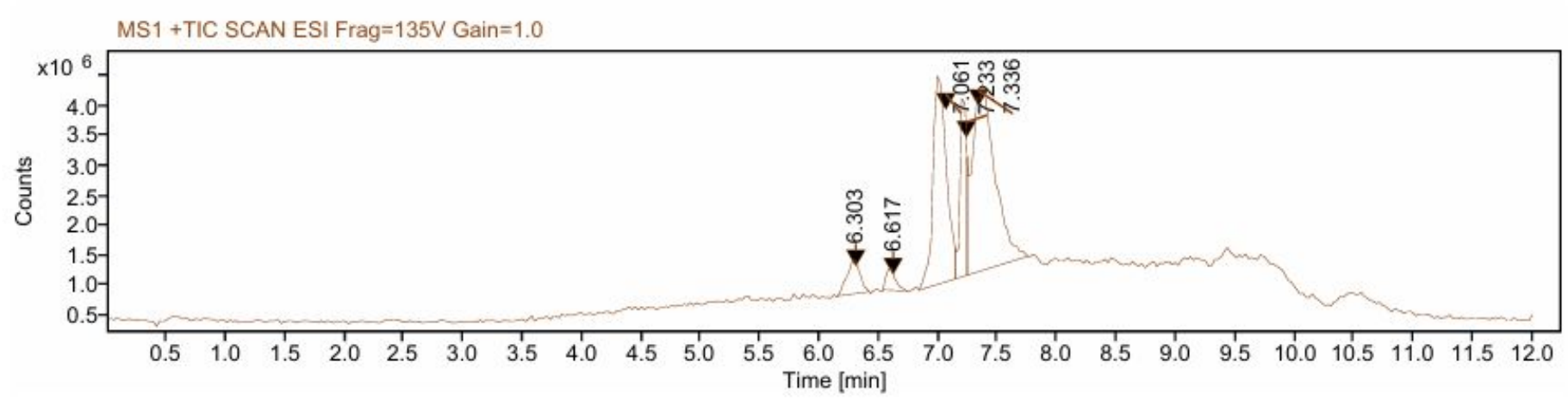

Signal: * VWD1B,Wavelength=254 nm [b]

RT [min]	Area	Area%
6.209	178.5889	0.6802
6.518	147.0574	0.5601
6.927	1616.3369	6.1561
7.195	24147.6469	91.9699
7.567	128.9226	0.4910
7.833	25.3038	0.0964
8.051	12.1825	0.0464

Peak RT: 7.233

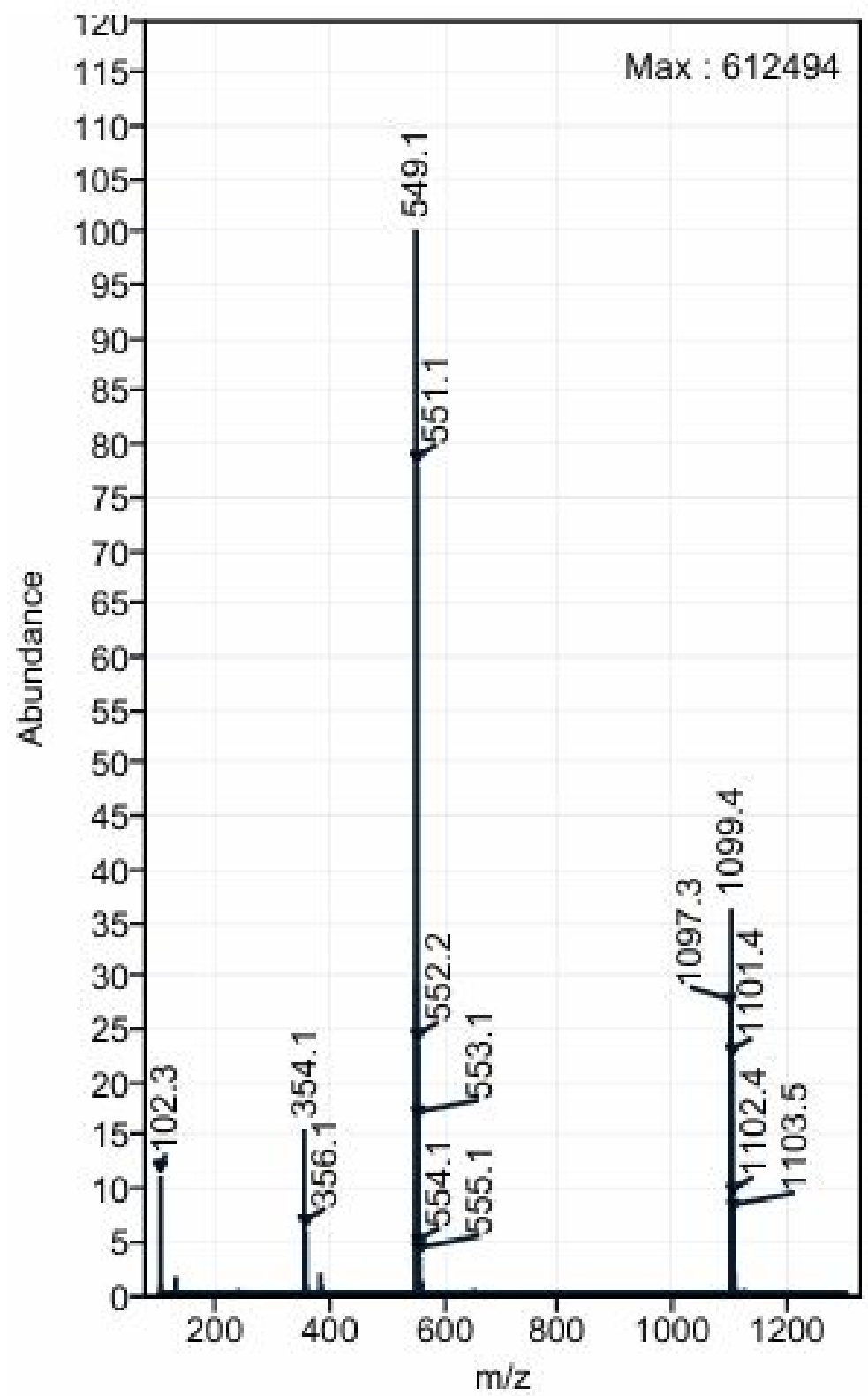

Peak RT: 7.336

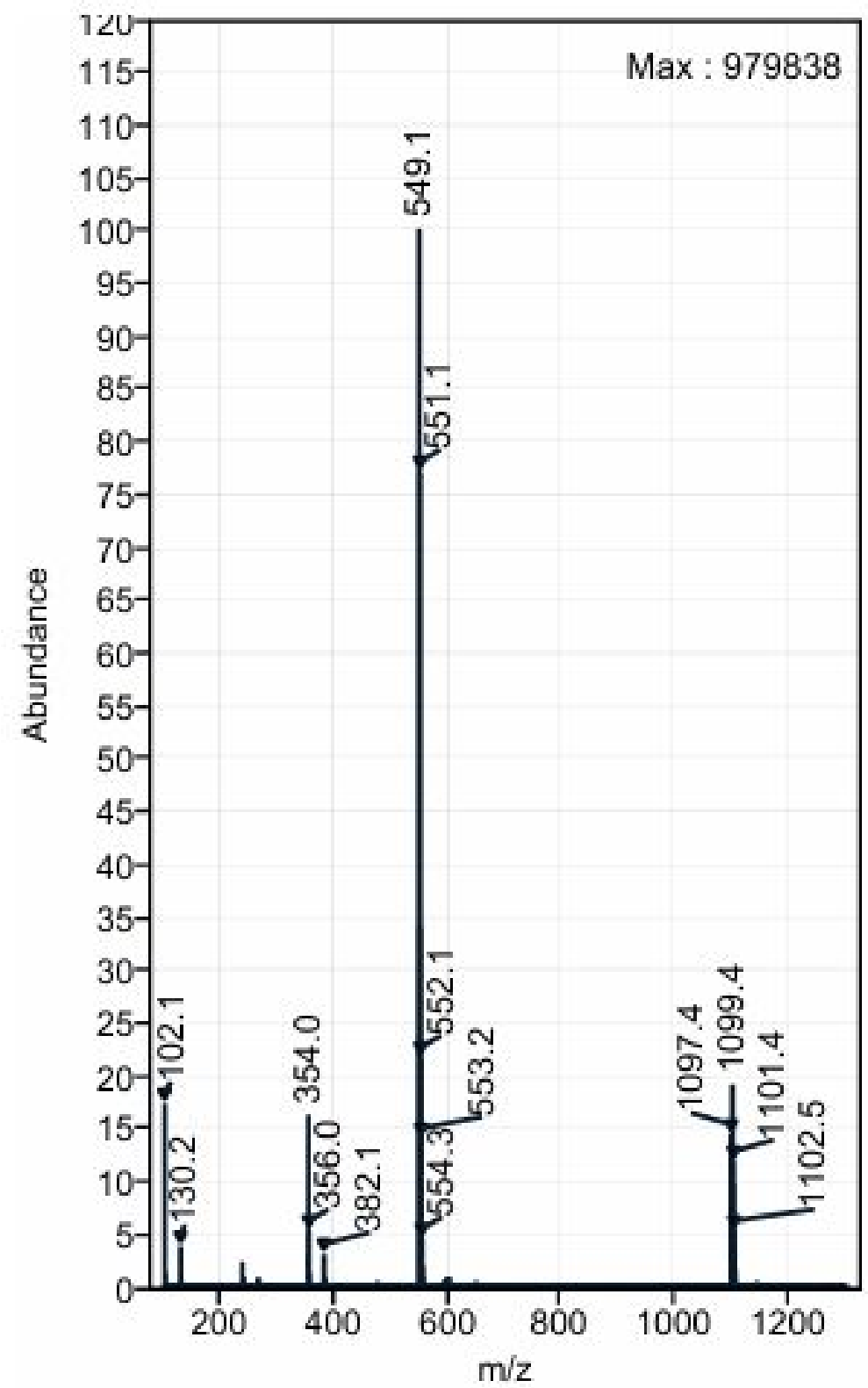

HPLC of Compound BNJ-278

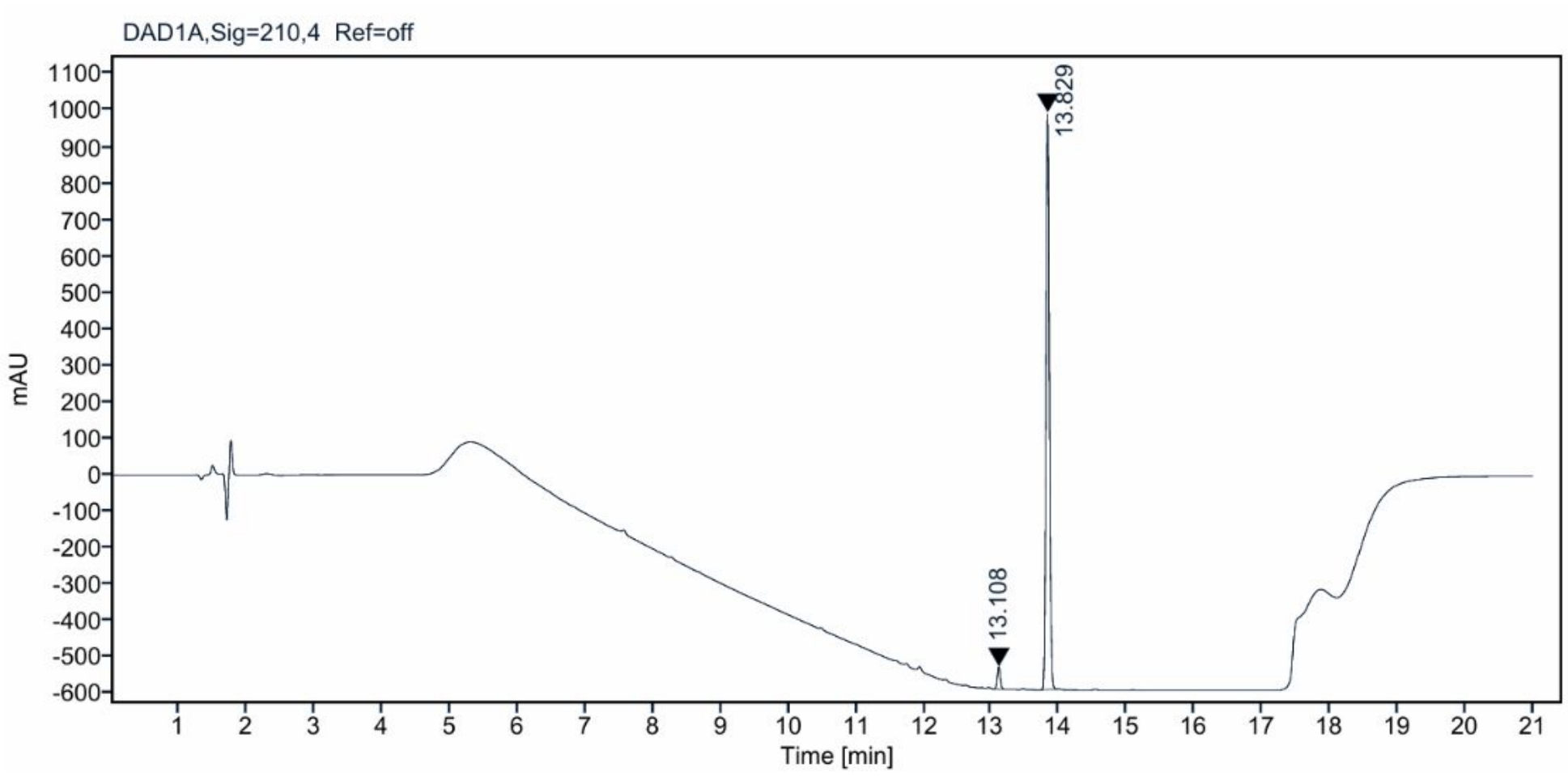

Signal: DAD1A,Sig=210,4 Ref=off

RT [min]	Area	Height	Area%
13.108	190.1027	61.7954	3.3059
13.829	5560.2551	1582.3737	96.6941
Sum	5750.3577		

^{1}HNMR of Compound BNJ-278

^{1}H NMR (600 MHz, DMSO) δ 12.275 (s, 1H), 8.550 (s, 1H), 8.059 (d, J = 1.24 Hz, 1H), 7.585 (d, J = 8.74 Hz, 1H), 7.348 (d, J = 8.29 Hz, 1H), 7.222 – 7.193 (m, 1H), 7.122 (d, J = 2.00 Hz, 1H), 6.974 (dd, J = 8.30, 2.01 Hz, 1H), 6.640 (d, J = 8.05 Hz, 1H), 6.553 (t, J = 2.00 Hz, 1H), 6.369 (s, 1H), 6.287 (s, 1H), 6.193 (d, J = 8.01 Hz, 1H), 3.990 (s, 3H), 1.758 – 1.706 (m, 1H), 0.886 – 0.853 (m, 2H), 0.638 – 0.592 (m, 1H), 0.519 – 0.474 (m, 1H).

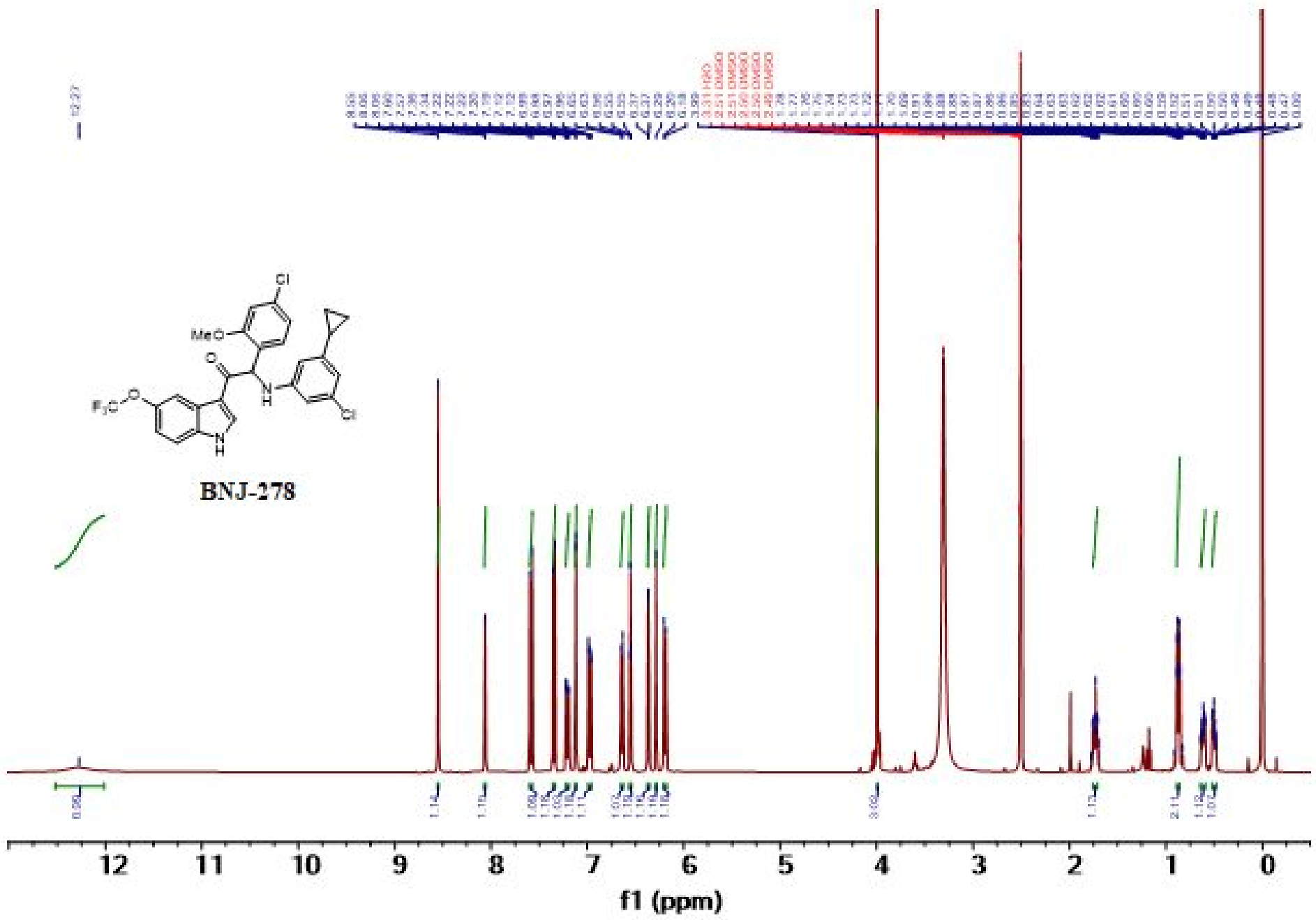

LCMS of Compound BNJ-390

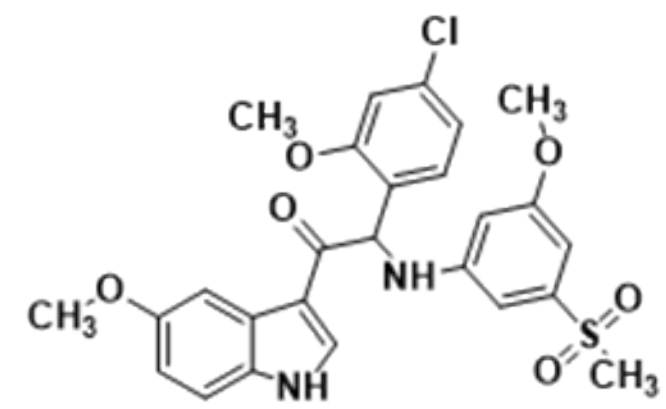

BNJ-390

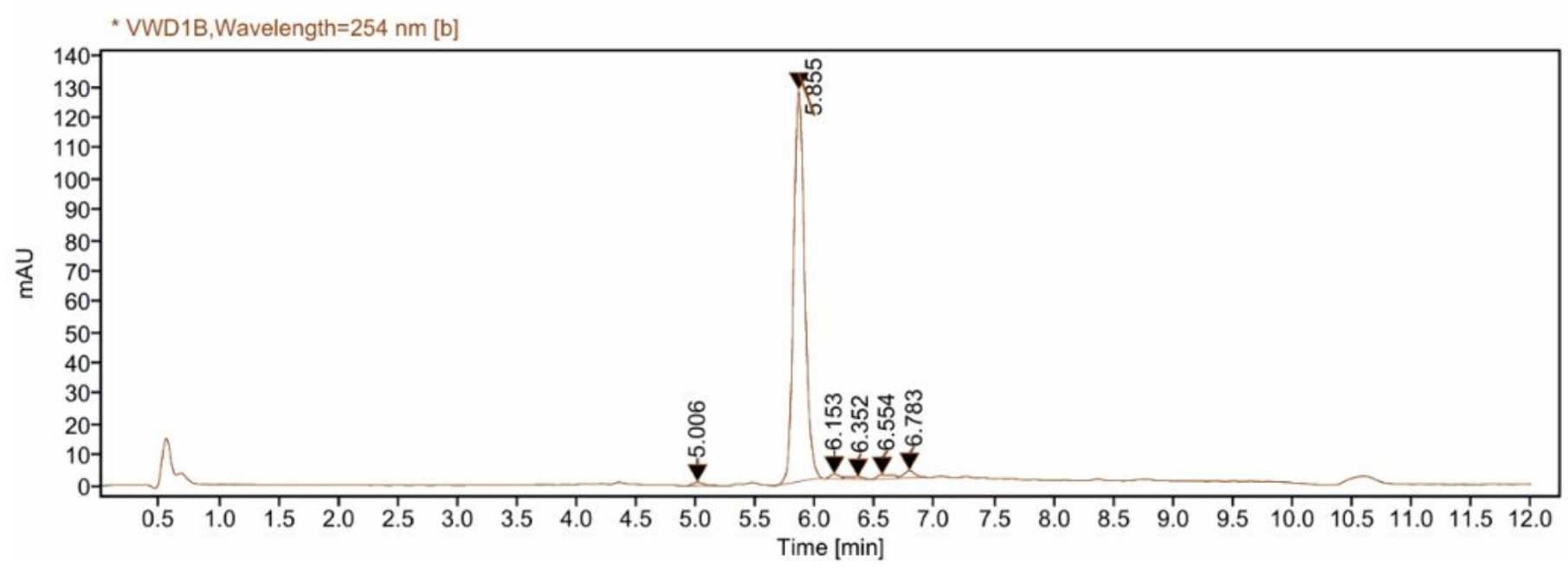

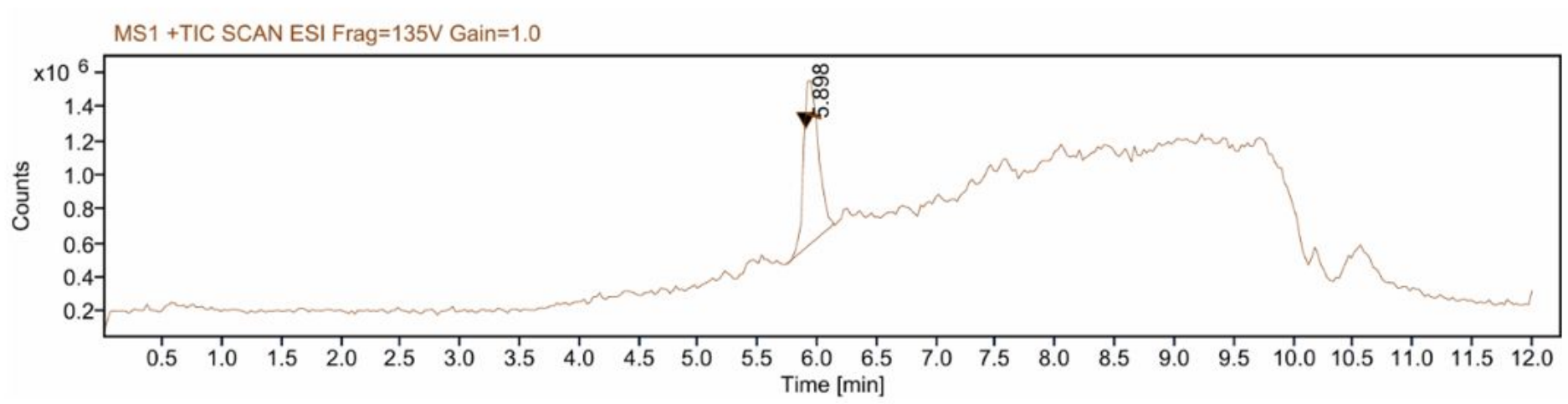

Signal: * VWD1B,Wavelength=254 nm [b]

RT [min]	Area	Area%
5.006	7.4537	0.8576
5.855	818.0834	94.1278
6.153	8.1319	0.9357
6.352	8.3498	0.9607
6.554	12.3238	1.4180
6.783	14.7770	1.7002
**Sum**	**869.1197**	

Peak RT: 5.898

Signal: MS1 +TIC SCAN ESI Frag=135V Gain=1.0

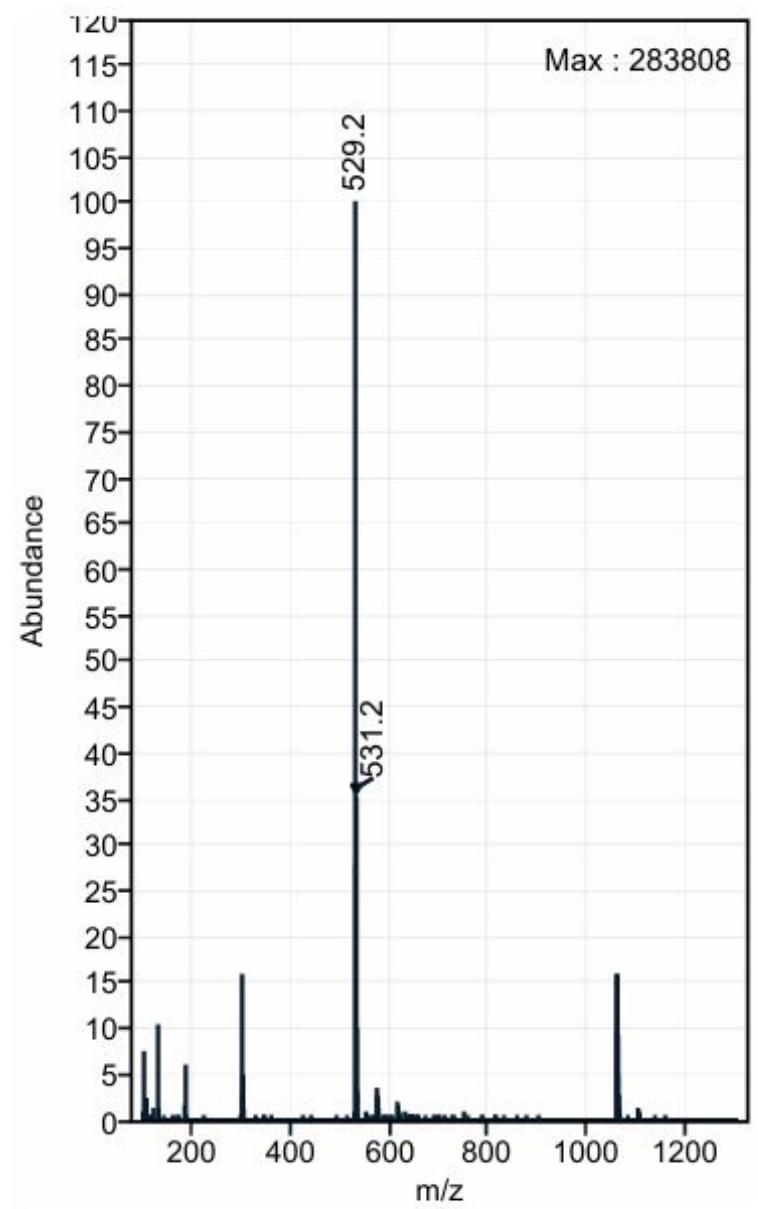

HPLC of Compound BNJ-390

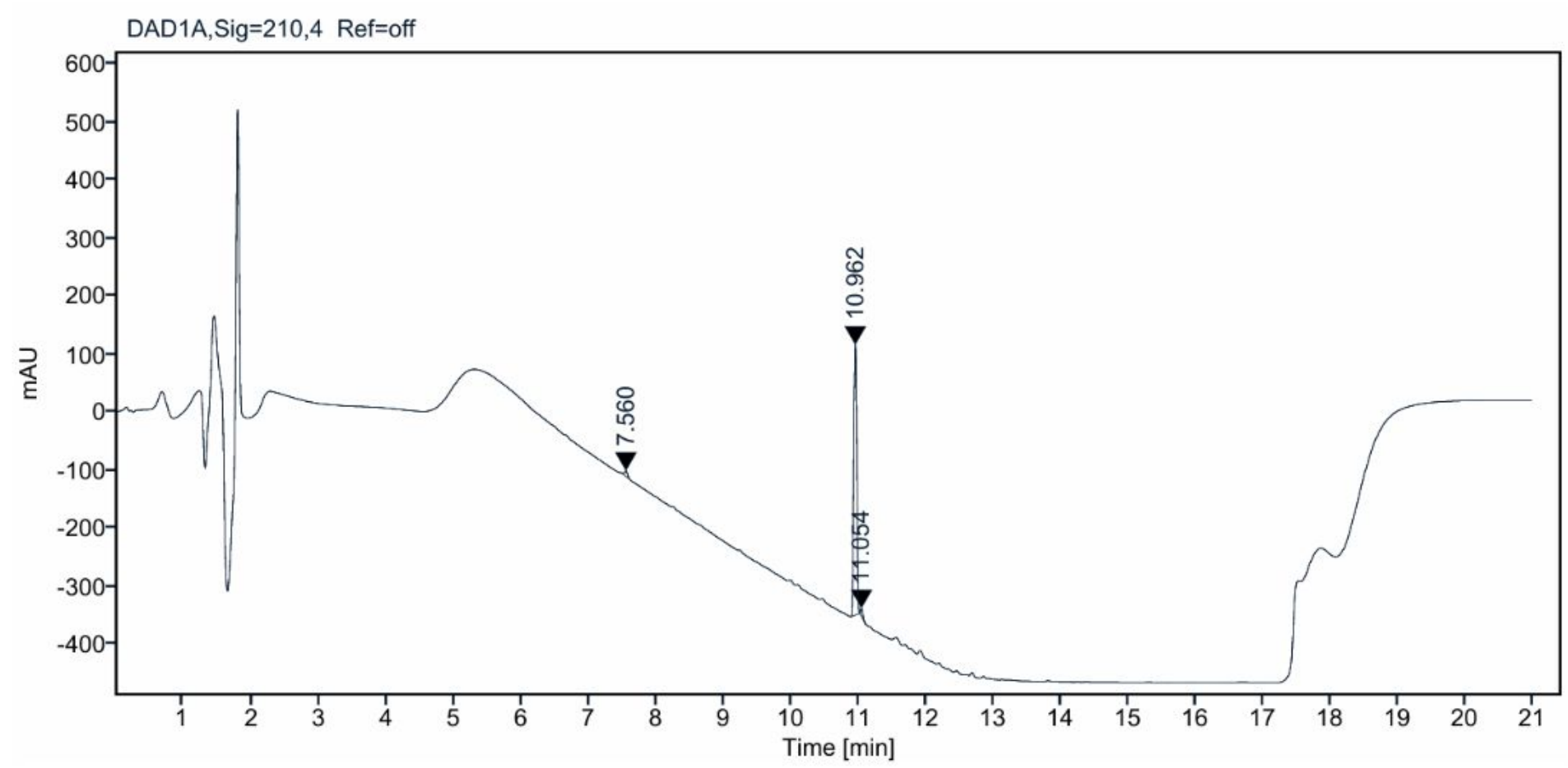

Signal: DAD1A,Sig=210,4 Ref=off

RT [min]	Area	Height	Area%
7.560	32.0842	9.3722	2.2590
10.962	1351.9948	466.0572	95.1928
11.054	36.1918	14.2838	2.5482
Sum	1420.2708		

LCMS of Compound BNJ-391

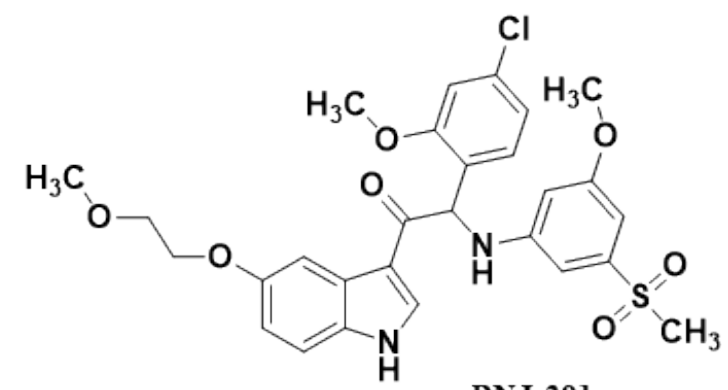

BNJ-391

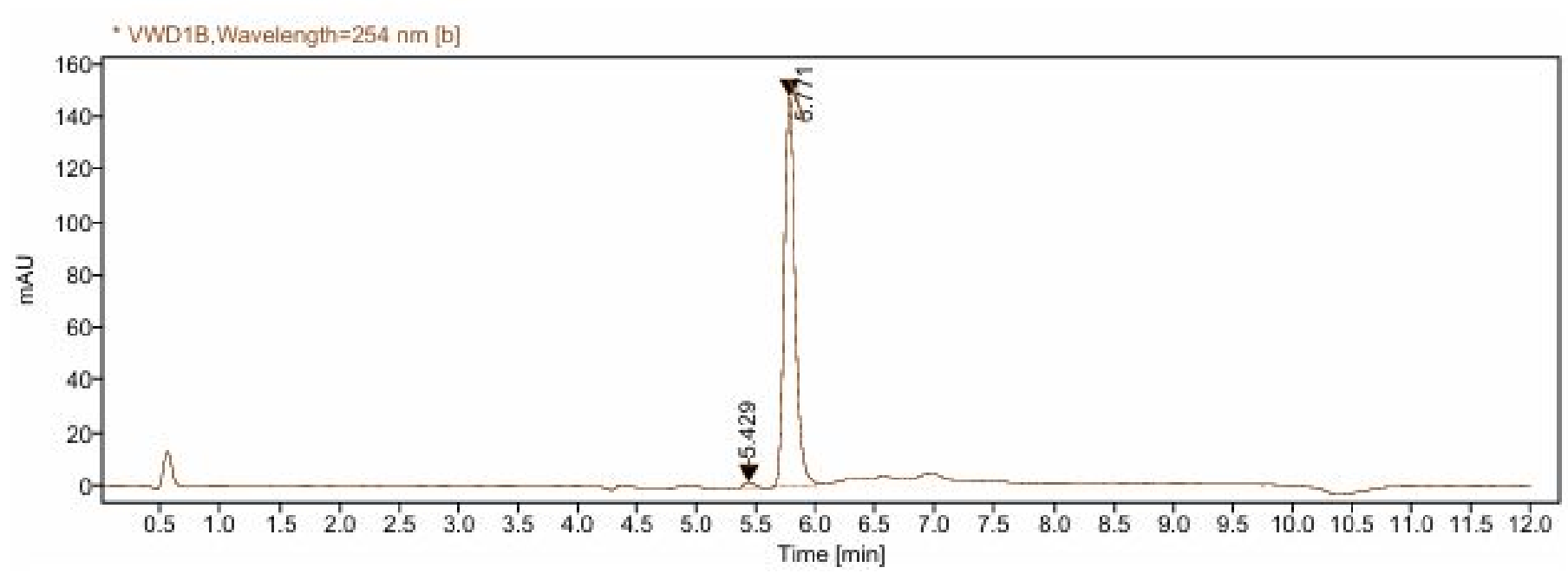

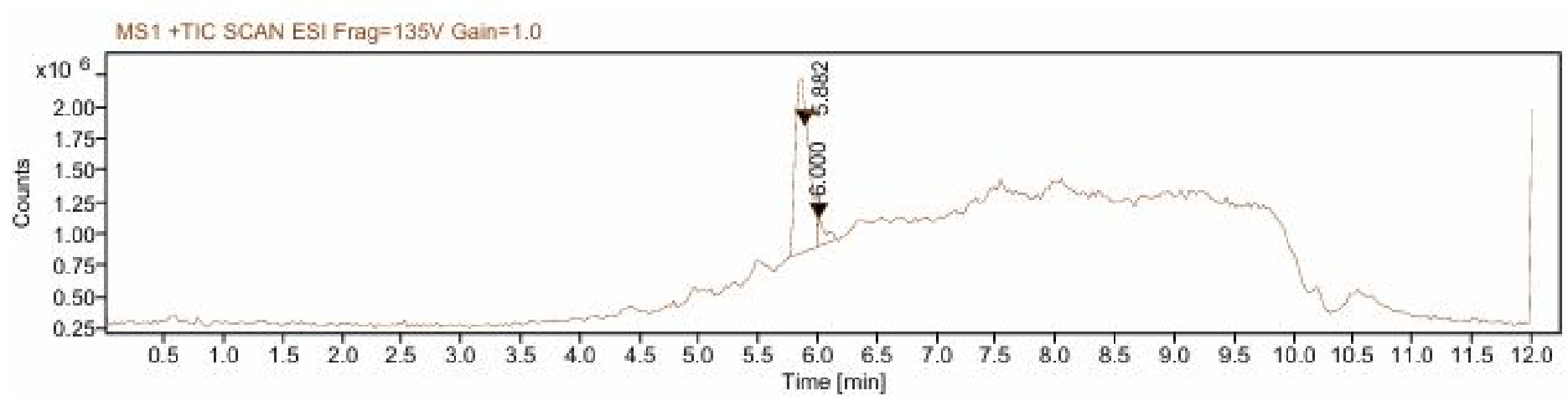

Signal: * VWD1B,Wavelength=254 nm [b]

RT [min]	Area	Area%
5.429	11.7501	1.2300
5.771	943.5792	98.7700
**Sum**	**955.3293**	

Peak RT: 5.882 **Peak RT:** 6.000

Signal: MS1 +TIC SCAN ESI Frag=135V Gain=1.0

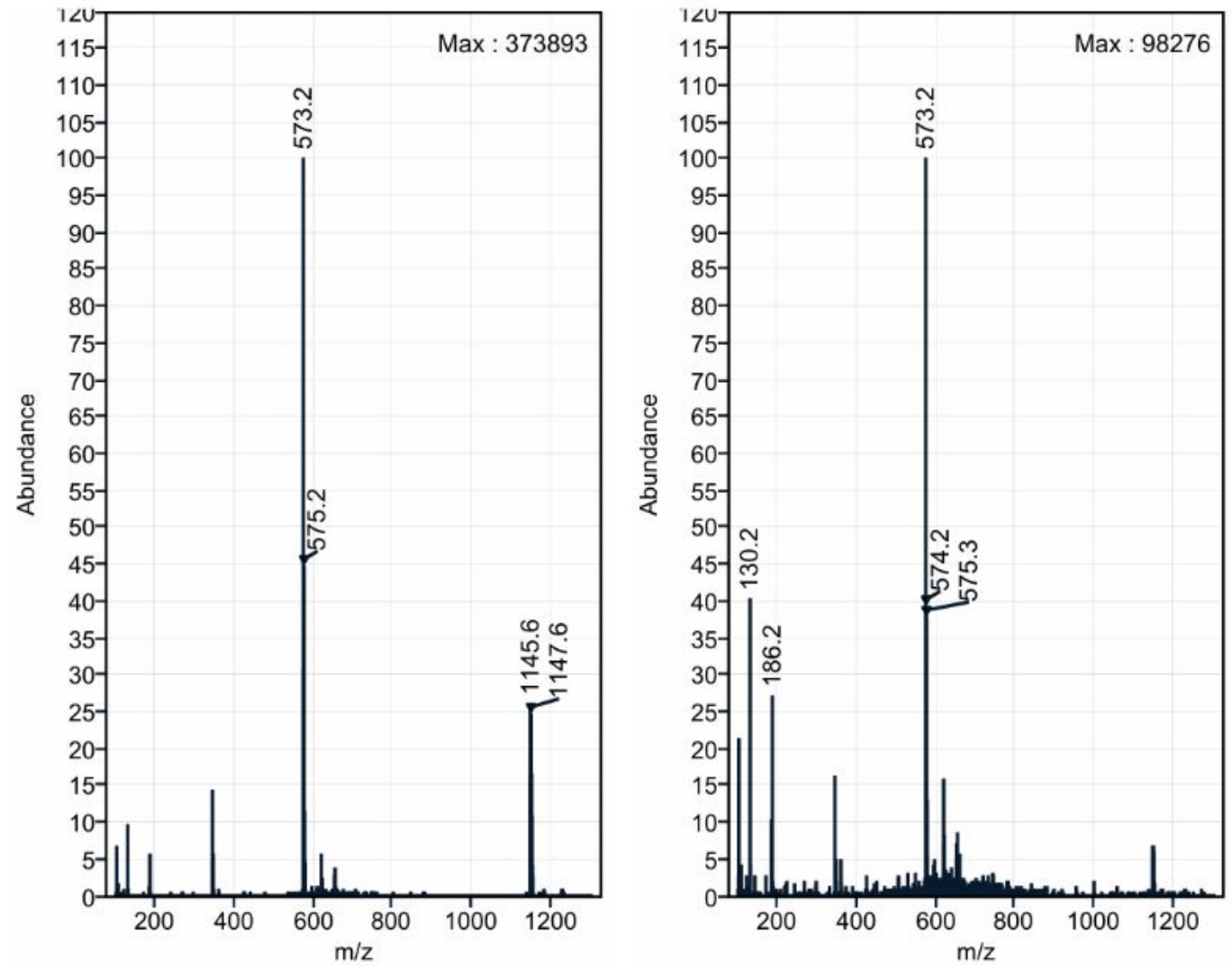

HPLC of Compound BNJ-495

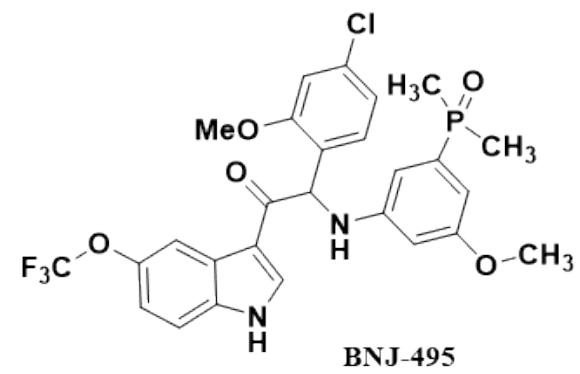

BNJ-495

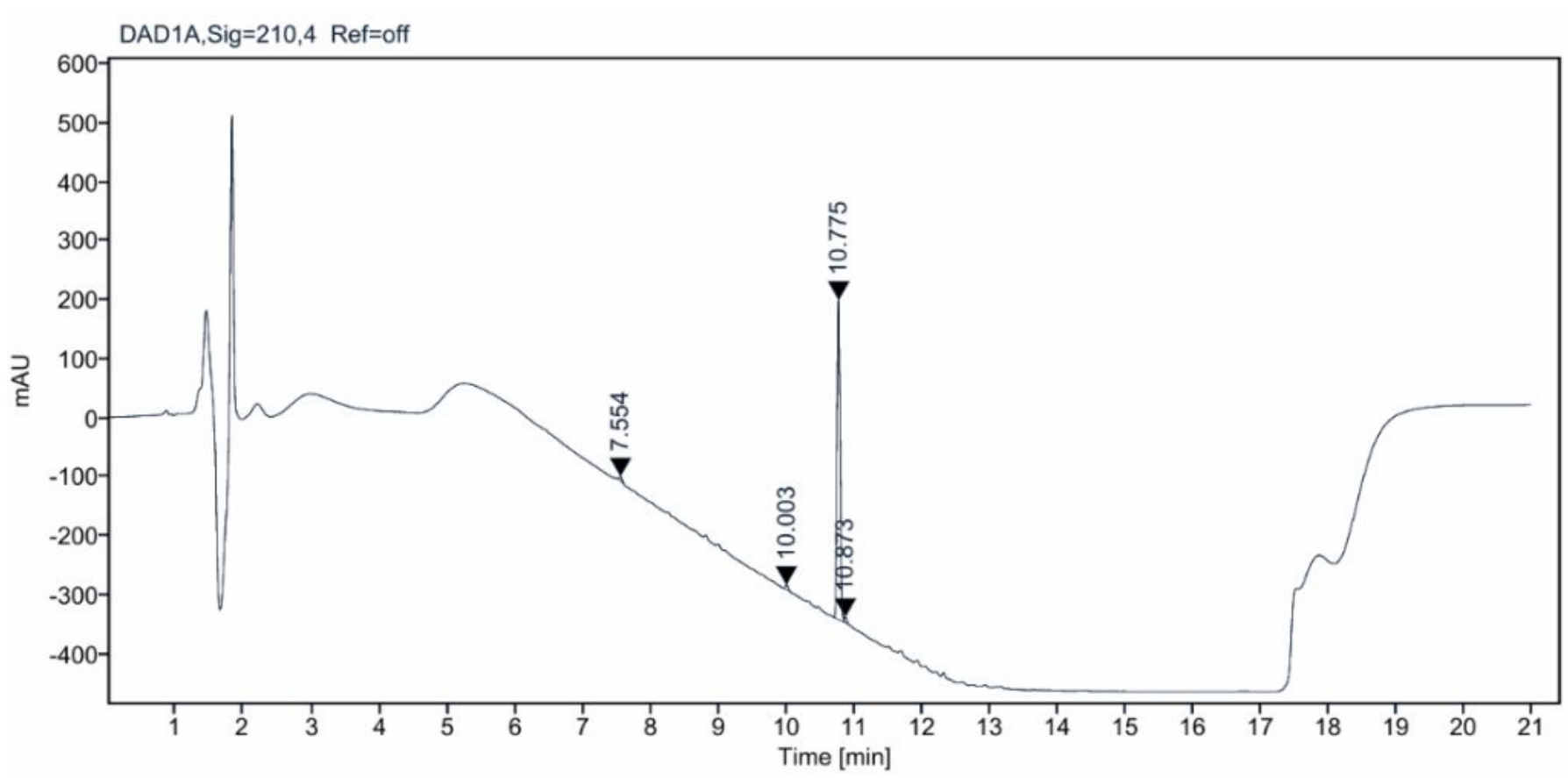

Signal: DAD1A,Sig=210,4 Ref=off

RT [min]	Area	Height	Area%
7.554	19.9500	7.2493	1.1846
10.003	23.1179	7.8388	1.3727
10.775	1615.3456	541.7743	95.9168
10.873	25.6984	9.5281	1.5259
Sum	1684.1120		

^{1}HNMR of Compound BNJ-391

^{1}H NMR (600 MHz, DMSO) δ 11.935 (d, J = 3.276 Hz, 1H), 8.352 (d, J = 3.305 Hz, 1H), 7.680 (d, J = 2.543 Hz, 1H), 7.363 (dd, J = 8.551, 2.321 Hz, 2H), 7.123 (d, J = 2.064 Hz, 1H), 7.024 (d, J = 7.727 Hz, 1H), 6.972 (dd, J = 8.328, 1.986 Hz, 1H), 6.910 (t, J = 1.781 Hz, 1H), 6.861 (dd, J = 8.831, 2.545 Hz, 1H), 6.582 (p, J = 2.204 Hz, 2H), 6.213 (d, J = 7.629 Hz, 1H), 4.092 – 4.060 (m, 2H), 4.003 (s, 3H), 3.724 (s, 3H), 3.687 – 3.659 (m, 2H), 3.318 (s, 3H), 3.087 (s, 3H).

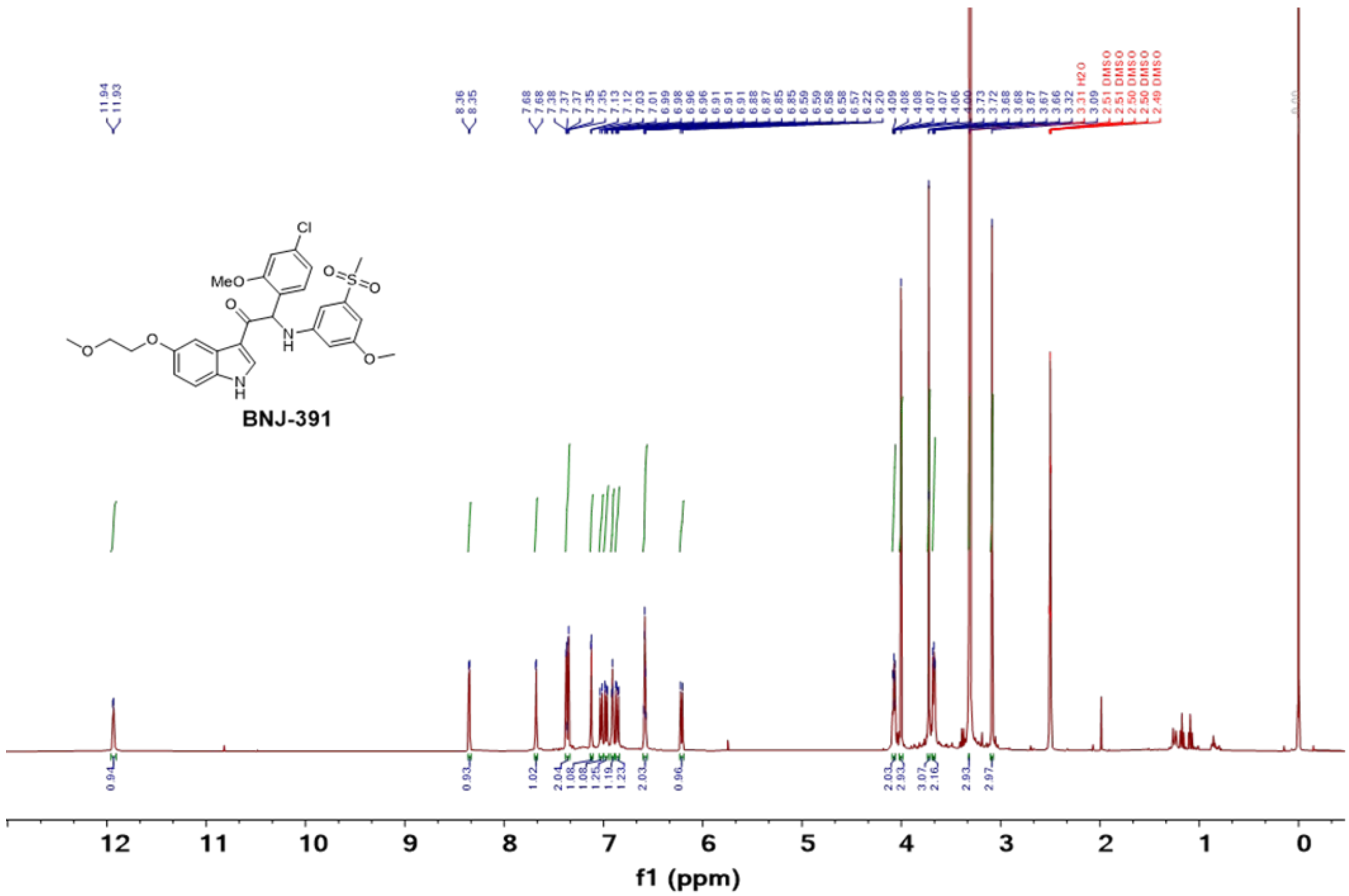

LCMS of Compound BNJ-495

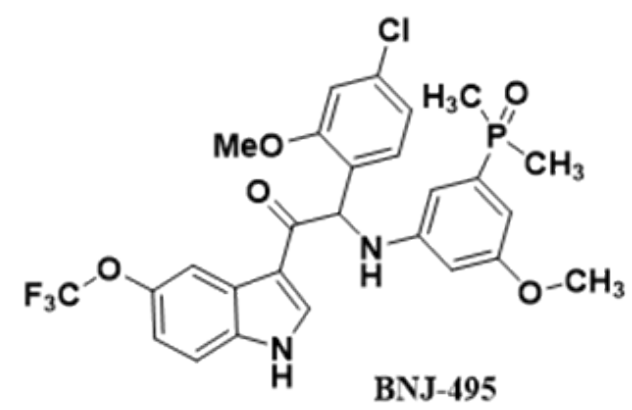

BNJ-495

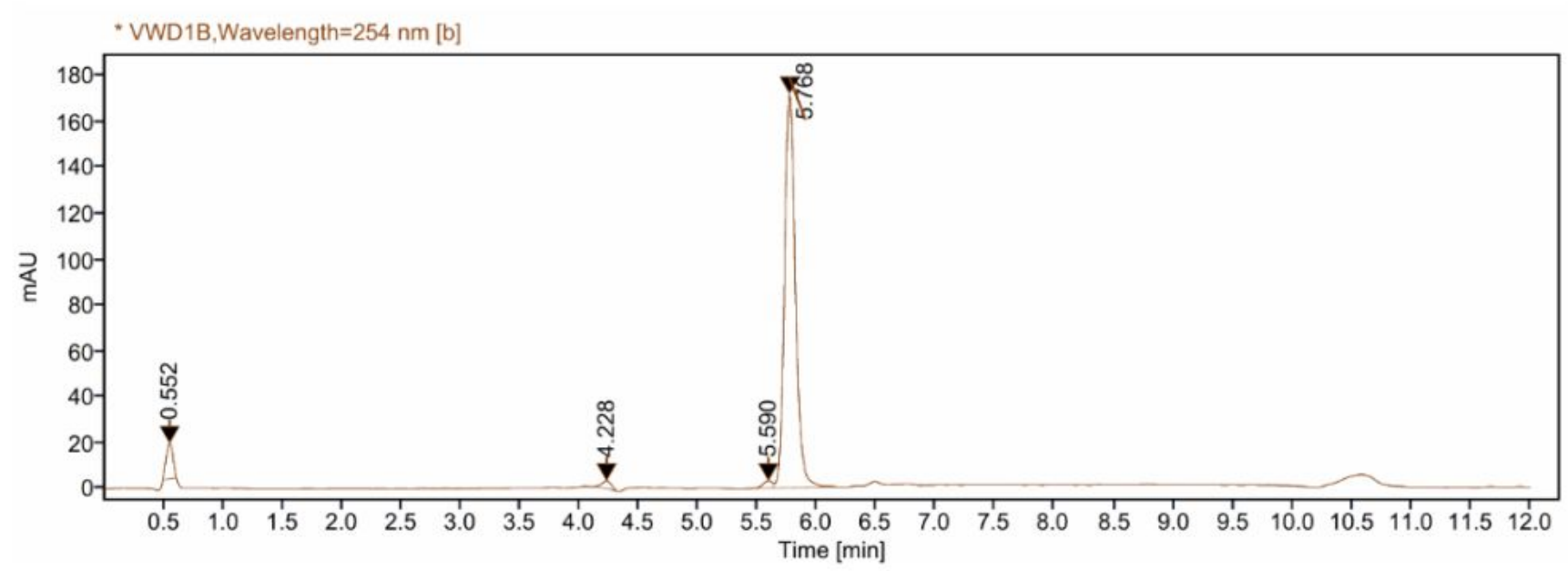

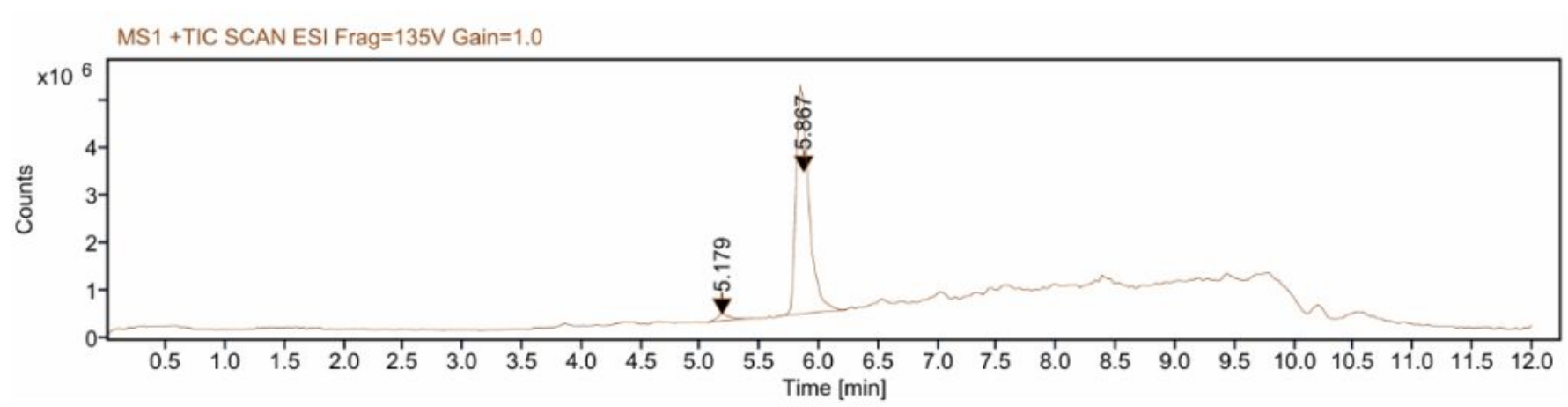

Signal: * VWD1B,Wavelength=254 nm [b]

RT [min]	Area	Area%
0.552	60.8367	5.0910
4.228	17.6910	1.4804
5.590	17.5483	1.4685
5.768	1098.9150	91.9601
**Sum**	**1194.9910**	

Peak RT: 5.179 **Peak RT:** 5.867

Signal: MS1 +TIC SCAN ESI Frag=135V Gain=1.0

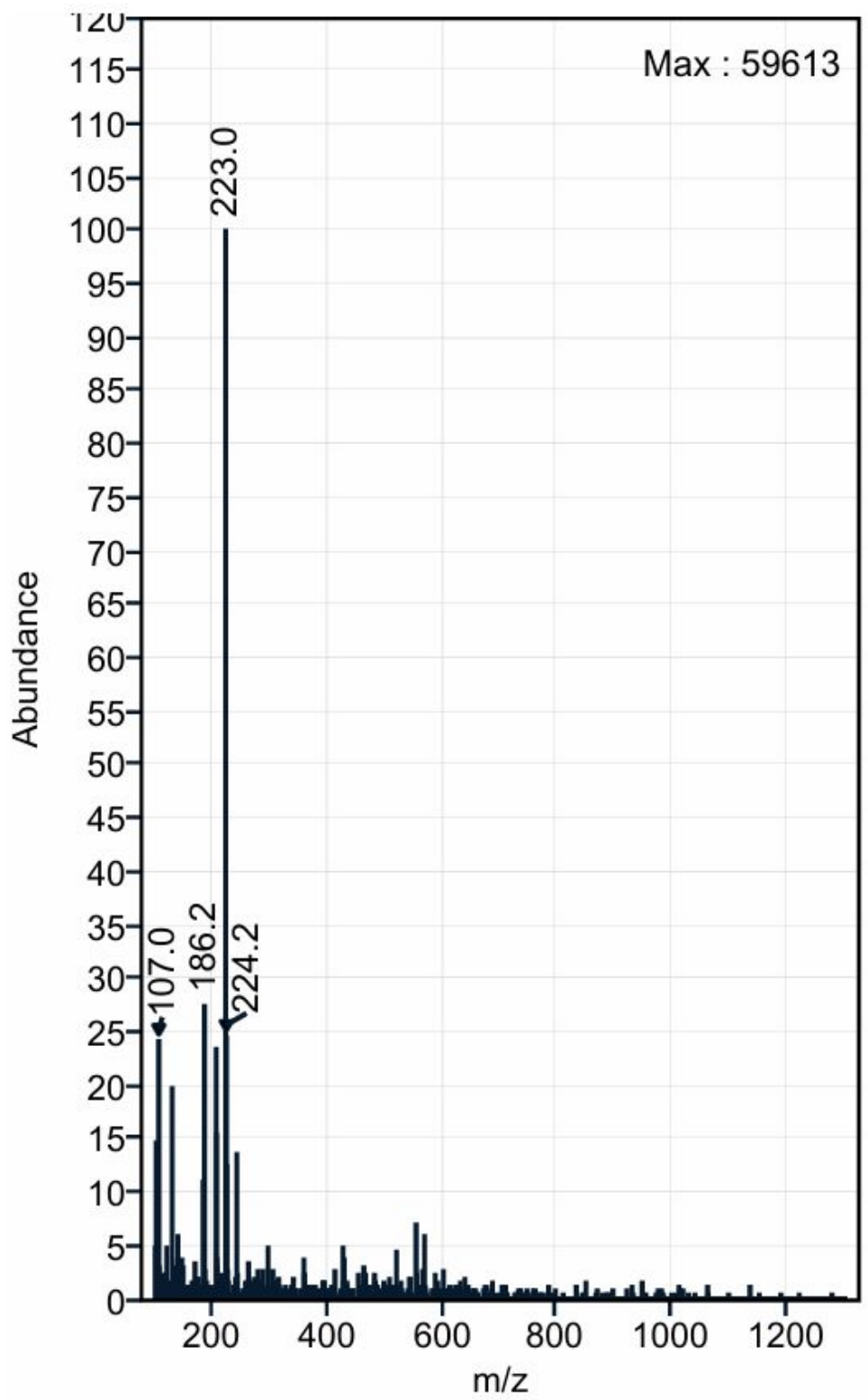

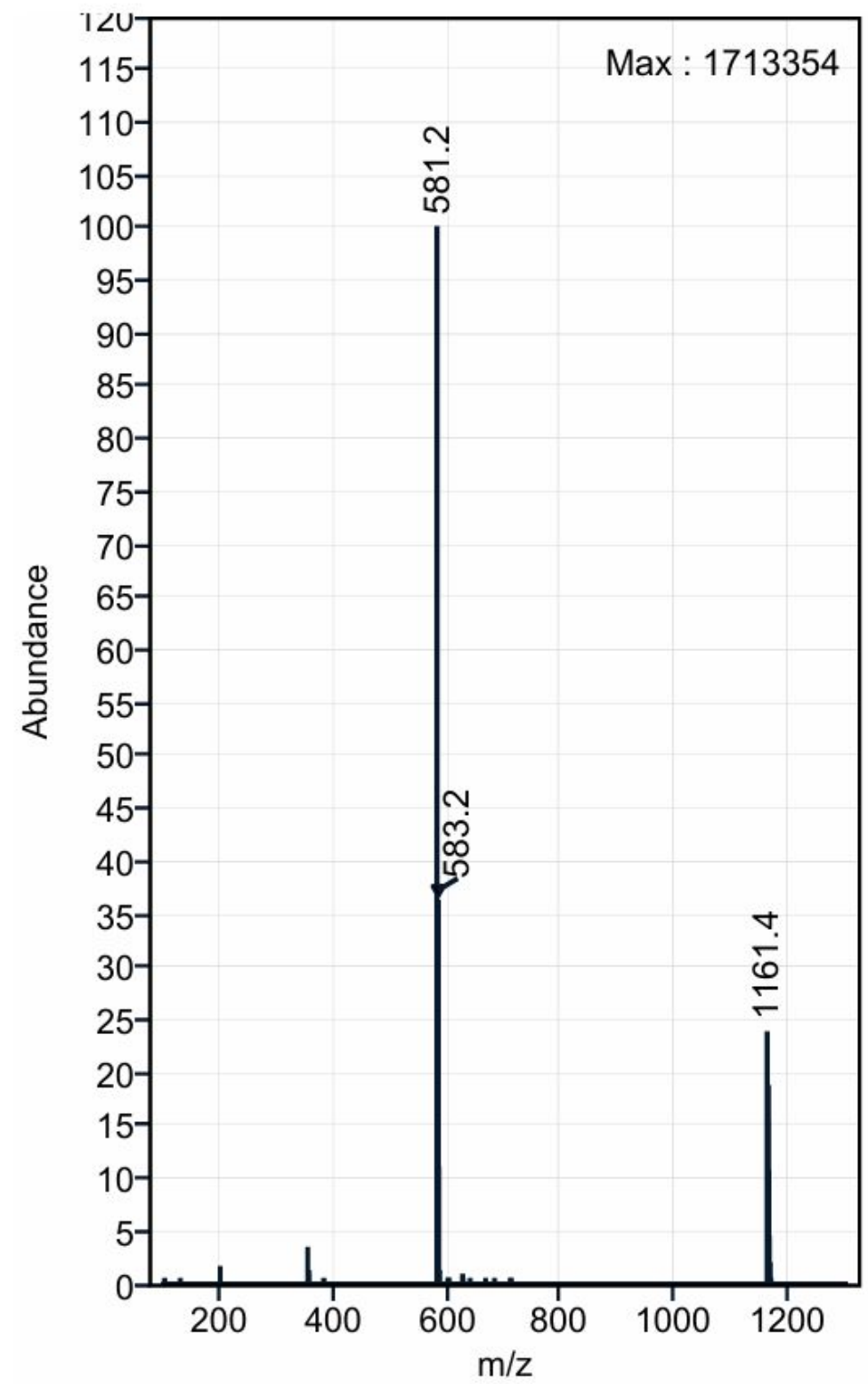

<u>**HPLC of Compound BNJ-495**</u>

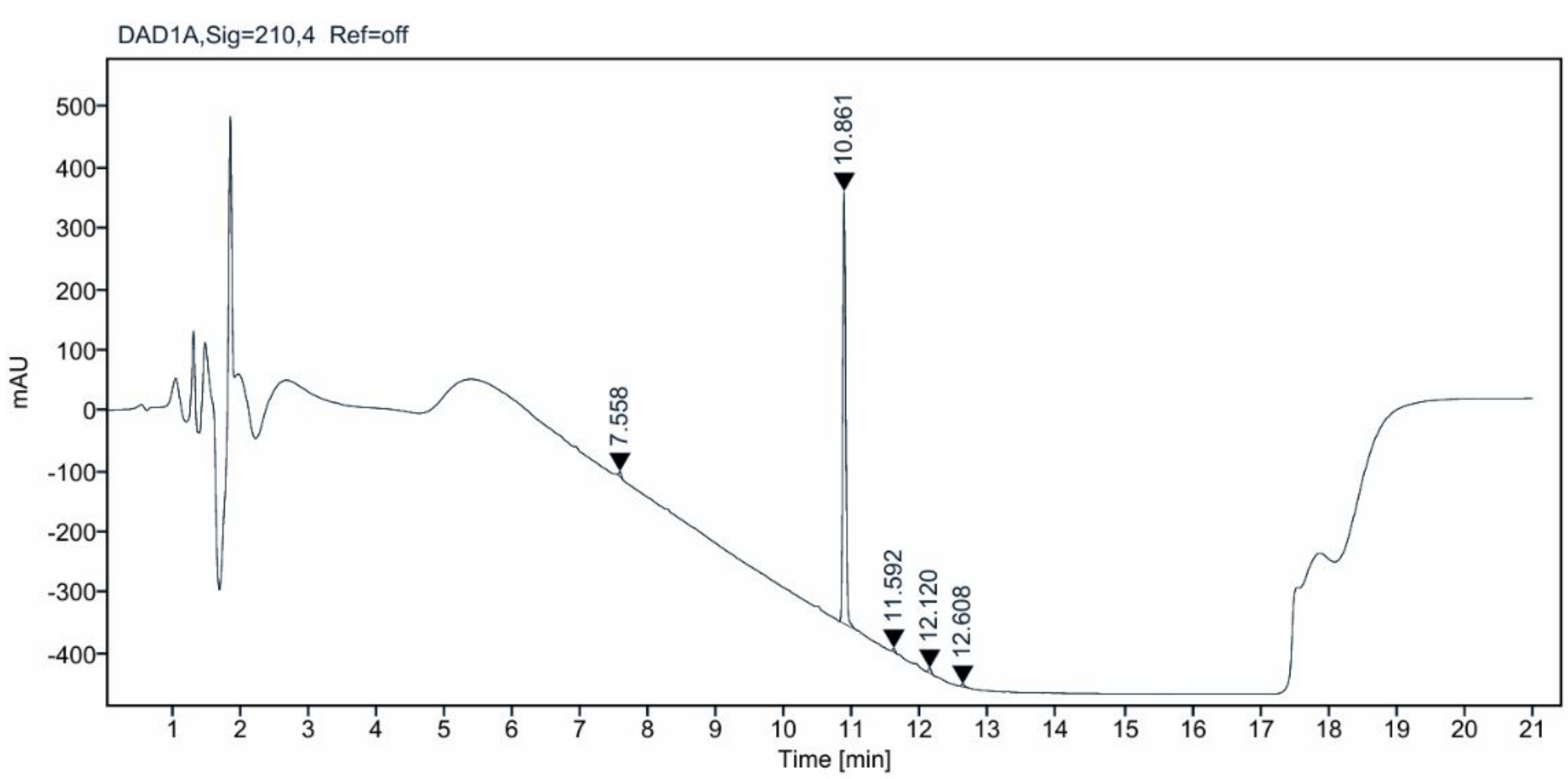

Signal: DAD1A,Sig=210,4 Ref=off

RT [min]	Area	Height	Area%
7.558	22.9894	8.3572	0.9974
10.861	2228.9540	712.4715	96.6998
11.592	16.6625	6.2798	0.7229
12.120	23.1069	8.1601	1.0025
12.608	13.3123	4.2903	0.5775
Sum	2305.0251		

LCMS of Intermediate Int-2

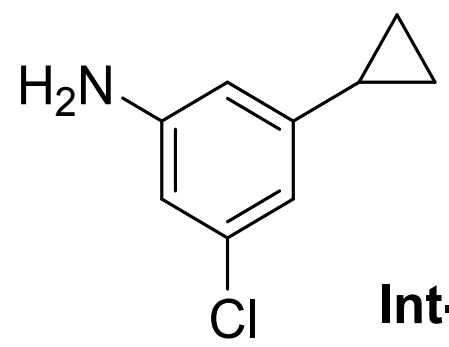

Int-2

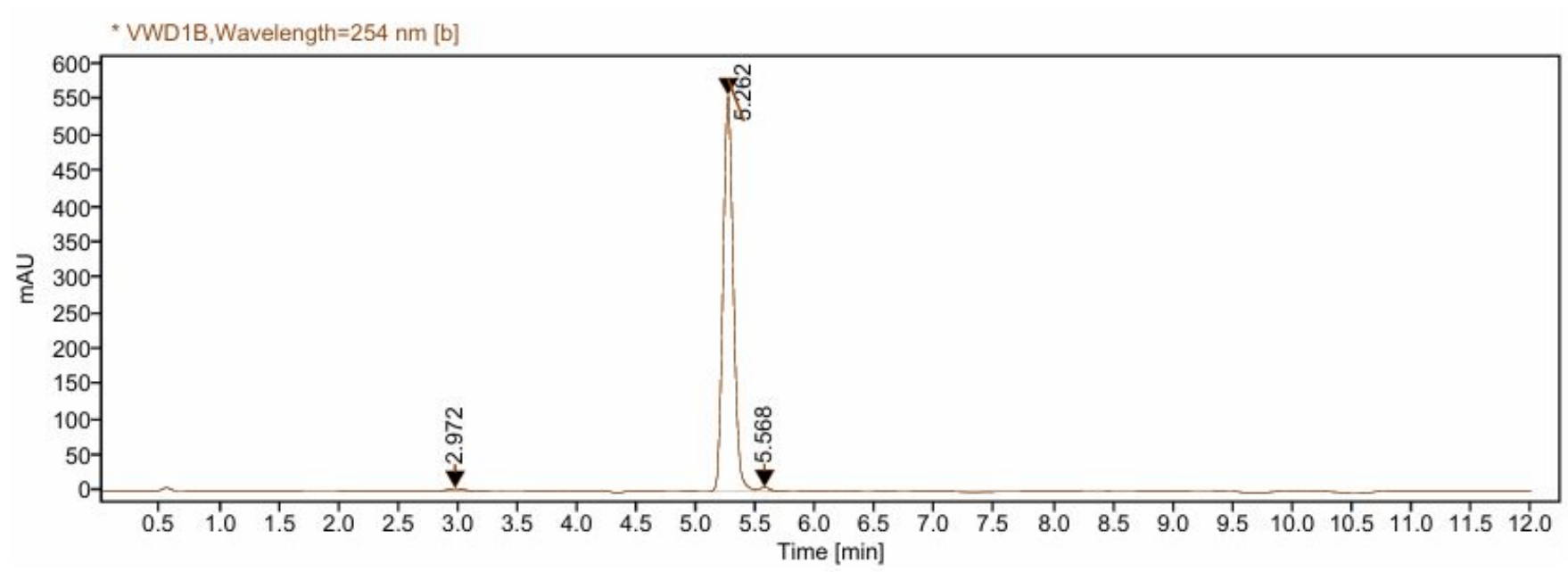

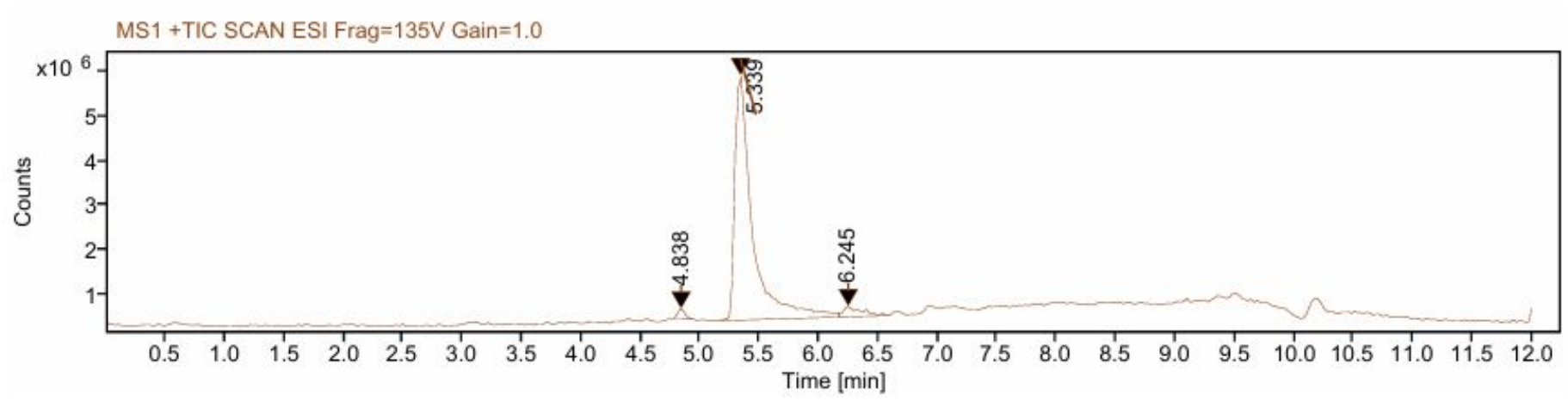

Signal: * VWD1B,Wavelength=254 nm [b]

RT [min]	Area	Area%
2.972	30.2075	0.8934
5.262	3320.8536	98.2202
5.568	29.9690	0.8864
**Sum**	**3381.0300**	

Peak RT: 4.838 **Peak RT:** 5.339 **Peak RT:** 6.245

Signal: MS1 +TIC SCAN ESI Frag=135V Gain=1.0

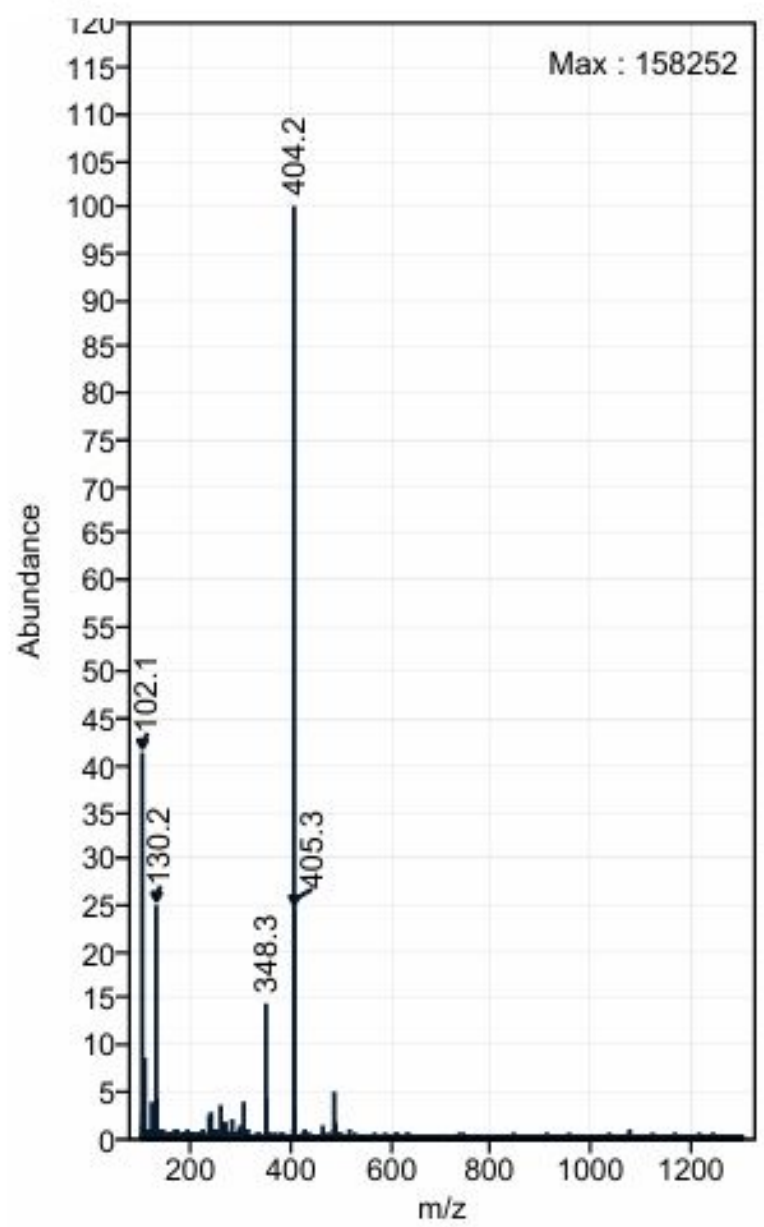

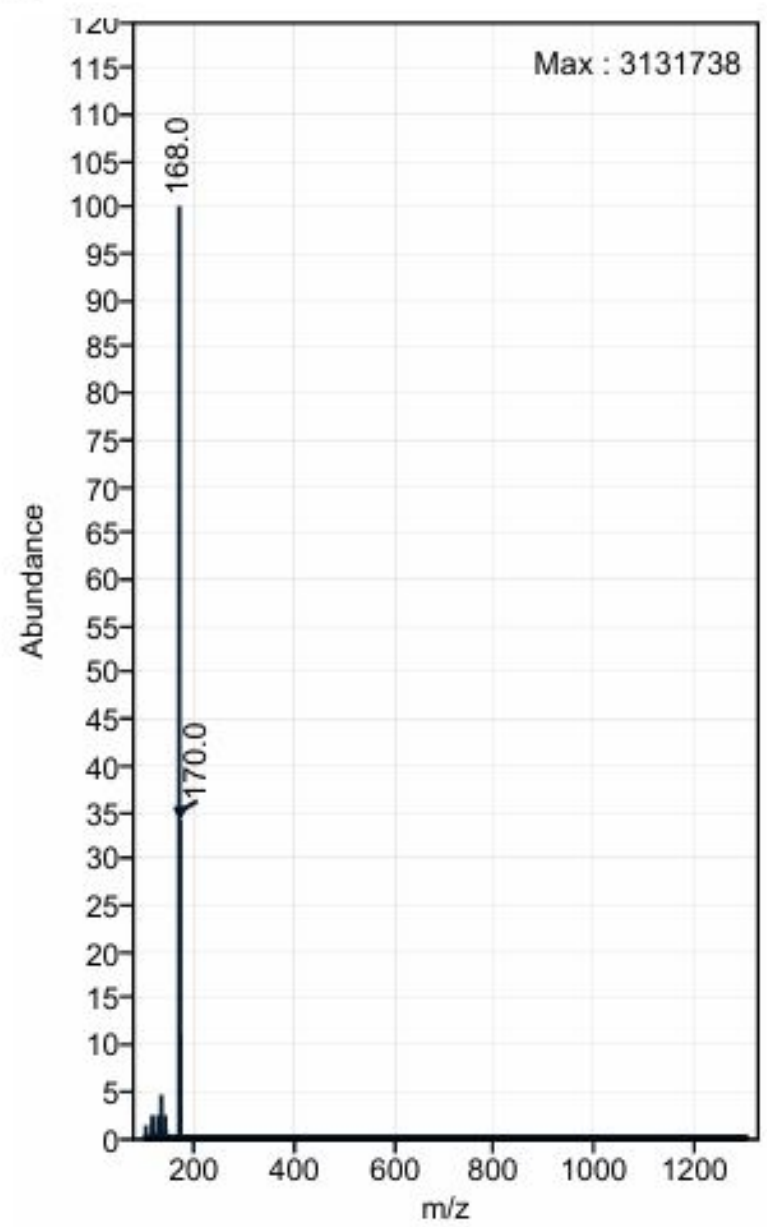

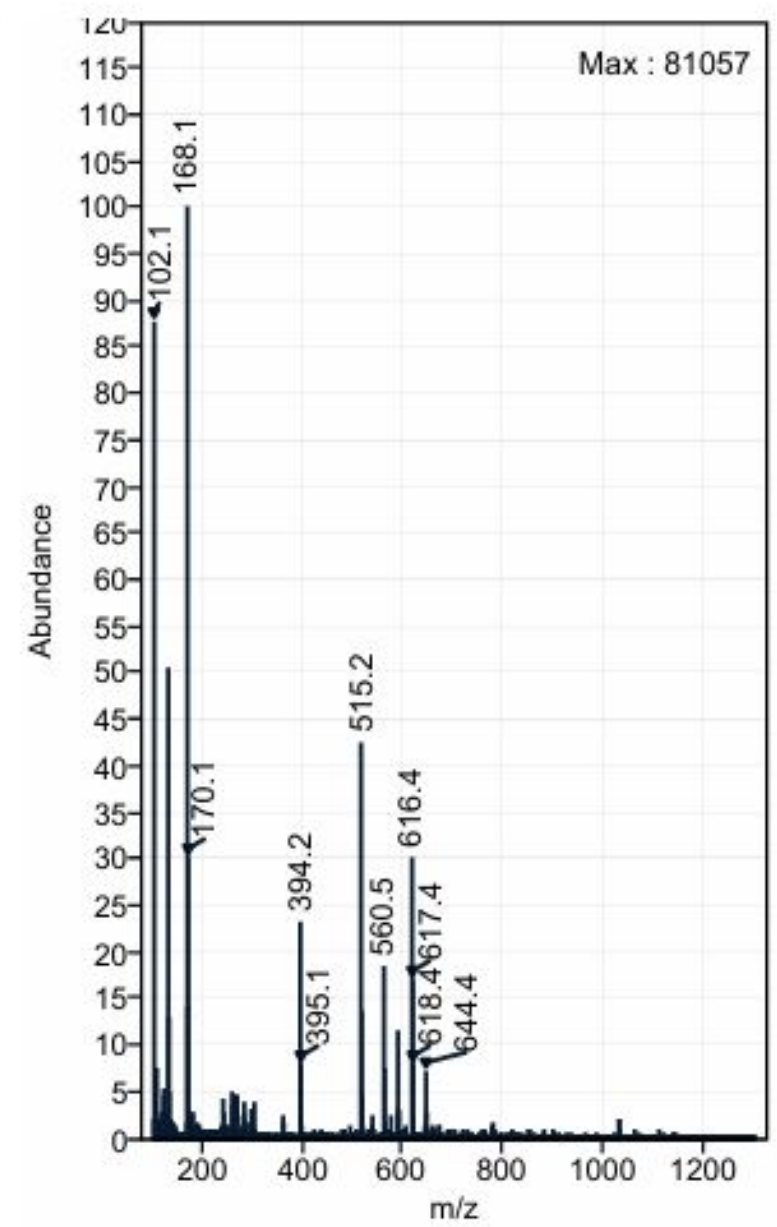

^{1}HNMR of Intermediate 2c

^{1}H NMR (600 MHz, DMSO-D6) δ 10.851 (s, 1H), 7.241 – 7.223 (m, 2H), 6.994 (s, 1H), 6.688 (dt, J = 8.720, 1.302 Hz, 1H), 6.285 – 6.272 (m, 1H), 4.019 (d, J = 4.719 Hz, 2H), 3.619 (d, J = 1.902 Hz, 2H), 3.302 (s, 3H).

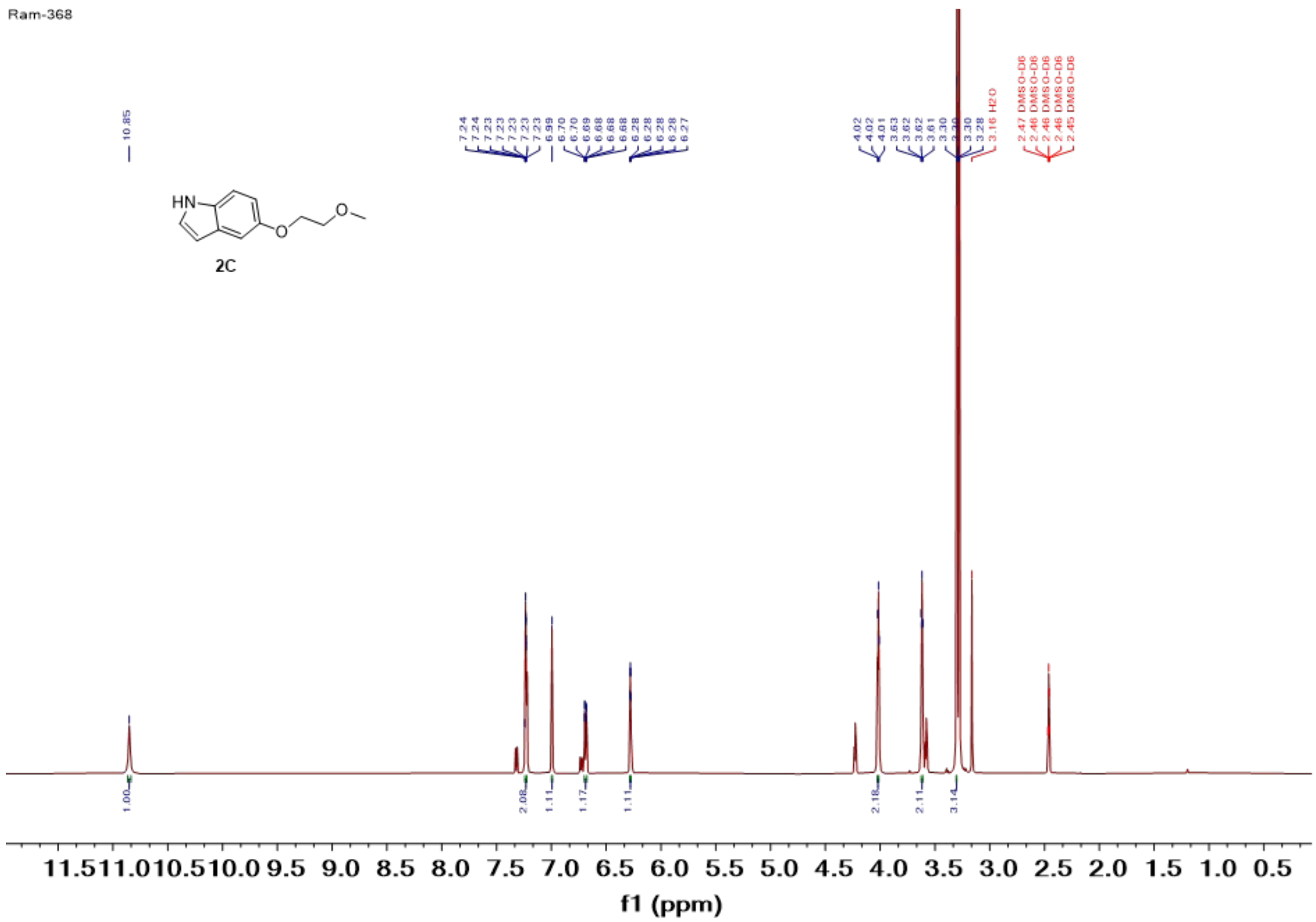

LCMS of Intermediate 3a

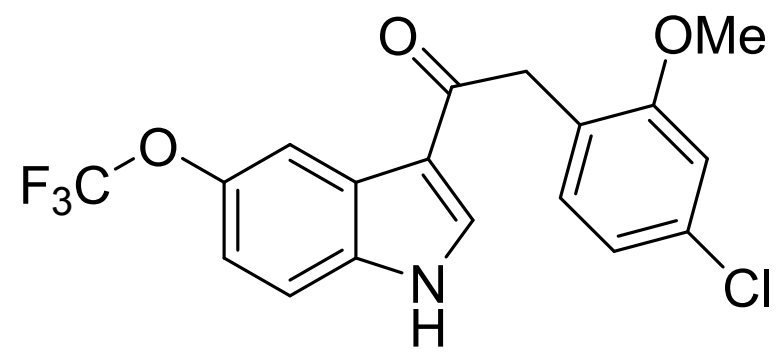

3a

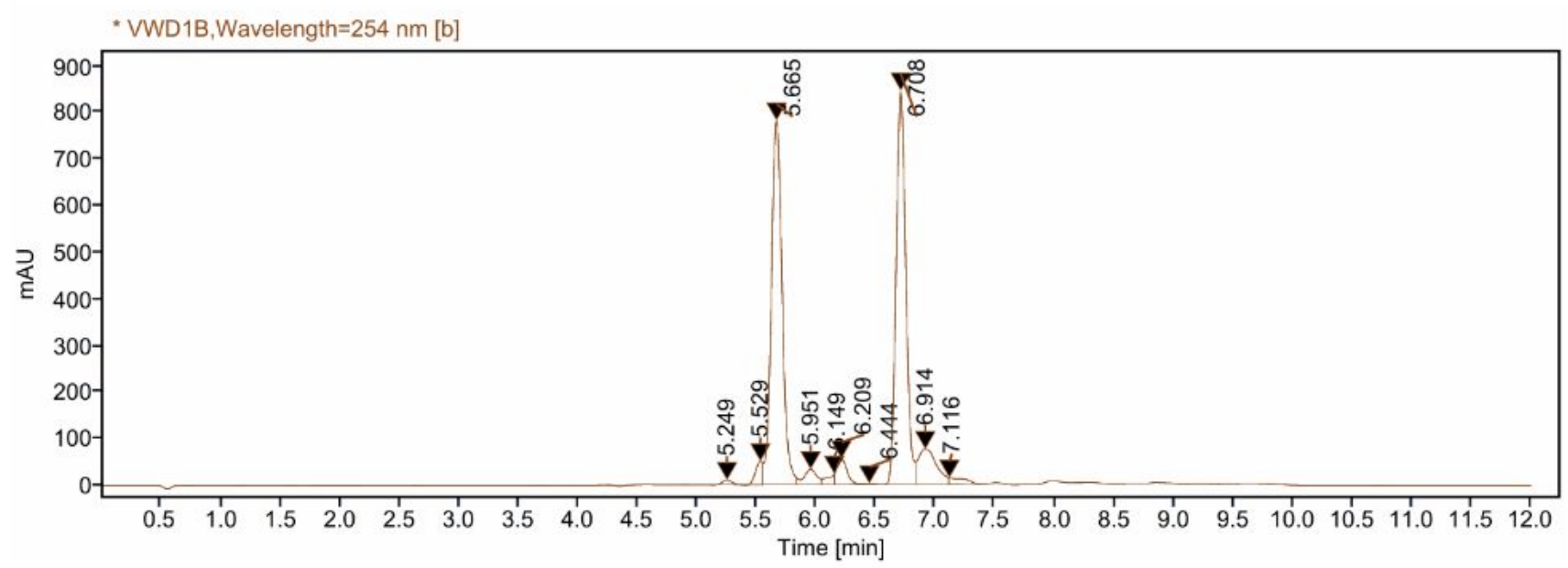

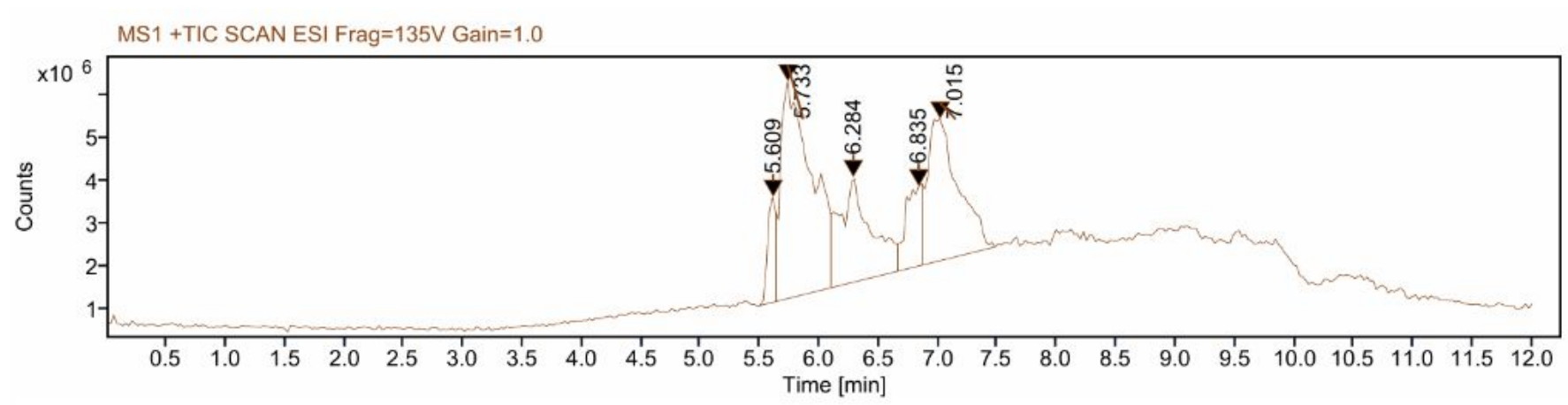

Signal: * VWD1B,Wavelength=254 nm [b]

RT [min]	Area	Area%
5.249	54.5813	0.4699
5.529	190.3067	1.6384
5.665	4913.6943	42.3037
5.951	254.8356	2.1940
6.149	98.4701	0.8478
6.209	331.3254	2.8525
6.444	13.6665	0.1177

RT [min]	Area	Area%
6.708	4824.4791	41.5356
6.914	803.2524	6.9155
7.116	130.6705	1.1250
**Sum**	**11615.2818**	

Peak RT: 5.609 **Peak RT:** 5.733 **Peak RT:** 6.284

Signal: MS1 +TIC SCAN ESI Frag=135V Gain=1.0

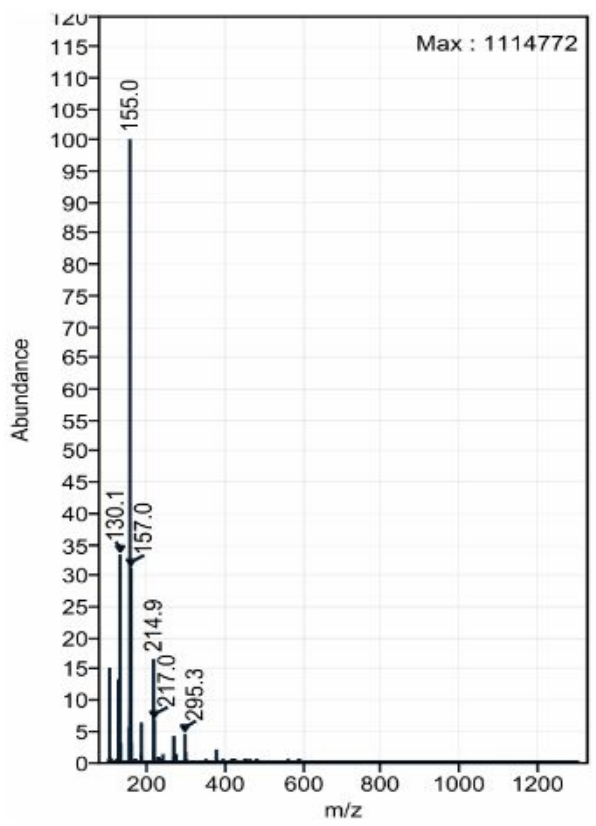

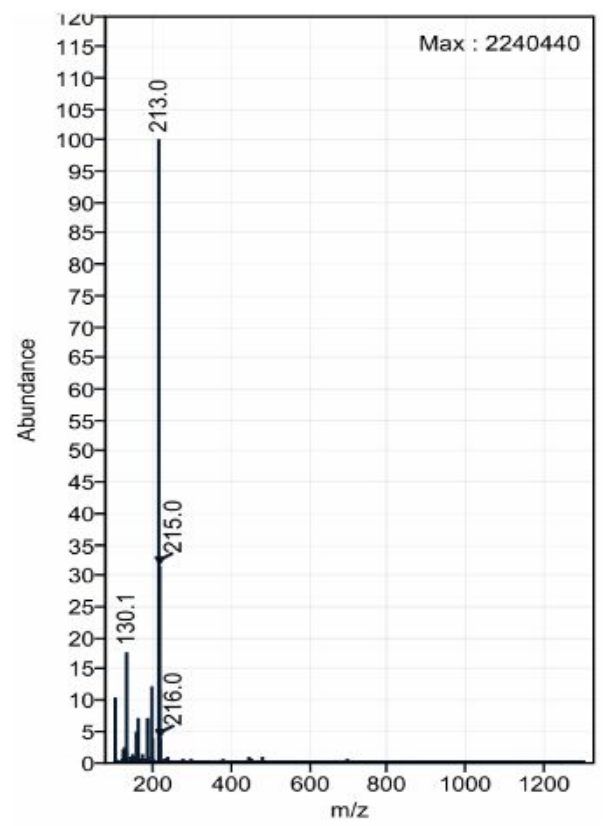

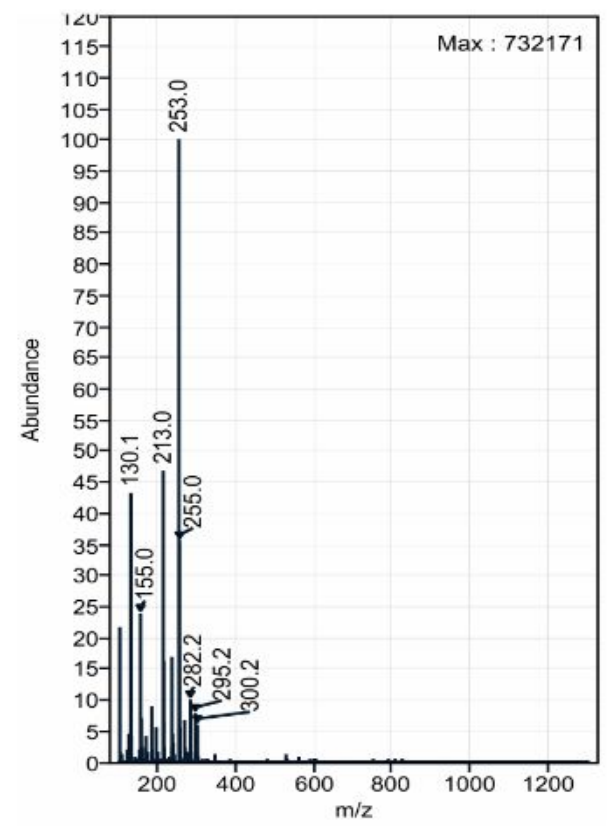

Peak RT: 6.835 **Peak RT:** 7.015

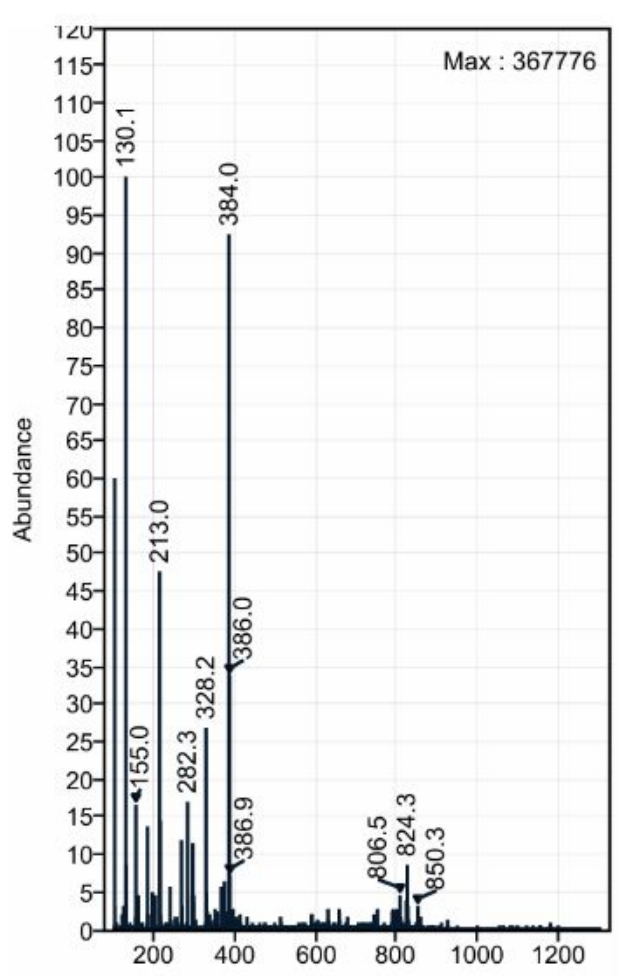

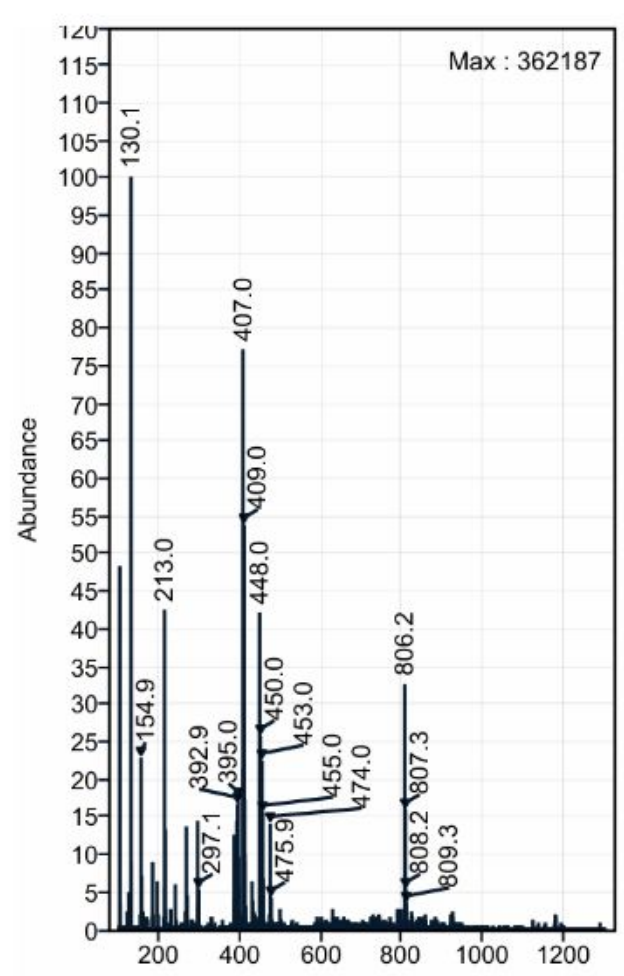

LCMS of intermediate 3b

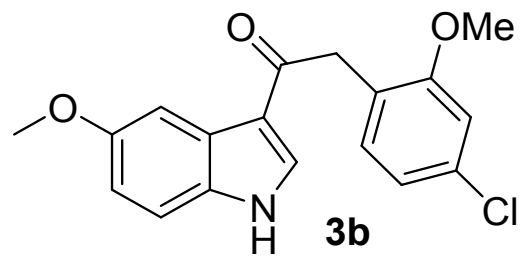

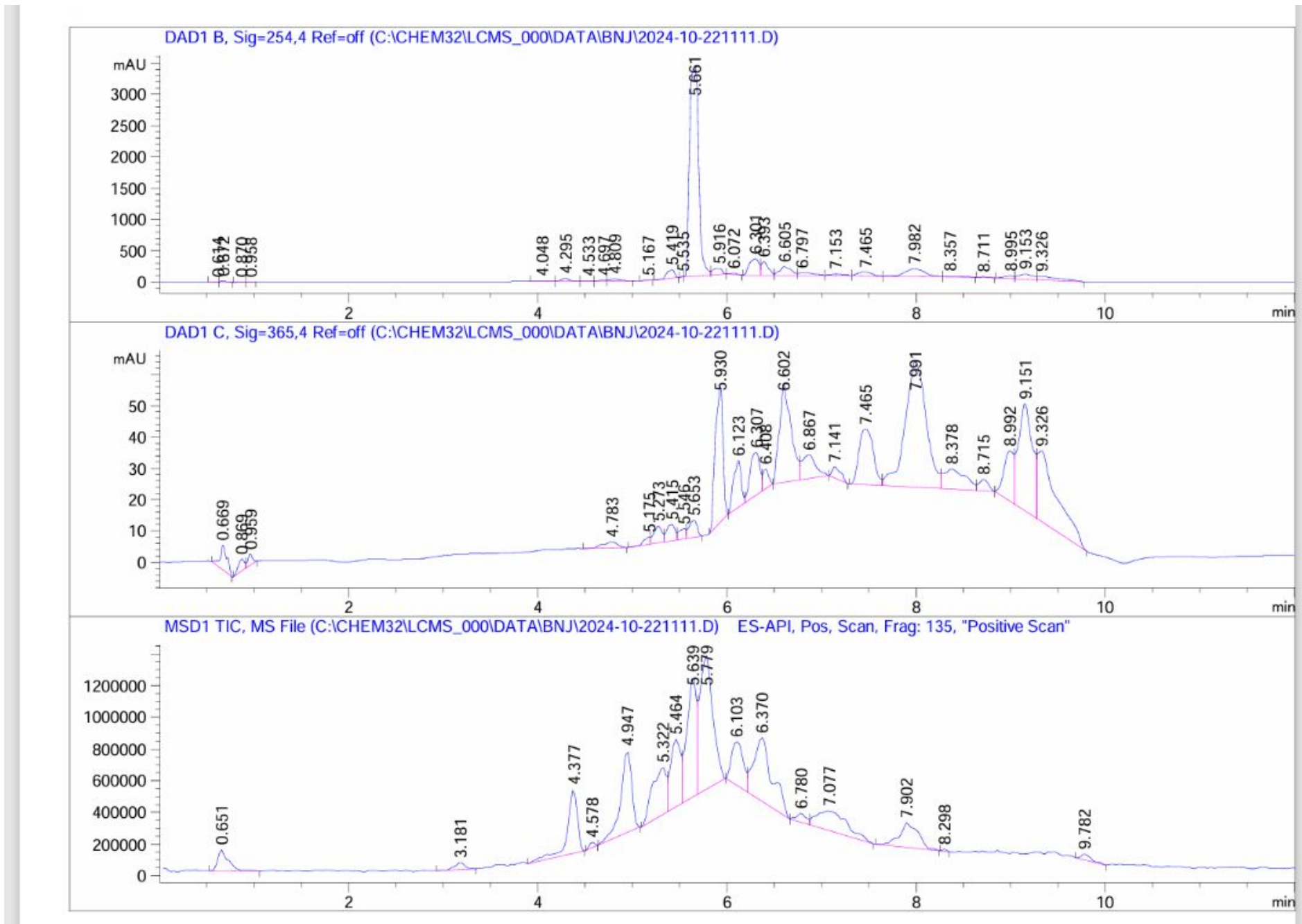

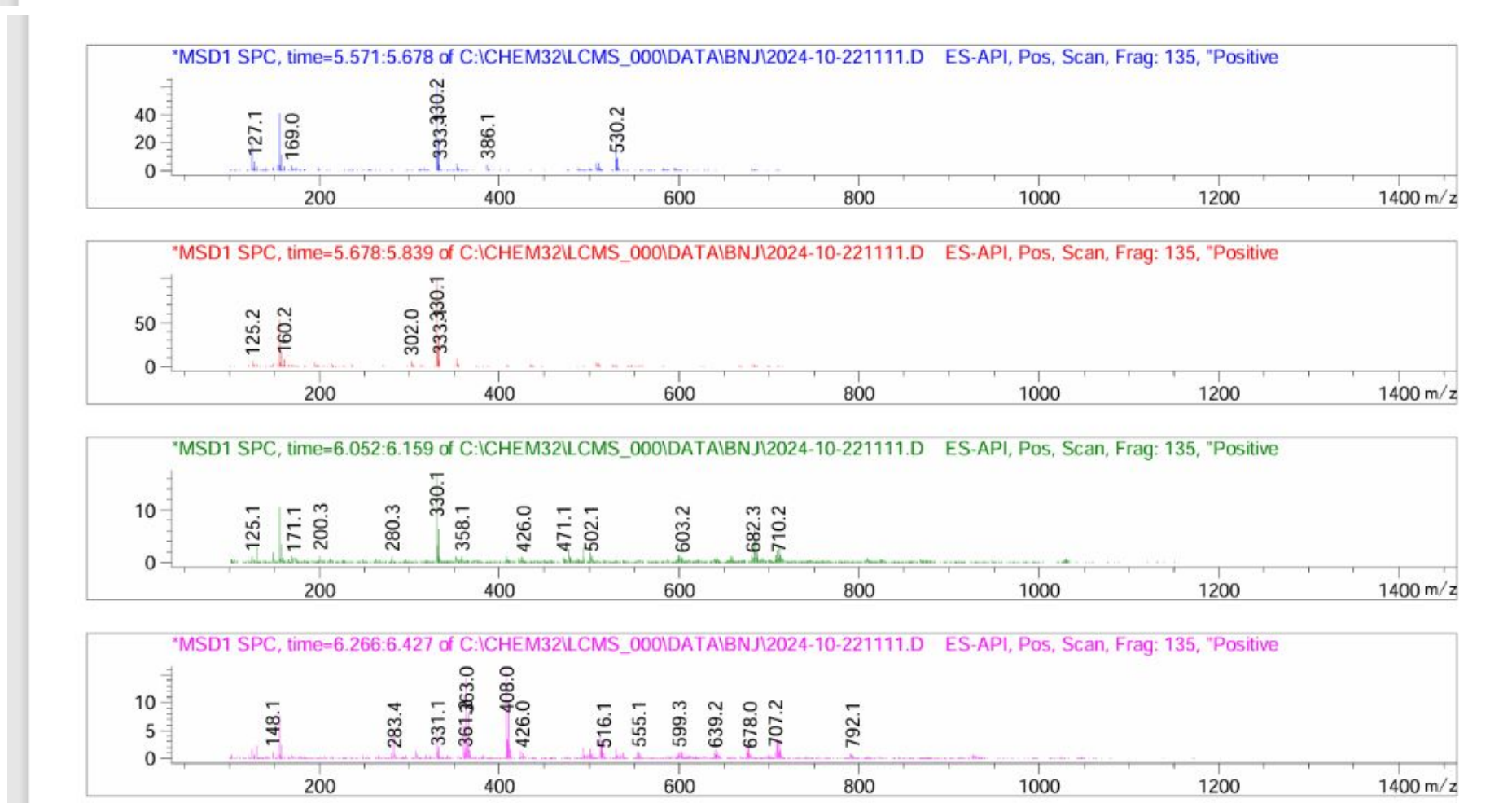

LCMS of intermediate 3c

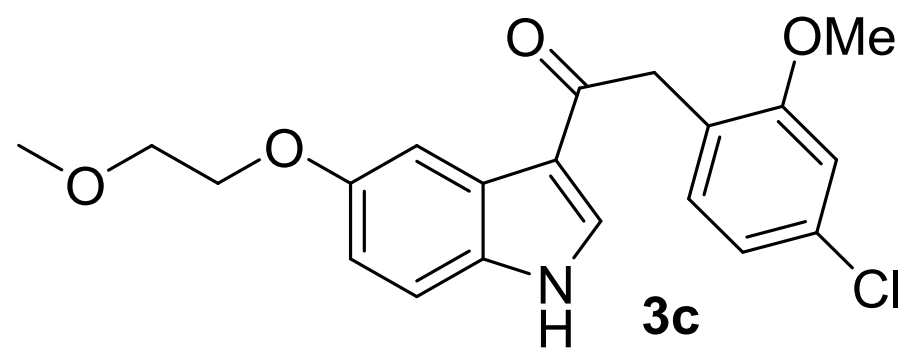

3c

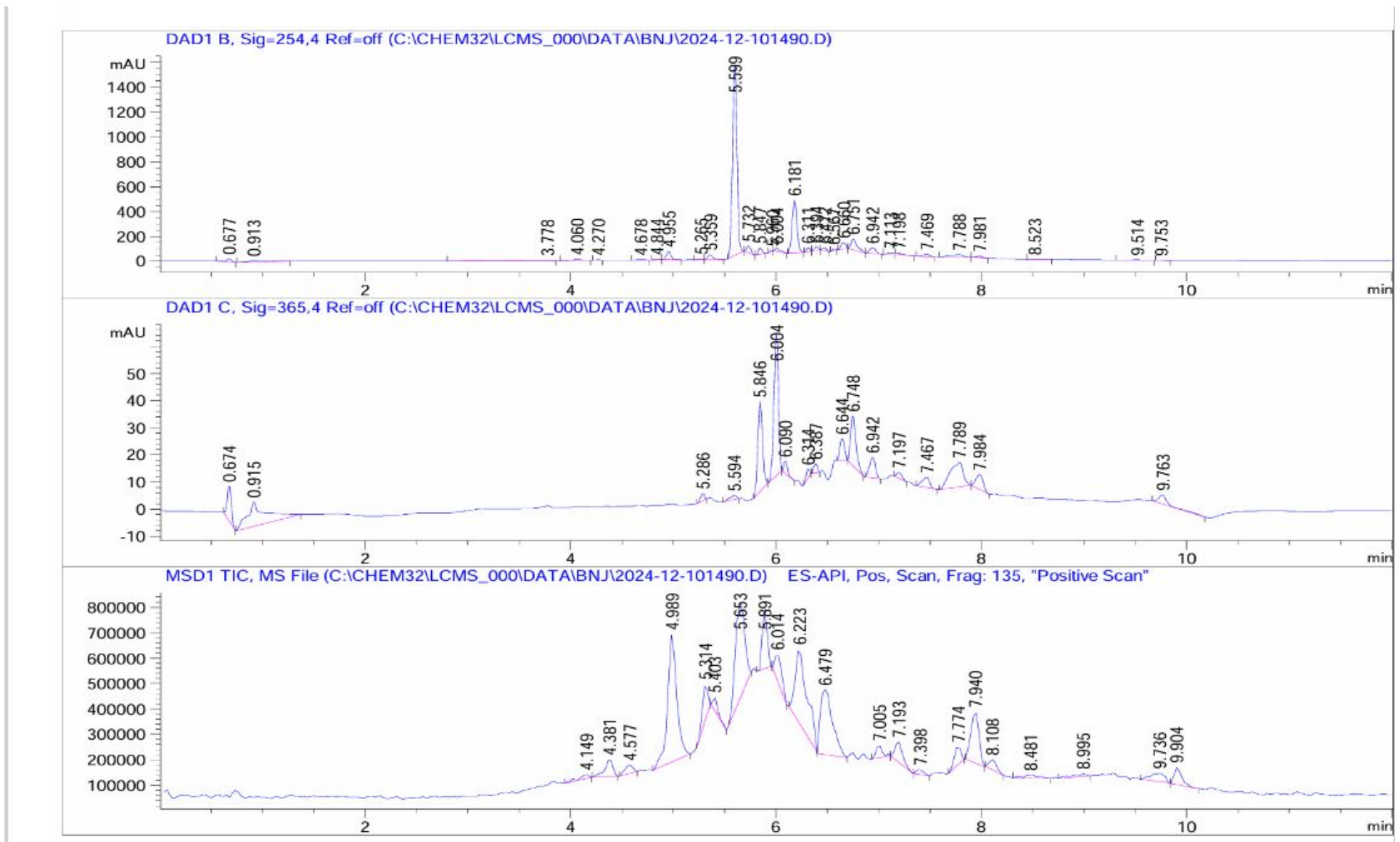

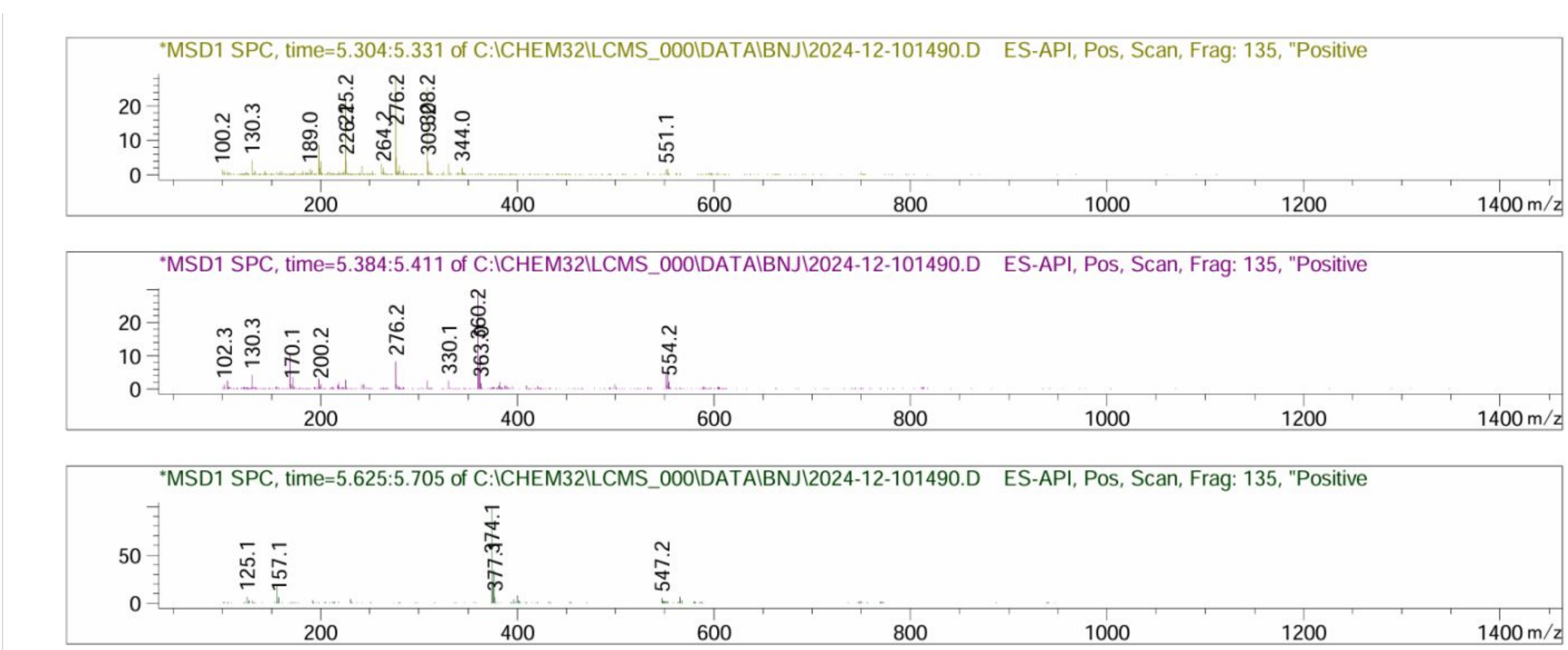

LCMS of intermediate 4a

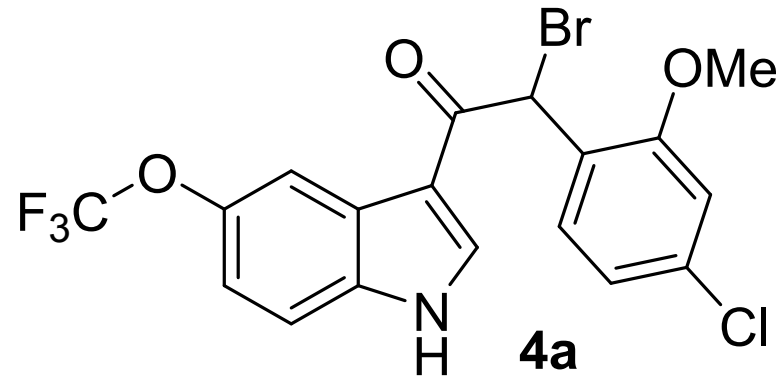

4a

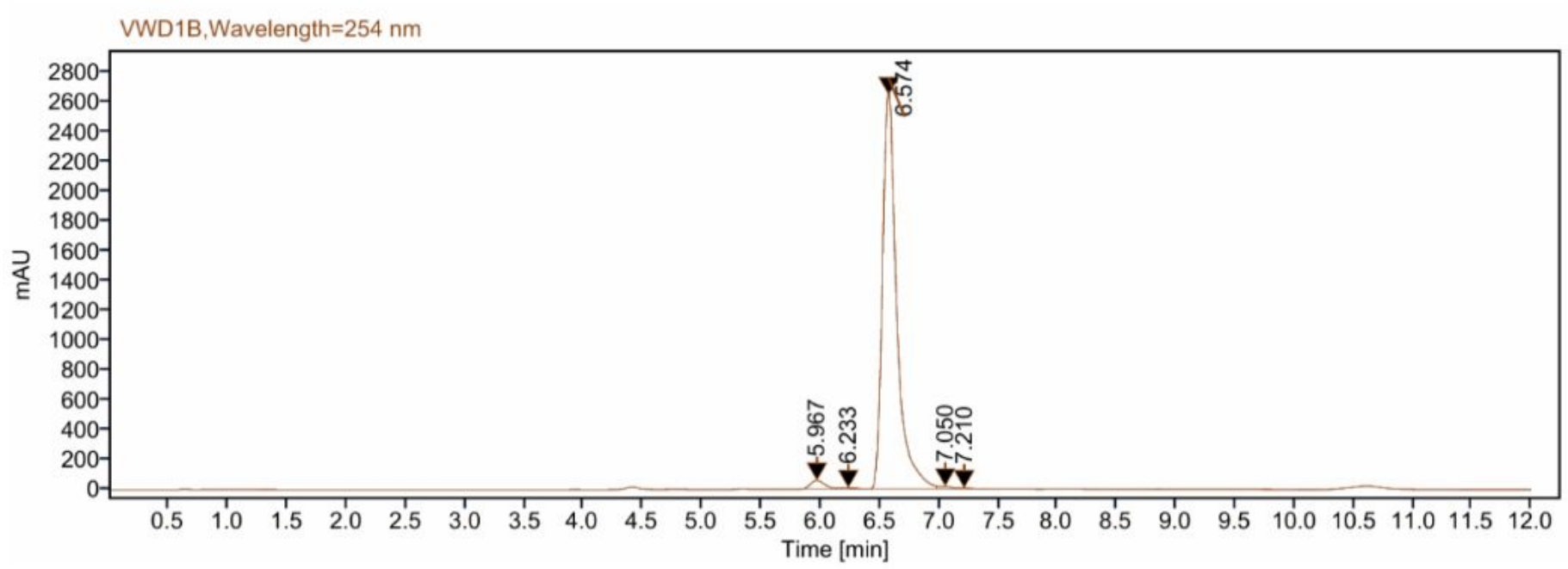

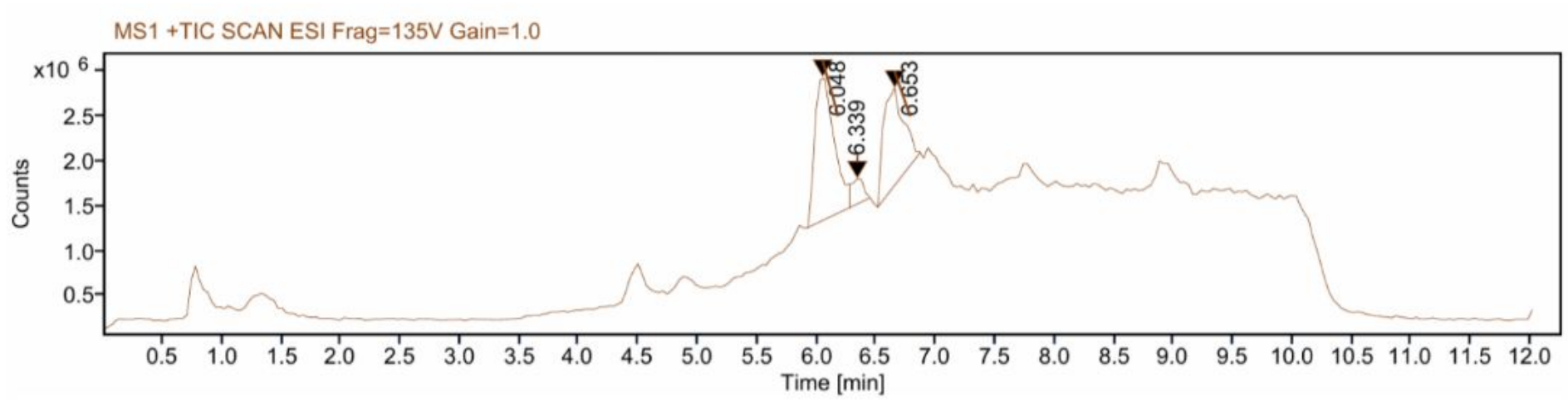

Signal: VWD1B,Wavelength=254 nm

RT [min]	Area	Area%
5.967	480.8370	2.0975
6.233	68.2344	0.2977
6.574	22218.4631	96.9218
7.050	123.3115	0.5379
7.210	33.2573	0.1451
**Sum**	**22924.1033**	

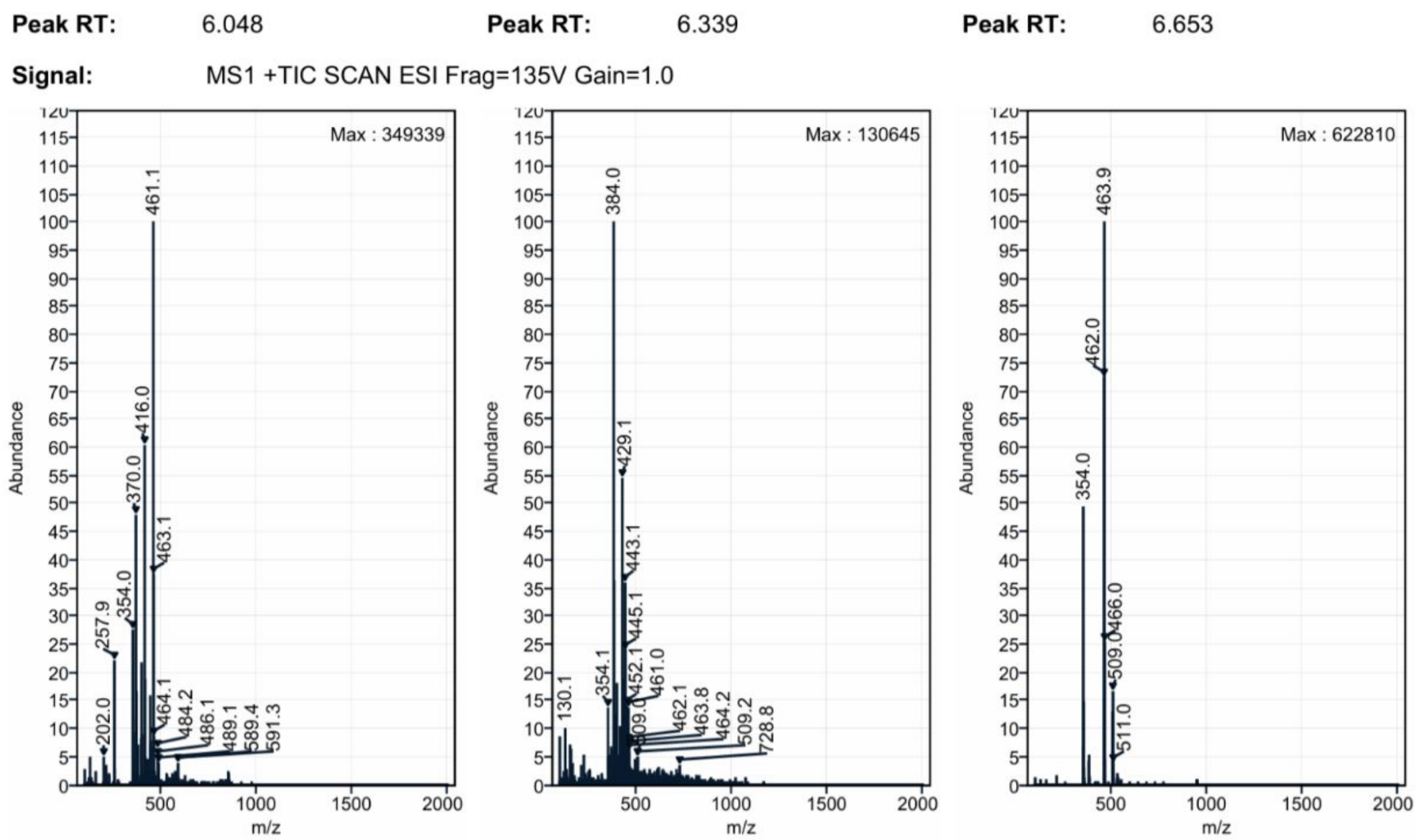

LCMS of intermediate 4b

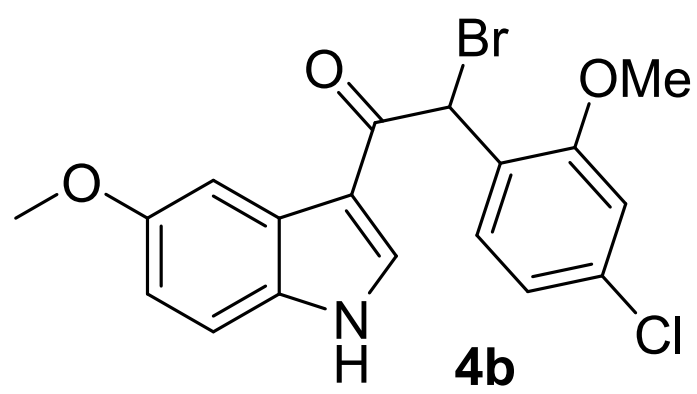

4b

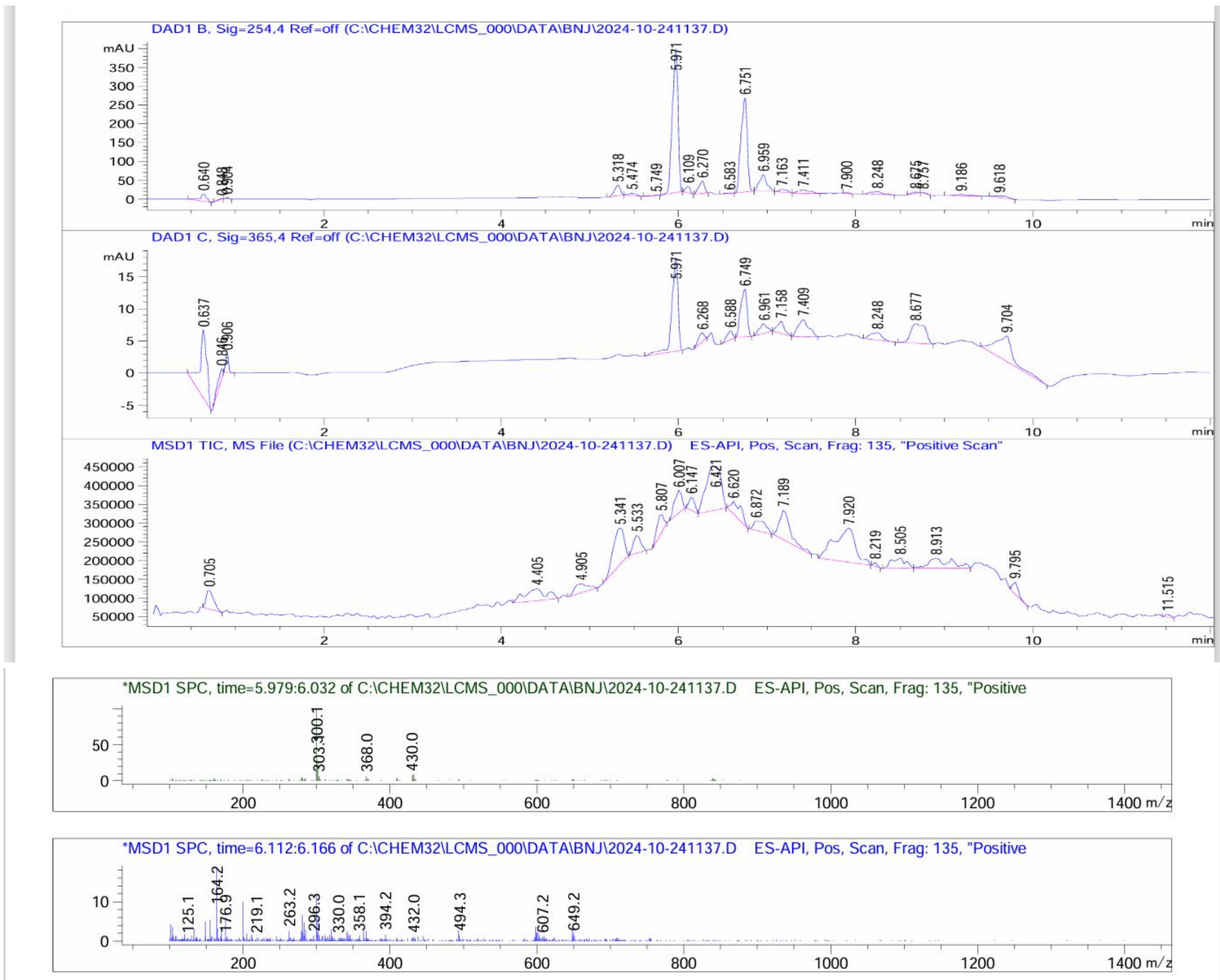

LCMS of intermediate 4c

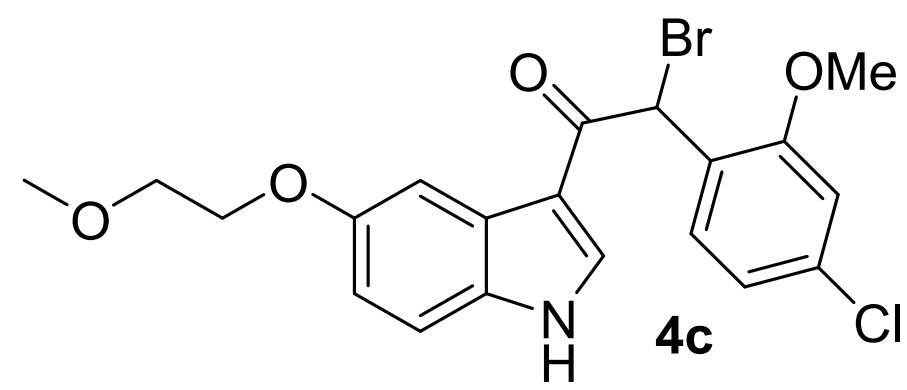

4c

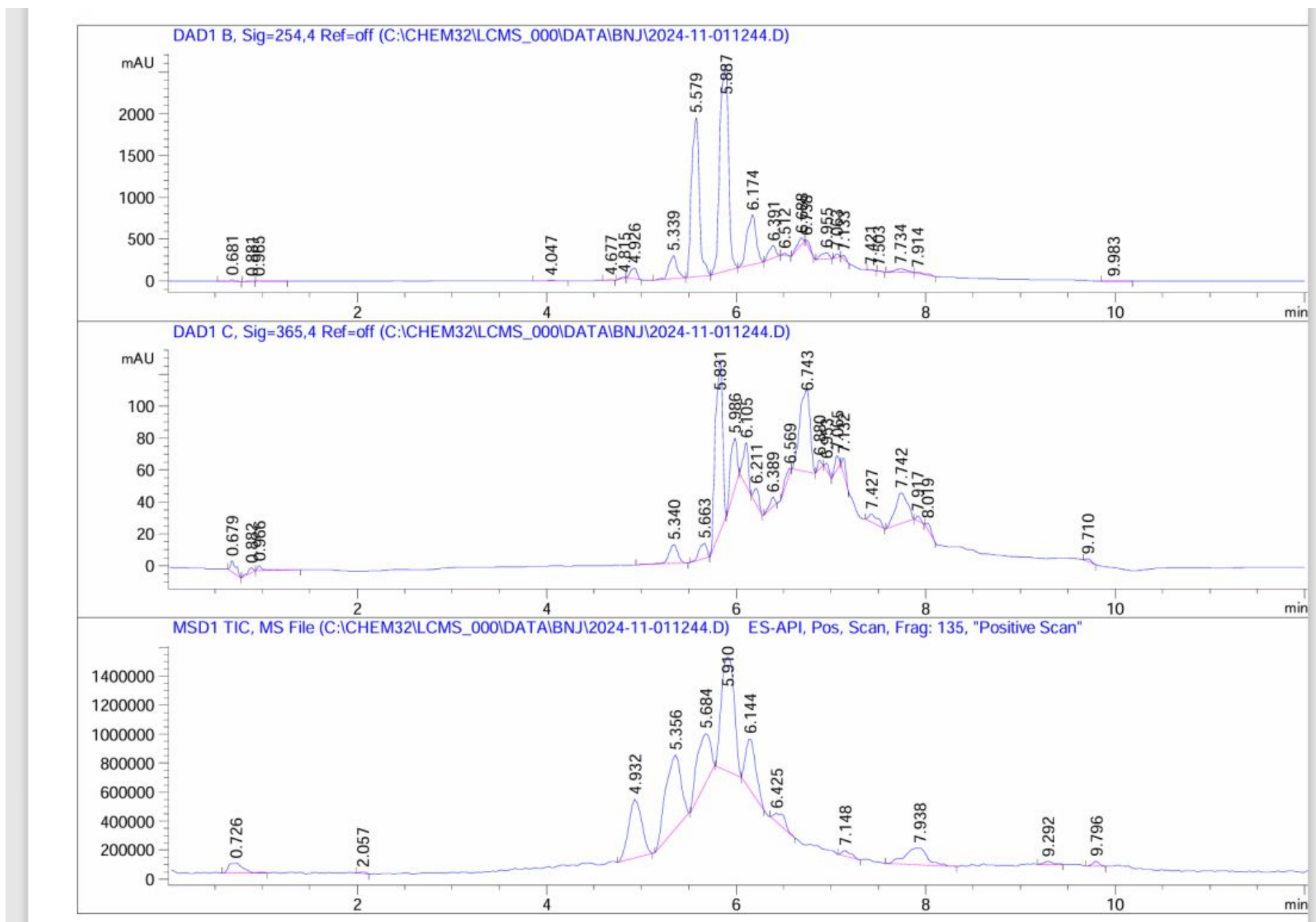

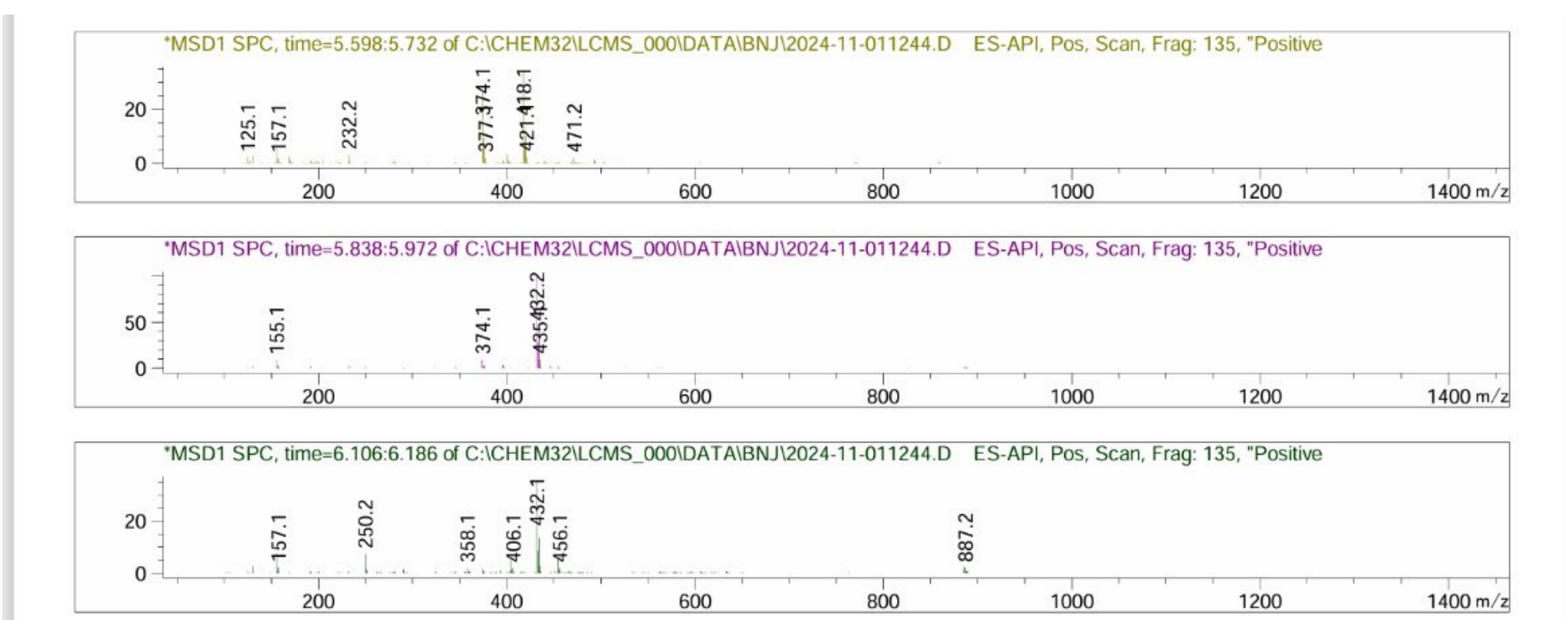

LCMS of intermediate 7

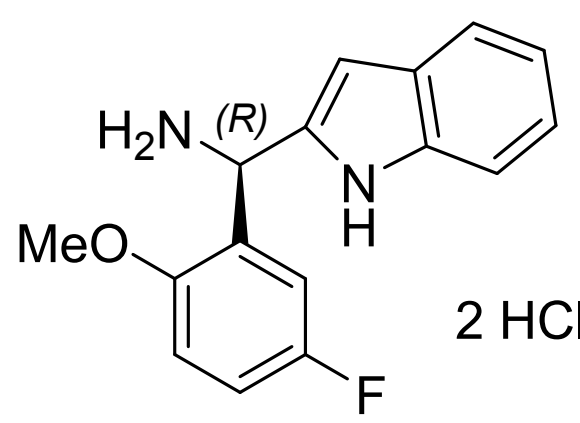

7

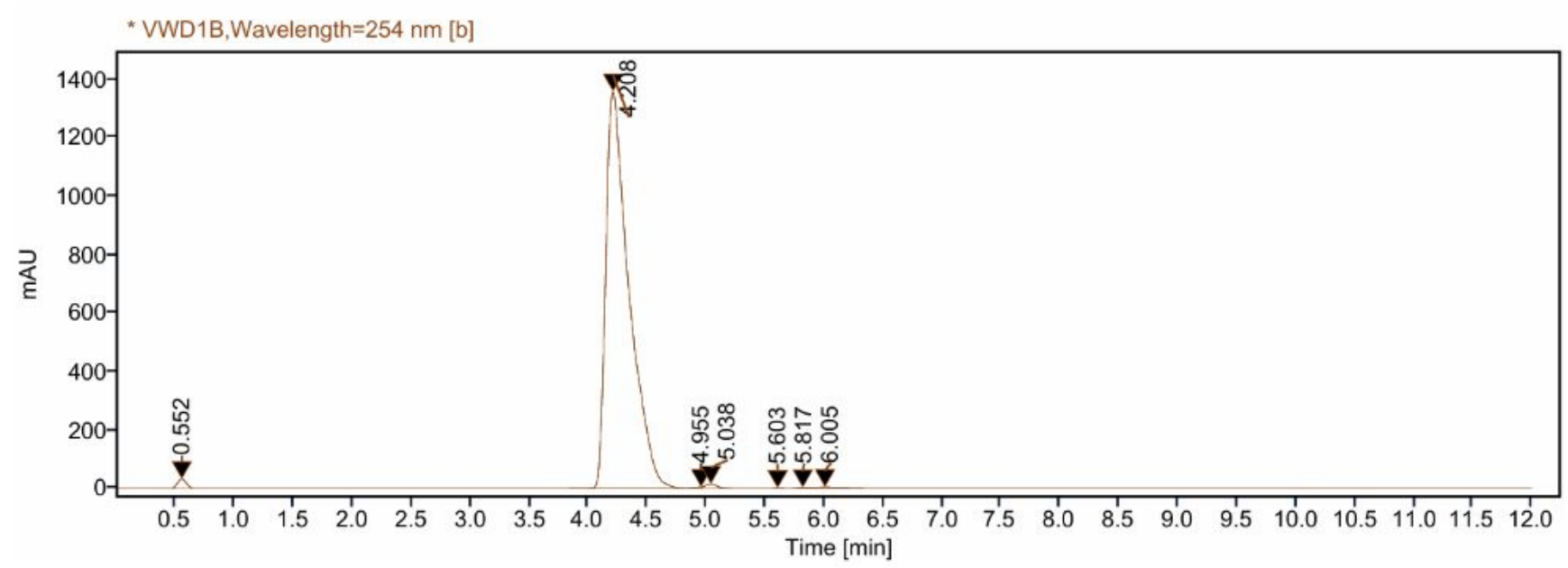

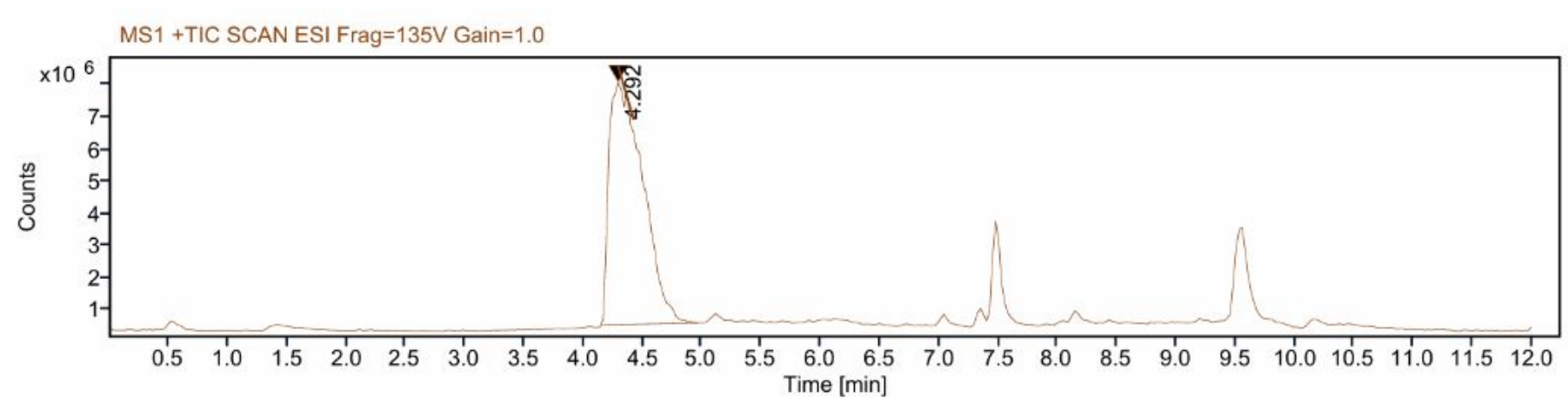

Signal: * VWD1B,Wavelength=254 nm [b]

RT [min]	Area	Area%
0.552	162.9949	0.8628
4.208	18532.7609	98.1016
4.955	16.8956	0.0894
5.038	98.7774	0.5229
5.603	3.4474	0.0182
5.817	15.9067	0.0842
6.005	60.6118	0.3208

Peak RT: 4.292

Signal: MS1 +TIC SCAN ESI Frag=135V Gain=1.0

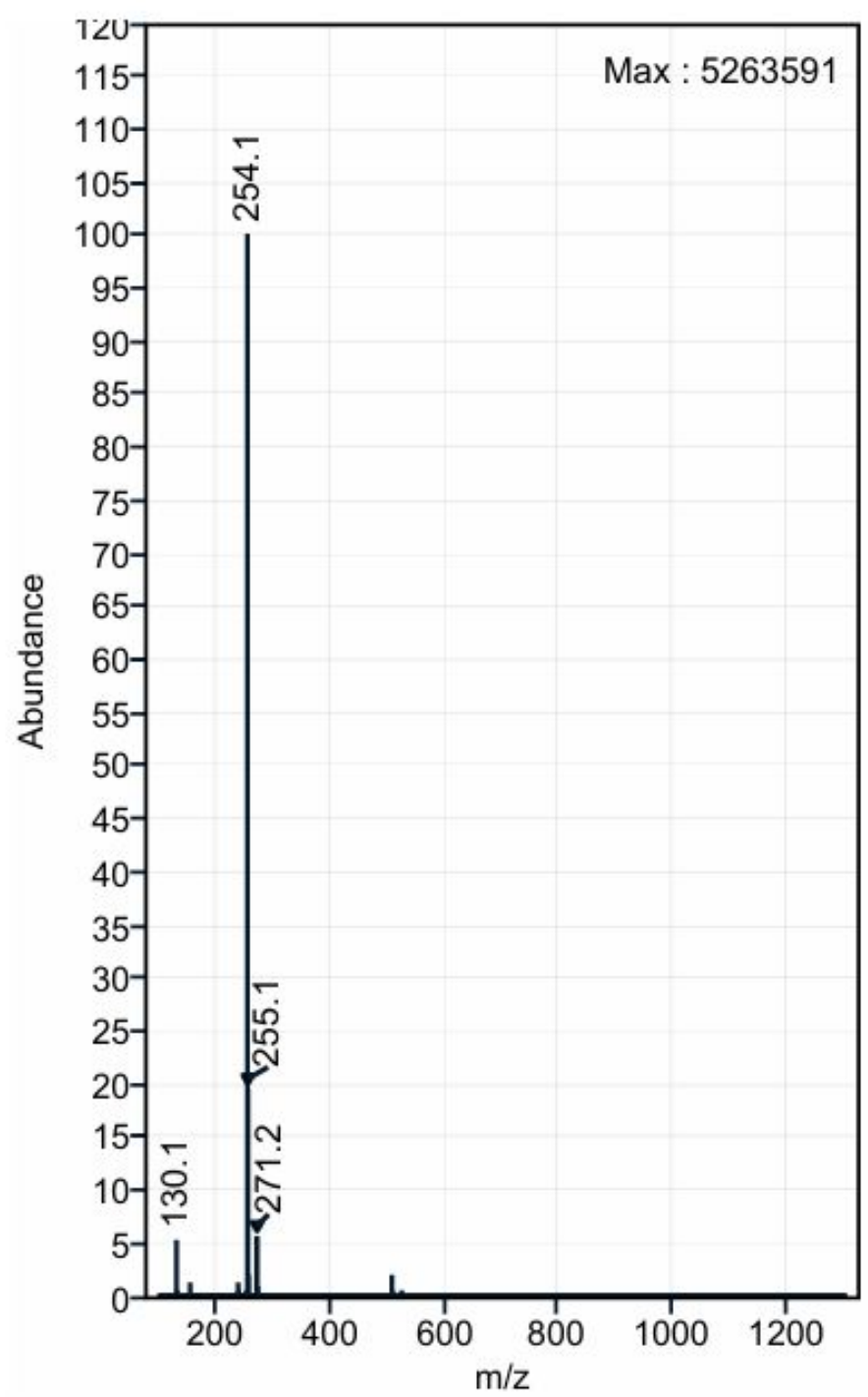

Supplement: Supplementary file 1 [file ci6c02098_si_001.pdf]
